# Characterizing Changes in the Rate of Protein-Protein Dissociation upon Interface Mutation Using Hotspot Energy and Organization

**DOI:** 10.1371/journal.pcbi.1003216

**Published:** 2013-09-05

**Authors:** Rudi Agius, Mieczyslaw Torchala, Iain H. Moal, Juan Fernández-Recio, Paul A. Bates

**Affiliations:** 1 Biomolecular Modelling Laboratory, Cancer Research UK London Research Institute, London, United Kingdom; 2 Joint BSC-IRB Research Program in Computational Biology, Life Science Department, Barcelona Supercomputing Center, Barcelona, Spain; University of Heidelberg, Germany

## Abstract

Predicting the effects of mutations on the kinetic rate constants of protein-protein interactions is central to both the modeling of complex diseases and the design of effective peptide drug inhibitors. However, while most studies have concentrated on the determination of association rate constants, dissociation rates have received less attention. In this work we take a novel approach by relating the changes in dissociation rates upon mutation to the energetics and architecture of hotspots and hotregions, by performing alanine scans pre- and post-mutation. From these scans, we design a set of descriptors that capture the change in hotspot energy and distribution. The method is benchmarked on 713 kinetically characterized mutations from the SKEMPI database. Our investigations show that, with the use of hotspot descriptors, energies from single-point alanine mutations may be used for the estimation of off-rate mutations to any residue type and also multi-point mutations. A number of machine learning models are built from a combination of molecular and hotspot descriptors, with the best models achieving a Pearson's Correlation Coefficient of 0.79 with experimental off-rates and a Matthew's Correlation Coefficient of 0.6 in the detection of rare stabilizing mutations. Using specialized feature selection models we identify descriptors that are highly specific and, conversely, broadly important to predicting the effects of different classes of mutations, interface regions and complexes. Our results also indicate that the distribution of the critical stability regions across protein-protein interfaces is a function of complex size more strongly than interface area. In addition, mutations at the rim are critical for the stability of small complexes, but consistently harder to characterize. The relationship between hotregion size and the dissociation rate is also investigated and, using hotspot descriptors which model cooperative effects within hotregions, we show how the contribution of hotregions of different sizes, changes under different cooperative effects.

## Author Summary

Within a cell, protein-protein interactions vary considerably in their degree of stickiness. Mutations at protein interfaces can alter the interaction between protein pairs, causing them to dissociate faster or slower. This may lead to an alteration in the dynamics of the cellular networks in which these proteins are involved. Therefore, the calculation and interpretation of mutants, which affect the rate of dissociation, is critical to our understanding of complex networks and disease. A key characteristic of protein–protein interfaces is that a subset of residues are responsible for most of the binding energy, such residues are called hotspots and effectively represent the sticky points of the interaction. In this work, we exploit both hotspot energies and organization and use them for the calculation of off-rate changes upon mutations. The insights gained provide us with a clearer understanding of the critical regions of stability and how they change for complexes of different sizes. Moreover, we provide a comprehensive map of the key determinants responsible for the accurate characterization of different classes of mutations, complexes and interface regions. This paves the way for more intelligent computational-interface-design algorithms and provides new insight into the interpretation of destabilizing mutations involved in complex diseases.

## Introduction

Protein-Protein interactions are at the core of all biological systems and the rates at which biomolecules associate and disassociate are the major driving forces behind the complex time-dependent signaling observed in many biological processes. Ordinary Differential Equations (ODEs) are generally used to model these processes [1]–[3]; however, ODEs are bottlenecked by the limited availability of the relevant experimental rate constants [4]. Therefore, the accurate calculation of the kinetic rate constants holds significant application in our understanding of complex networks involved in diseases such as cancer [5]–[7]. Kinetic rate constant prediction is also central to effective drug design [8]–[10]; *in vivo* scenarios, where the concentration of a drug-like ligand exposed to its target receptor is not constant, as usually it is *in vitro*, the drug efficacy is no longer well described by the *in vitro* measured dissociation constant, but rather depends on the association (*k_on_*) and dissociation (*k_off_*) rate constants [8]. Whereas the enhancement of the on-rate is limited by the diffusion rate and several pharmacological factors, off-rate optimization is independent of such factors and entirely dependent on the short-range interactions between the bound monomers in question [8]. Hence the calculation and minimization of dissociation rate constants becomes a critical objective in drug design optimization [11]. At the other end of the spectrum, most disease causing mutations which are not in the protein core, occur at the interface regions and result in complex destabilization [12] and a number of studies have shown that changes in the binding free energy are largely the result of changes in the off-rate as opposed to minimal changes in the on-rate [13], [14]. While several aspects of biomolecular association have been investigated [10], [15]–[17], work on dissociation rate is still very limited [18]. Moreover, up to the analysis reported in this work, which attempts to calculate off-rate variations upon mutations in a high throughput context, calculation of dissociation rate constants has been limited to *wild-type* complex studies [19], [20].

The *k_off_* of a complex may be estimated using Molecular Dynamic (MD) simulations starting from the bound structure and allowing for dissociation to occur [21]. MD simulations typically allow simulation times of ns to μs, which are below the time-scales necessary for natural dissociation. Although steered molecular dynamics (SMD) simulations provide an alternative means to estimate the dissociation of protein complexes [21]–[23], such methods bias the dissociation process through a forced pathway in the direction of the applied force, and computational complexity limits their applicability. In our recent work, the *wild-type* kinetic rate constants of a number of complexes were predicted, using empirical scoring functions, with a number of molecular descriptors, describing various aspects of protein-protein interaction [19]. Whereas many descriptors showed high correlations with the association rate, particularly those calculated using the unbound structures, significant correlations for the dissociation rate could not be found.

Given the limited predictive ability of the current molecular features for *k_off_* [19], instead of trying to characterize off-rate mutations directly using such molecular features only, a different approach is taken here, one which exploits the synergistic and distributional information available in hotspot residues. Hotspots refer to a subset of residues at the interface which are able to significantly destabilize the binding free energy by more than 2 kcal/mol when mutated to alanine [24]. So far hotspot research has mainly focused on their identification [25]–[35], residue-level properties [24], [36] and distributional properties [37]–[39]. However, work on their practical application is still very limited [40]. Here, the relationship between hotspot energetics and the dissociation rate constant is investigated. We put to test the notion of whether the *ΔΔG*s of single-point mutations to alanine, as traditionally trained upon and predicted by hotspot prediction algorithms, can be used to quantify changes in Δ*k_off_*. The key point of interest here is that mutations, such as those we would like to quantify the changes in off-rate for, are not limited to single-point alanine mutations, as are in hotspot prediction algorithms. Therefore, a direct estimation of Δ*k_off_* using *ΔΔG* will not suffice. To address this, an unconventional approach is taken and computational alanine scans of the interface pre- and post-mutation are performed using hotspot predictor algorithms. Using the *ΔΔG*s of the single-point alanine mutations generated from these scans, a set of 16 hotspot descriptors are designed and calculated. The hotspot descriptors are then used as features to quantify off-rate changes of single-point, and more importantly multi-point, mutations to alanine and also non-alanine. A key advantage of using such hotspot descriptors, is not only the fact that non-alanine and multi-point mutations can now be characterized using single-point alanine mutations, but higher-order or rather, global effects of a given mutation can now be addressed. These include changes in the size and distribution of hotregions (clusters of hotspots), cooperative effects within hotregions and changes in localized stability regions such as the core, rim and support regions. All of which, as shown in this work, play varyingly important roles in the determination of the off-rate of a given mutation.

Our results confirm that indeed, using hotspot descriptors, the energies of single-point mutations to alanine can be used to describe effects of mutations other than alanine and also multi-point mutations. Machine learning models using such hotspot descriptors show consistently higher predictive abilities in the fine-grained and coarse-grained prediction of off-rate changes upon mutation, than models without hotspot descriptors. We find that hotspot descriptors tend to be broadly predictive for different classes of mutations, whereas molecular descriptors can be highly specific to small subsets of mutations. Our investigations also highlight differences in the distribution of stable regions at the interface for complexes off different sizes and interface areas and show the effects of cooperativity, on the stability provided by hotregions of various sizes.

### Approach

In the first part of this work, sets of hotspot descriptors are generated, where each set represents hotspot descriptors generated from a particular hotspot predictor. The hotspot predictors tested include; two hotspot prediction servers (*KFC2* [30] and *Hotpoint* [28]) and also two hotspot predictors developed in this work (*RFSpot* and *RFSpot_KFC2*). The hotspot descriptors' ability to characterize changes in off-rate due to mutations is assessed on a set of 713 experimental off-rates taken from wild-type and mutated proteins in the SKEMPI database [41]. Experimental off-rates in the dataset cover a range of Δlog_10_(*k_off_*) of −8.5 to 6.8, with *k_off_* units of s−1, and represent a diverse set of interactions as listed in the Supplementary Information (Dataset S1). As a relative performance measure, a benchmark set of 110 molecular descriptors (Text S1) is also included in the analysis and compared to the performance of the hotspot descriptors. The molecular descriptor set consists of a complex and comprehensive set of structure related descriptors characterizing various aspects of protein-protein interactions and their energetics; a subset of which have already proven to be successful in our previous work on predicting *wild-type* protein-protein binding free energies and kinetic rate constants [19], [42] and therefore serves as a thorough benchmark comparison. All descriptor analysis in the initial section is independent of any machine learning models trained on off-rate data. Rather, the aim here is to uncover the individual predictive power of each descriptor in estimating off-rate mutations. The Pearson's Correlation Coefficient (PCC) is used to evaluate fine-grain predictive ability, *i.e.* the ability to make numerical predictions. On the other hand, the Mann Whitney U-Test and several classification measures described in Materials and Methods are used to evaluate the coarse-grain ability to detect stabilizing mutations from neutral and destabilizing mutations.

In the second part, the prediction of off-rates using machine learning models is investigated. Here, several models using both hotspot and molecular descriptors are built, and their prediction patterns and anomalies highlighted. In order to uncover similarities in their predictions, the 713 off-rate dataset is categorized into what we term as data regions. Such data regions represent mutations that have a common physical property, or come from a similar type of complex or region on the interface. Mutations within a data region in turn might hold different predictive difficulty than mutations in another. This data region analysis enables us to identify current strengths in the prediction of off-rates and conversely, mutations which are consistently harder to characterize.

In the third part of this work, the use of specialized models specific to different data regions is investigated. By doing so we are able to identify descriptors of which their predictive value is highly specific to subsets of mutations, regions on the interface, or types of complexes. The specialized models are generated using a Genetic Algorithm running Feature Selection (GA-FS) with either linear (Linear Regression, LR) or non-linear (Support Vector Machines, SVM) learning models.

In the latter sections, the effects of complex size and interface area on the distribution of stability regions at the interface are investigated. Issues related to cooperativity and conformational changes, in the determination of off-rates, are also highlighted.

## Results/Discussion

### Hotspot descriptor generation

**Figure 1:**
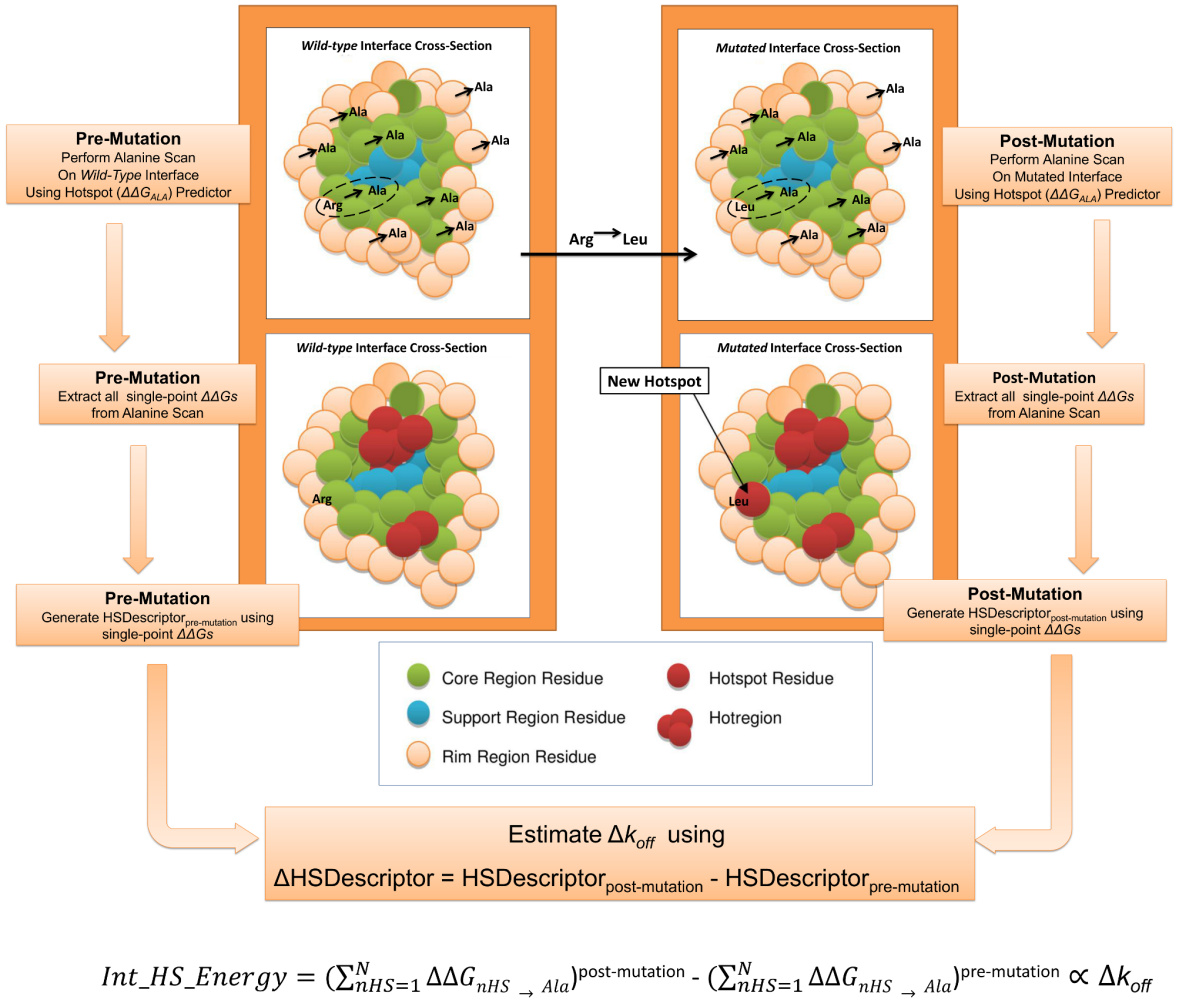
Off-rate estimation using hotspot energies and organization. In this work we generate a set of hotspot descriptors for characterizing off-rate changes upon mutation. The hotspot descriptors use single-point alanine *ΔΔG*s from computational alanine-scans generated using hotspot prediction algorithms, to predict changes in off-rate upon single-point and multi-point mutations to all residue types. To do so, for a given *wild-type* complex structure, the interface is scanned for hotspots using a hotspot prediction algorithm. The single-point alanine *ΔΔG*s from the scan are extracted and stored. Next, the structural mutation in question is applied and the mutated interface re-scanned for hotspots. This generates a new set of single-point alanine *ΔΔG*s for the mutated interface. Note that the mutation in question may also affect the hotspot energies of other neighboring residues which are not mutated. The two sets of *ΔΔG*s are then used to generate a set of hotspot descriptors, where the final hotspot descriptor value is the change in the descriptor's value from mutant to *wild-type*. For example in the case of *Int_HS_Energy*, the final value is the change in the sum of the *ΔΔG*s, of all hotspot residues, pre- and post-mutation. Hotspots are also categorized into core, rim, support and hotregions. This enables us to investigate and account for cooperative effects within hotregions and to identify differences in regions critical for stability, both on complexes of different size and interface area.

One of the main motivations behind this work is to explore the use of currently available descriptors (physics-based and knowledge-based potentials) and design a new class of descriptors (hotspot descriptors) for describing changes in off-rates. On the design of a new class of descriptors, our proposition is that interface hotspots can be seen as the anchor points responsible for the stable longevity of a complex. Namely, changes in the number of hotspots, hotspot energies and their distribution across the interface brought upon by structural mutations directly relates to changes in off-rate. Our approach of using hotspot predictions and subsequently hotspot descriptors for characterizing off-rates is depicted in Figure 1. First a pre-mutation alanine scan is performed; essentially this translates to using a hotspot predictor of choice on each residue at the interface. This generates a collection of single-point alanine *ΔΔG*s that are then employed differently depending on the hotspot descriptor in question (See Table 1). For example if we are using *Int_HS_Energy*, then this hotspot descriptor will sum all the energies of only the hotspot residues. After all the hotspot descriptors for the *wild-type* complex are calculated, the mutation in question is applied using FoldX [43], such as the Arg to Leu mutation in Figure 1. Then, using a hotspot predictor as in the *wild-type* scan, another computational alanine scan is performed on the mutated interface. Again, all single-point alanine *ΔΔG*s are then fed into the hotspot descriptors. Continuing with the example of *Int_HS_Energy* as a hotspot descriptor, here the *ΔΔG*s of only the hotspot residues on the mutated interface are summed, and the final descriptor value will be the change in the sum of the single-point *ΔΔG*s to alanine of all hotspot residues pre- and post-mutation. This value is then correlated to Δ*k_off_*_Leu→Arg_.

**Table 1:** Summary of hotspot descriptors.

Hotspot descriptor	Description
**Int_Energy_1**	Change in Total Interface *ΔΔG*_ALA_ Energy
**Int_HS_Energy**	Change in Total Interface *ΔΔG*_ALA_ Energy of Hotspots
**No_HS**	Change in Number of Hotspots
**No_Clusters**	Change in Number of Unique Hotregions
**MaxClusterSize**	Change in Number of Hotspots in Largest Hotregion
**AVG_HS_PathLength**	Change in Hotspot Coverage
**CoreHSEnergy**	Change in Total *ΔΔG*_ALA_ Energy of Hotspots in Core Region
**CoreHS**	Change in Number of Hotspots in Core Region
**RimHSEnergy**	Change in Total *ΔΔG*_ALA_ Energy of Hotspots in Rim Region
**RimHS**	Change in Number of Hotspots in Rim Region
**SuppHSEnergy**	Change in Total *ΔΔG*_ALA_ Energy of Hotspots in Support Region
**SuppHS**	Change in Number of Hotspots in Support Region
**HSEner_PosCoop**	Change in Total Hotspot *ΔΔG*_ALA_ Energy Accounting for Positive Cooperativity in Hotregions
**HS_PosCoop**	Change in Hotspot Counts Accounting for Positive Cooperativity in Hotregions
**HSEner_NegCoop**	Change in Total Hotspot *ΔΔG*_ALA_ Energy Accounting for Negative Cooperativity in Hotregions
**HS_NegCoop**	Change in Hotspot Counts Accounting for Negative Cooperativity in Hotregions

### Hotspot descriptors and hotspot predictors

The motivations and calculation for each of the 16 hotspot descriptors is detailed in Materials and Methods. In summary (See Table 1); *Int_HS_Energy*, is the difference in the sum all the energies of hotspot residues pre- and post-mutation. *HSEner_PosCoop* and *HSEner_NegCoop* are identical to *Int_HS_Energy* except that, in order to account for positive and negative cooperativity effects between hotspots within a hotregion, the hotspot energies are down-weighted and up-weighted accordingly to the size of hotregion they are in. *CoreHSEnergy*, *RimHSEnergy* and *SuppHSEnergy*, are similar to *Int_HS_Energy*, except that changes in hotspot energies are limited to the given region on the interface. Each of the 6 descriptors also has its coarse-grain counterpart (*No_HS*, *HS_PosCoop*, *HS_NegCoop*, *CoreHS*, *RimHS* and *SuppHS*), where only hotspot counts instead of energies are used in the calculations. Other hotspot descriptors include the change in the size of the largest hotregion (*MaxClusterSize*), the number of hotregions (*No_Clusters*), the spread of the hotspots at the interface (*AVG_HS_PathLength*) and *Int_Energy_1* that characterizes changes in all single-point alanine mutations at the interface.

A number of hotspot predictors are investigated for the generation of hotspot descriptors, and in total 6 sets of hotspot descriptors are generated (See Table 2). These include hotspot descriptors generated from available hotspot prediction servers, *KFC2a*, *KFC2b* [30], *RFHotpoint1* and *RFHotpoint2* [28], along with the hotspot descriptors generated from hotspot prediction algorithms developed in this work (*RFSpot*, *RFSpot_KFC2*). Explanation of each hotspot prediction algorithm, its features, and performance comparisons can be found in Materials and Methods. In summary, *KFC2a* and *KFC2b* are SVM hotspot prediction models developed in [30] and use a combination of solvent accessibility and plasticity features. *RFHotpoint1* and *RFHotpoint2* are random forest models using the features from the original Hotpoint [28] hotspot predictor, but re-trained on a larger dataset from SKEMPI (Table S16 in Text S4). *RFSpot* is a random forest model that employs a large set of molecular descriptors and *RFSpot_KFC2* adds to this feature set, features from the original *KFC2a* and *KFC2b* models. The use of multiple hotspot predictors enables us to probe consistencies and anomalies in the predictive abilities of the hotspot descriptors.

**Table 2:** Pearson's Correlation Coefficient (PCC) of hotspot descriptors with experimental Δlog_10_(*k_off_*) for the 713 off-rate mutations in SKEMPI.

Hotspot descriptor	RFHotpoint1	RFHotpoint2	KFC2a	KFC2b	RFSpot	RFSpot_KFC2	Mean PCC	Variance in PCC
**Int_Energy_1**	−0.312	−0.312	−0.472	−0.432	−0.182	−0.289	−0.333	0.105
**No_HS**	−0.433	−0.266	−0.429	−0.496	−0.493	−0.496	−0.436	0.089
**Int_HS_Energy**	−0.568	−0.312	−0.546	−0.527	−0.532	−0.559	−0.508	0.097
**No_Clusters**	0.101	−0.069	−0.075	−0.272	−0.284	−0.285	−0.147	0.159
**MaxClusterSize**	−0.225	0.022	0.094	0.052	−0.163	−0.292	−0.085	0.162
**AVG_HS_PathLength**	−0.152	−0.139	−0.031	−0.197	−0.110	−0.016	−0.108	0.071
**CoreHSEnergy**	−0.608	−0.365	−0.369	−0.427	−0.541	−0.560	−0.479	0.105
**RimHSEnergy**	−0.415	0.020	−0.100	0.000	−0.367	−0.329	−0.198	0.194
**SuppHSEnergy**	−0.153	−0.162	−0.617	−0.489	−0.385	−0.465	−0.379	0.187
**CoreHS**	−0.413	−0.281	−0.232	−0.476	−0.342	−0.440	−0.364	0.095
**RimHS**	−0.319	−0.071	−0.181	0.000	−0.128	−0.176	−0.146	0.109
**SuppHS**	−0.156	−0.153	−0.430	−0.344	−0.480	−0.441	−0.334	0.146
**HSEner_NegCoop**	−0.487	−0.282	−0.475	−0.260	−0.414	−0.514	−0.405	0.109
**HS_NegCoop**	−0.330	0.013	−0.049	−0.356	−0.415	−0.460	−0.266	0.198
**HSEner_PosCoop**	−0.278	−0.192	−0.218	−0.437	−0.573	−0.444	−0.357	0.150
**HS_PosCoop**	−0.013	−0.256	−0.138	−0.154	−0.494	−0.457	−0.252	0.190

### Off-rate changes of single-point and multi-point mutations can be explained using hotspot energies of single-point alanine mutations

#### Contribution of k_on_ and k_off_ to the binding free energy

**Figure 2:**
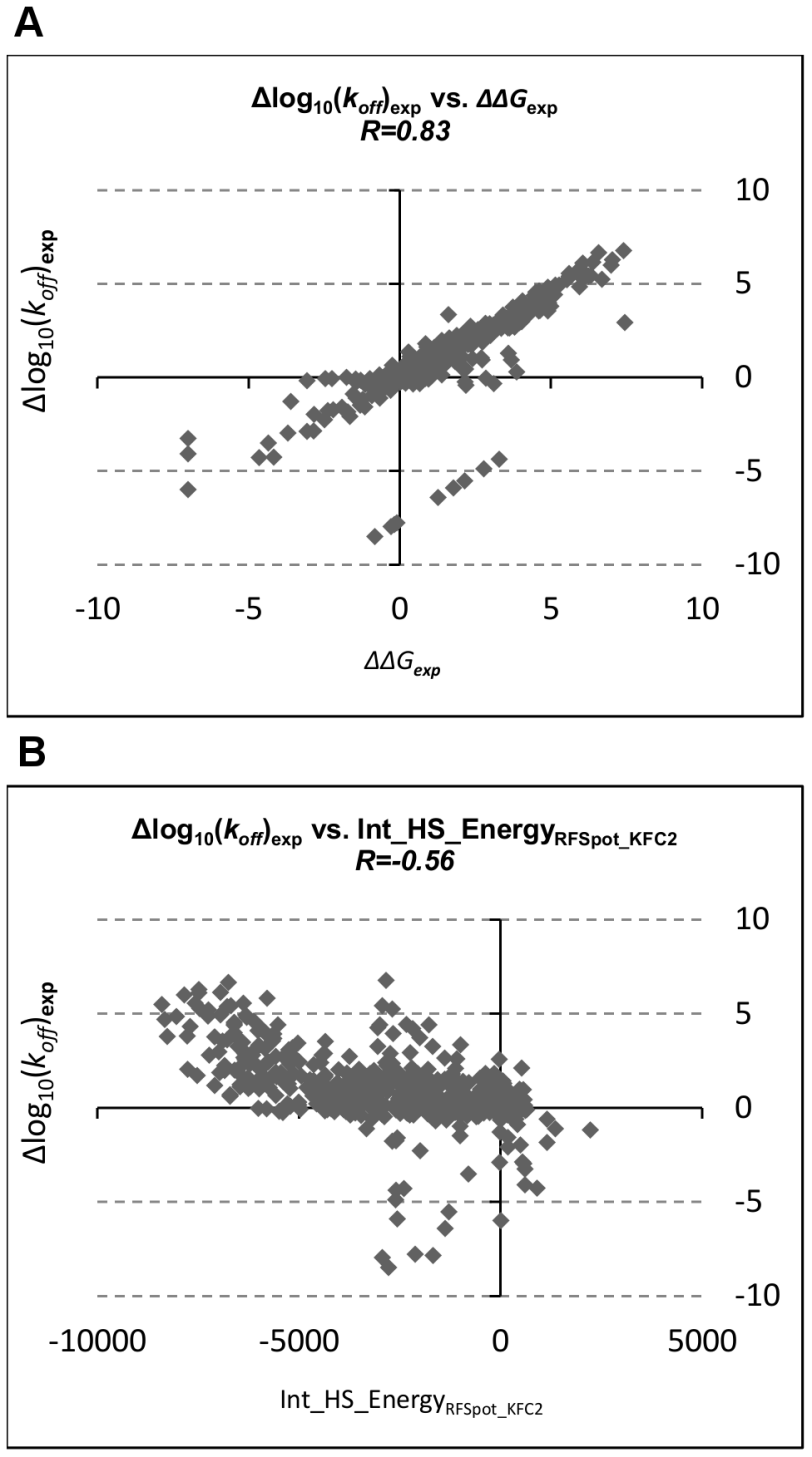
Relationship of off-rate changes upon mutation with change in binding free energy and change in interface hotspot energy. (A) The relationship between experimental values for Δlog_10_(*k_off_*) and *ΔΔG* for all the 713 mutations in the SKEMPI off-rate dataset. (B) The relationship between changes in interface hotspot energies, as predicted by *RFSpot_KFC2* hotspot predictor, and change in Δlog_10_(*k_off_*) for all the 713 mutations in the SKEMPI off-rate dataset. Note that 50% of off-rate mutants in this dataset involve mutations to non-alanine residues and include multi-point mutants. In turn *Int_HS_Energy* characterizes these changes with the use of single-point alanine *ΔΔG*s as highlighted in Figure 1.

Our novel approach of using the *ΔΔG*s of single-point alanine mutations to characterize off-rates is based on two generalizations. The first one being that, the change in binding free energy is mostly reflected through a change in the off-rate rather than the on-rate. If this is so, any prediction algorithm designed for *ΔΔG*s, may to some extent also be used for the prediction of Δ*k_off_* and vice-versa. For the 713 off-rate mutations used in this work, the corresponding experimental values for *ΔΔG* and Δlog_10_(*k_on_*) are also extracted (see Dataset S1), and the PCCs between them are shown in Table 3A. The correlations are calculated for single-point alanine mutations, single-point non-alanine, multi-point, and on all mutations. Namely, *ΔΔG*, shows a correlation of R = 0.83 with Δlog_10_(*k_off_*) (Scatter Plot in Figure 2A) and R = −0.6 with Δlog_10_(*k_on_*). More notably is that the *ΔΔG* of multi-point and non-alanine mutations is strongly reflected through a change in Δlog_10_(*k_off_*) (R = 0.96, R = 0.92 respectively). Other lines of evidence also show that the change in binding free energy is largely explained through a change in off-rate; Namely, mutagenesis studies in [44], [45] show that the increases in dissociation rate constants were the dominant cause for a decrease in binding affinity. Work on the related phenomenon of protein-DNA binding shows that 78% of the variance of log_2_(*k_off_*) is explained by the variance of information of the binding site sequence as opposed to 49% of the variance of log_2_(*k_on_*) [46]. In a somewhat similar line of reasoning, work on the enhancement of the protein-protein association rate shows that mutations that affect binding free energy, as a result of affecting the on-rate with no change in the off-rate, are found at surface-exposed sites and located at the vicinity of, but outside, the binding site - as those within the binding site are generally off-rate modulating [47]. With this in mind, for the 713 off-rate mutation dataset, only 25% of the mutants are located at the edges (Rim) or outside the binding site (Surface), hence we also expect that the larger portion of mutants in our data, to predominantly affect the off-rate, as is also confirmed by the correlations in Table 3A.

**Table 3:** Relationship between experimental *ΔΔG*, Δlog_10_(*k_off_*), Δlog_10_(*k_on_*) and change in interface hotspot energy (*Int_HS_Energy*) for 713 mutations in SKEMPI.

A	*ΔΔG*	Single-point alanine	Single-point non-alanine	Multi-point	All 713 mutations
	**Δlog_10_(** ***k_off_*** **)**	0.57	0.92	0.96	0.83
	**Δlog_10_(** ***k_on_*** **)**	−0.56	−0.65	−0.65	−0.60

B	*Int_HS_Energy*	Single-point alanine	Single-point non-alanine	Multi-point	All 713 mutations
	**Δlog_10_(** ***k_off_*** **)**	−0.33	−0.34	−0.62	−0.51
	**Δlog_10_(** ***k_on_*** **)**	0.12	0.08	0.22	0.17
	***ΔΔG***	−0.48	−0.29	−0.57	−0.53

(A) Shows PCC between experimental *ΔΔG* with the respective Δlog_10_(*k_off_*) and Δlog_10_(*k_on_*) for single-point alanine, single-point non-alanine, multi-point and all 713 mutations. (B) Shows PCC between *Int_HS_Energy* with the respective *ΔΔG*, Δlog_10_(*k_off_*) and Δlog_10_(*k_on_*) for single-point alanine, single-point non-alanine, multi-point and all 713 mutations. Experimental values for the 713 mutations used here are extracted from SKEMPI [41] and are presented in Dataset S1.

#### Explaining off-rate changes using ΔΔG energies from single-point alanine mutations

Given this link between *ΔΔG* and Δlog_10_(*k_off_*) we can use *ΔΔG*s as a starting point for the prediction of off-rates. The second generalization being, that we can do so using only hotspot energies from hotspot predictors. Hotspot predictors, although being *ΔΔG* predictors, are limited to characterizing *ΔΔG*s of single-point mutations to alanine, as the definition of a hotspot requires. Therefore, if one were to use the *ΔΔG*s of a hotspot predictor directly to estimate Δ*k_off_*, it is not able to predict the effects of multi-point mutations and non-alanine mutations (which form 49% of the 713 off-rate mutations in our dataset). The main motivation behind the hotspot descriptors designed in this work is therefore to be able to map the effects of multi-point mutations and non-alanine mutations into energies involving only single-point alanine mutations, where the latter can be predicted by off-the-shelf hotspot predictors. To assess this proposition, we make use of a representative hotspot descriptor *Int_HS_Energy*,
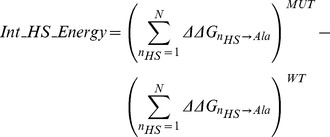
(1)where the effect of a mutation on the off-rate is calculated as the change in the sum of the hotspot energies across the interface pre- and post-mutation (See Figure 1). The PCC of *Int_HS_Energy* with Δlog_10_(*k_off_*), Δlog_10_(*k_on_*) and *ΔΔG* are shown in Table 3B. Given that *Int_HS_Energy* is generated separately using 6 hotspot prediction algorithms, all correlations presented are the average over its 6 instances. *Int_HS_Energy* provides a reasonable starting-point estimate of changes in both *ΔΔG* and Δlog_10_(*k_off_*), where the PCC of *Int_HS_Energy* with Δlog_10_(*k_off_*) (See Figure 2B) and *ΔΔG* are R = −0.51 and R = −0.53 respectively. The anti-correlation confirms that an increase in *wild-type* to mutant interface hotspot energies results in a lower off-rate and hence more stable complex.

**Figure 3:**
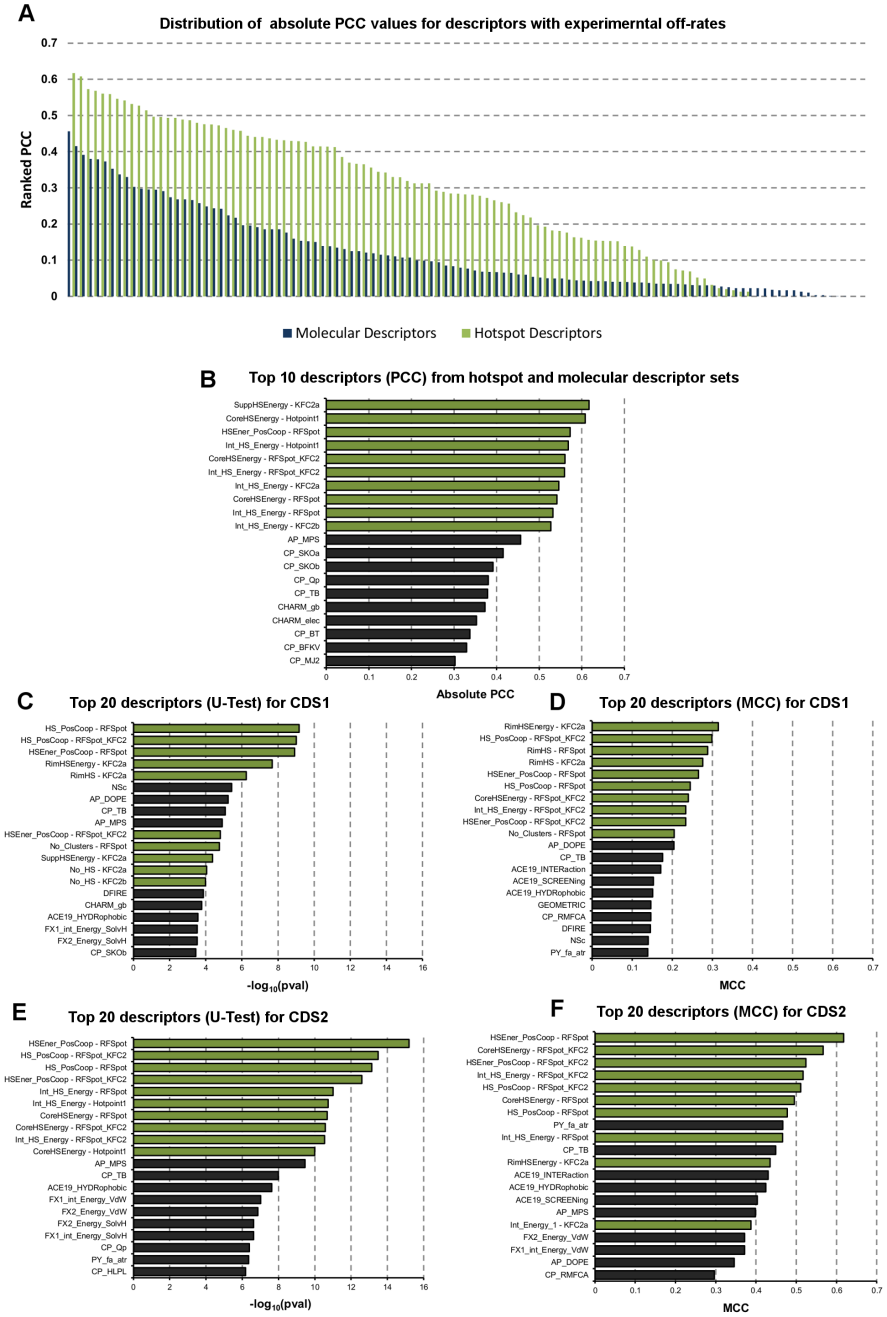
Hotspot and molecular descriptors for estimating change in off-rate. The hotspot descriptors designed in this work are benchmarked against a set of 110 molecular descriptors; both in their ability to estimate Δlog_10_(*k_off_*) and in their ability to detect stabilizing mutations of **Δlog_10_(** ***k_off_*** **)** <−1. The performance measures shown here enable us to assess the raw predictive power of the descriptors independent of any learning models. Green and black bars highlight descriptors from the hotspot and molecular descriptor sets respectively. (A) Comparison of the distribution of the absolute PCC values for the hotspot descriptors designed in this work against that for the molecular descriptors. The related list of descriptor names and their respective PCCs is found in Text S5. (B) Top 10 hotspot descriptors and top 10 molecular descriptor according to absolute PCC with experimental Δlog_10_(*k_off_*). (C) Mann Whitney U-Test rankings for all descriptors where values are ranked according to −log_10_(pval) and represent the discrimination ability of the descriptors for the detection of stabilizing mutants (**Δlog_10_(** ***k_off_*** **)** <−1) from neutral to destabilizing mutants (**Δlog_10_(** ***k_off_*** **)** >0) (Referred to as CDS1). This dataset contains 31 stabilizing mutants and 503 neutral to destabilizing mutants. (D) Matthew's Correlation Coefficient (MCC) rankings for all descriptors on same dataset. (E) and (F) are identical to (C) and (D) except that results are for off-rates that satisfy |**Δlog_10_(** ***k_off_*** **)|** >1. This dataset contains 31 stabilizing mutants and 213 destabilizing mutants (referred to as CDS2).

The strength of correlation at R = −0.51, and those achieved by other hotspot descriptors which also use single-point alanine *ΔΔG*s to describe off-rate changes, is better understood in the context of other descriptors for off-rate estimation. The absolute PCCs with Δlog_10_(*k_off_*) for both the molecular (110 in total, including physics-based energy terms and knowledge-based potentials) and hotspot descriptors (including 6 sets of 16 hotspot descriptors as generated using the 6 hotspot predictors) are calculated (See Text S5, Table S1). At n = 713, all absolute correlations of |R|>0.1 are highly significant with p<0.001. A ranked list for both sets is superimposed and shows that hotspot descriptors can explain changes in Δlog_10_(*k_off_*) with markedly higher correlations than a diverse set of molecular descriptors (see Figure 3A). One should also note that, this is only an assessment of the raw predictive power of the descriptors. Once such a hotspot descriptors are fed into machine learning models and trained on off-rate data, their predictive power can be combined synergistically with that of others to achieve correlations as high as R = 0.79 with Δlog_10_(*k_off_*), as is shown in subsequent sections.

#### Accounting for experimental conditions

It must be noted that, as with the *ΔΔG* values used to parameterize hotspot prediction algorithms, the *k_off_* values in the SKEMPI data set are taken from different sets of experiments and were thus measured in a range of experimental conditions. Therefore, we performed an assessment of how severely variations in experimental temperature, ionic strength and pH can introduce noise into log_10_(*k_off_*) and Δlog_10_(*k_off_*). Firstly, 635 of the 713 values come from experiments reported to be performed in the 295–298K range, and 72 values either did not have their temperature reported, or were reported as ‘room temperature’ or ‘standard conditions’, corresponding to the 293–298K range [41]. The remaining six experiments were performed at 323K. Thus, only 0.8% of the data lies outside of a 5K temperature range. Although not reported in the SKEMPI database, most of the rate constants were determined using surface plasmon resonance or stopped-flow fluorescence in a relatively narrow range of standard buffer conditions. Further, ionic strength and pH predominantly affect the rate of association rather than the rate of dissociation; electrostatic shielding and changes in protonation state influence the long-range forces which drive protein association, rather than the short-range forces which keep the buried surfaces of the binding partners together. For instance, in the M3-XCL1 complex, in which ionic strengths in the 0.2 to 1.5 M NaCl range were investigated, the rate of association varied by over 70-fold, while the rate of dissociation varied by less than 3 fold (Figure 2C and Table III of [48]). Similarly, in a study of a VEGF-antibody interaction, varying pH in the 6.5–8.5 range resulted in around 30% variation in dissociation rate, while varying the ionic strength in the 10–1000 mM range produced a two-fold change in *k_off_* [49]. Even assuming a large three-fold standard error in *k_off_*, this would result in a standard error of 3/ln10≈1.3 in log *k_off_* [49]. Lastly and most importantly, we make the assumption that though reference states may change across experimental methods and studies, within a given experiment the reference state is constant for the experimental determination of the *wild-type* and its mutants, which tend to be generated within the same experimental work. Given that we train on values for Δlog_10_(*k_off_*) = log_10_(*k_off_*)^Mut^ - log_10_(*k_off_*)^WT^, any systematic variations associated with experimental conditions are eliminated, and thus we believe that this issue is less prominent for mutation prediction as it is for *wild-type*. Given the above assessment, we believe that the noise introduced by merging data from the different experiments that make up the SKEMPI data set is significantly less than the variation in Δlog_10_(*k_off_*) values which we are investigating and span a range of 15.3.

#### Two-step complex dissociation

Complex association/dissociation of two proteins A and B can be described using a two-step reaction, where an encounter complex (AB*) is formed before the final complex (AB) [50], [51],
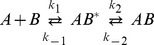
(2)


Both stable and unstable encounter complexes have been suggested [50] and rate limiting steps may vary depending on the complex in question [52]. In turn, experimental characterization of these encounter states remains a major challenge [50]. In the two-step model,
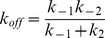
(3)k_−1_ represents the movement of proteins against an electrostatic field [51]. However, from experiments on a wide range of associating proteins, ionic concentration and hence long-range electrostatic forces have been shown to have a minimal effect on the off-rate [48], [49], [53], which suggests that in such cases, k_−1_ is not the rate-limiting step during complex dissociation. Here, k_−1_>>k_2_ and hence *k_off = _*k_−2_. Therefore, for such cases, the breakdown or stability of the final complex (AB) is the rate limiting step in complex dissociation [51]. Under this assumption, which is the one taken in this work, the encounter complex (AB*) need not be taken into consideration.

#### Hotspot and molecular descriptors and fine-grained detection of stabilizing mutations

**Figure 4:**
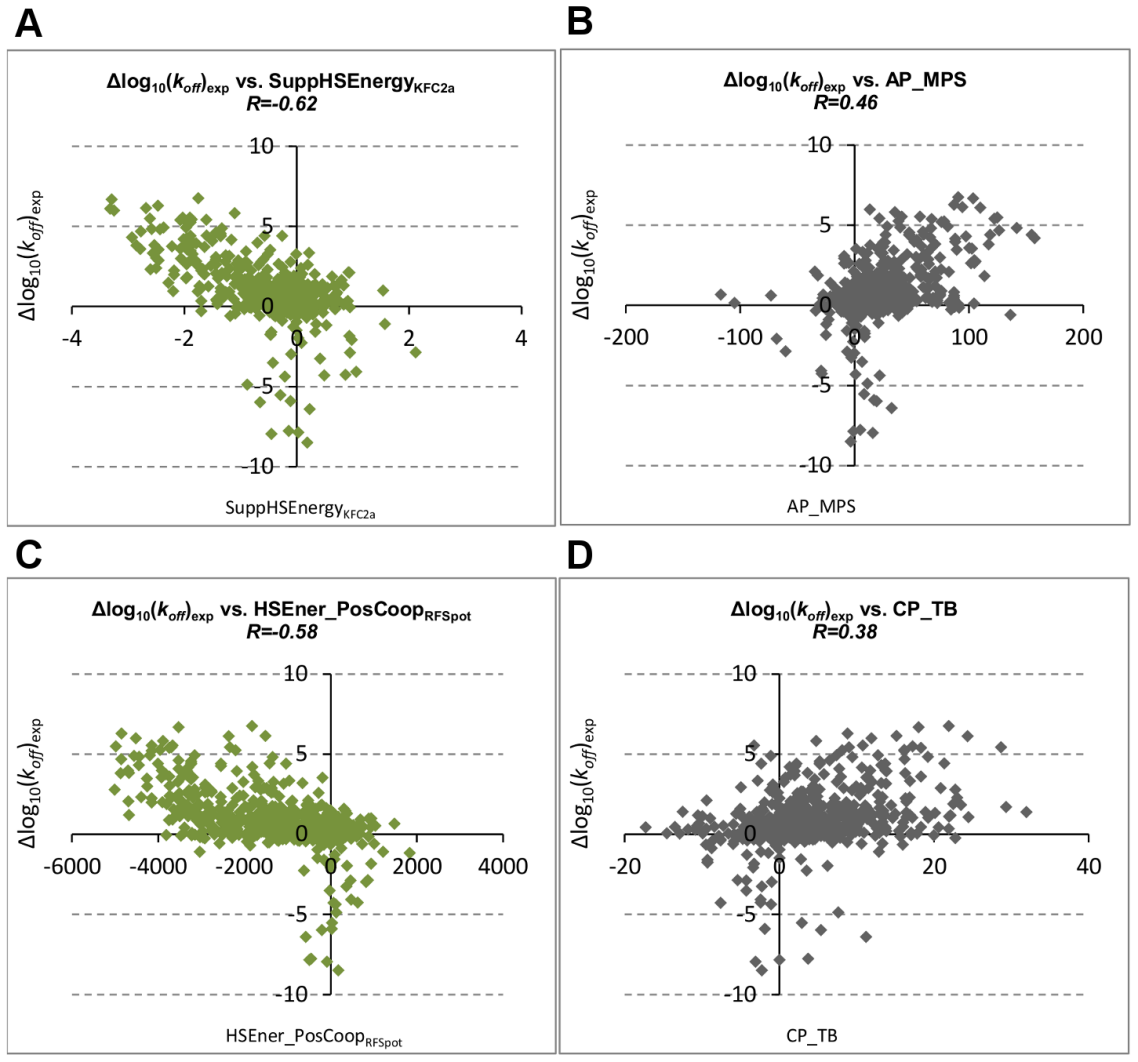
Hotspot and molecular descriptor scatter plots. The relationship between experimental values for Δlog_10_(*k_off_*) and (A) hotspot descriptors showing highest correlation with Δlog_10_(*k_off_*) (SuppHSEnergy_KFC2a_ - changes in hotspot energies in the support region as predicted by KFC2a [30]), (B) molecular descriptor showing highest correlation with Δlog_10_(*k_off_*) (AP_MPS - the DARS atomic potential [54]), (C) top performing hotspot descriptor for the detection of stabilizing mutants (HSEner_PosCoop_RFSpot_ – changes in hotspot energies on accounting for positive cooperativity in hotregions) and (D) top performing molecular descriptor for the detection of stabilizing mutants (CP_TB – coarse grained protein-protein docking potential).

The top 10 ranking descriptors according to PCC with Δlog_10_(*k_off_*) from both the hotspot and molecular descriptor sets are superimposed and are presented in Figure 3B. The highest ranked descriptors all relate to energetic changes in hotspots suggesting that changes in hotspot counts is not sufficient to characterize changes in off-rate. The most prominent being; the change in hotspot energies at the core region (*CoreHSEnergy*) and the change in the total hotspot energies at the interface (*Int_HS_Energy*). Given that these descriptors show up when generated using different hotspot prediction methods, indicates that they are insensitive to prediction biases of the hotspot predictors generating them. Further analysis on the sensitivity of the hotspot descriptors generated by each hotspot predictor is presented in Text S2. Other descriptors which show high PCCs with Δlog_10_(*k_off_*), include the change in total hotspot energy at the interface on accounting for positive hotregion cooperativity (*HSEner_PosCoop_RFSpot_* R = −0.57, see Figure 4C) and the change in hotspot energies in the support region (*SuppHSEnergy_KFC2a_* R = −0.62, see Figure 4A). Apart from the DARS atomic potential (AP_MPS [54], see Figure 4B) designed for protein-protein docking with |R| = 0.46, the top 10 molecular descriptors (Figure 3B (black)) are dominated by coarse-grain statistical potentials.

#### Hotspot and molecular descriptors and coarse-grained detection of stabilizing mutations

Understanding and predicting the consequences of mutations on protein-protein interactions is a precursor to two important endeavors in biology. At one end of the spectrum, destabilizing mutations at protein-protein interfaces are a hallmark of many cancers and other complex diseases [12]. At the other end, the identification of stabilizing mutations is central to computational protein design strategies [8]–[10], [40]. To assess the discriminatory power of the hotspot and molecular descriptors, the dataset is partitioned into (Δlog_10_(*k_off_*)<−1), representing the stabilizing portion of the dataset, and (Δlog_10_(*k_off_*)>0), representing the neutral to destabilizing portion of the dataset (referred to as CDS1 –Classification Dataset 1). Another dataset which removes the neutrals, as detailed in Material and Methods, is also used (referred to as CDS2). For an unbiased assessment of descriptor discrimination ability, a number of discrimination performance measures are calculated; the Mann Whitney U-Test (Figure 3C, E and Table S8 in Text S5), the Matthew's Correlation Coefficient (MCC) (Figure 3D, F), the Area Under the Curve (AUC) (Table S9 in Text S5) together with a number of classification performance measures (Table S4, Table S5) as described in Materials and Methods.

Similar to the correlations with Δlog_10_(*k_off_*) (Figure 3 A, B), the changes in hotspot descriptors show better discrimination abilities than changes in molecular descriptors (Figure 3C–F). This confirms that stabilizing mutations of multi-point and non-alanine nature, may be also be detected using simply the energies of single-point alanine mutations. Therefore, the more destabilizing are single-point mutations to alanine on the mutated interface, compared to the *wild-type* interface, the more stable is the interaction as a result of the mutation in question. Scatter plots of representative hotspot and molecular descriptors (*HSEnerPosCoop_RFspot_* and *CP_TB* [55], see Figure 4C, D) which do well on both CDS1 and CDS2 highlight a tendency to underestimate stabilizing mutations. For both CDS1 and CDS2, the positive cooperativity descriptors *HSEner_PosCoop*/*HS_PosCoop* dominate the ranked lists (Figure 3C–F) and RimHSEnergy/RimHS for CDS1 (Figure 3D). For example *RimHSEnergy_KFC2a_* achieves a TPR/FPR/MCC of (0.52/0.09/0.51) on CDS1 where neutrals are present. In turn, *HSEner_PosCoop_RFSpot_* achieves a TPR/FPR/MCC of 0.58/0.05/0.62 for the detection of stabilizing mutants on CDS2. Given that *HSEner_PosCoop_RFSpot_* supersedes *Int_HS_Energy* (additivity within hotregions assumption) and *HSEner_NegCoop* (negative cooperativity within hotregions assumption) suggests that applying the general assumption of positive cooperativity between hotspots within a hotregion, and accounting for it, provides higher detection rates of stabilizing mutations (*i.e.* Δlog_10_(*k_off_*)<−1). It should be noted however, that out of the three hotspot predictors which generate the most discriminatory hotspot descriptors (i.e. *RFSpot*, *RFSpot_KFC2* and *KFC2a)*, the positive cooperativity descriptors which show high discrimination abilities, are limited to those generated by *RFSpot* and *RFSpot_KFC2*. The relationship of Δlog_10_(*k_off_*) and cooperative effects within hotregions is addressed more specifically in the subsequent sections (see *Effects of hotregion size, count and cooperativity on the off-rate*).

### Off-rate prediction using machine learning models with hotspot and molecular descriptors

**Figure 5:**
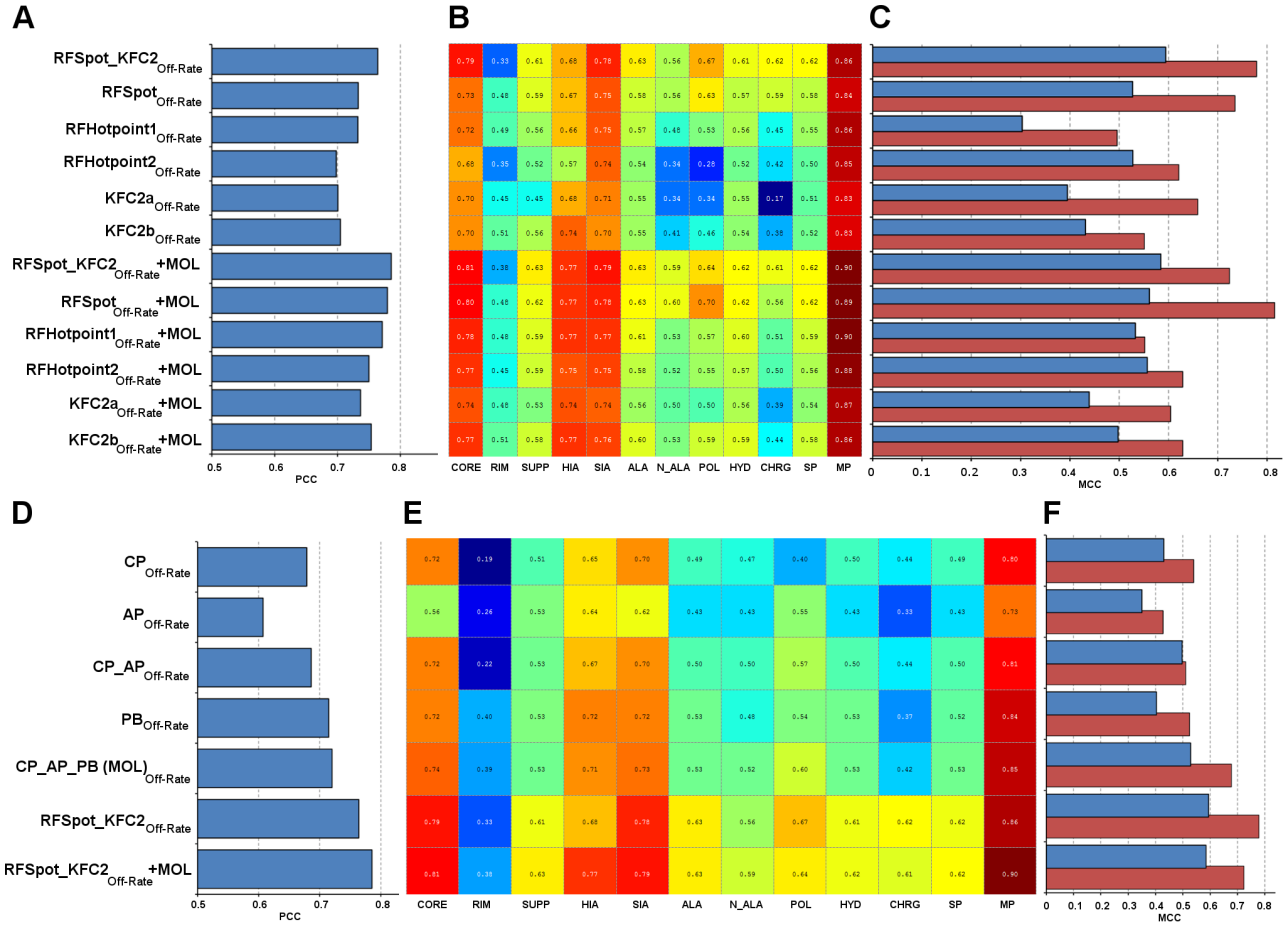
Off-rate prediction models using hotspot and molecular descriptors. A number of RF regression and classification models are built using different sets of hotspot and molecular descriptors. The prediction accuracy is also assessed on subsets of mutations defined as data regions. The data regions enable us to identify classes of mutations, which are consistently harder to characterize, data set biases and prediction patterns. (A) PCC values for off-rate model predictions with Δlog_10_(*k_off_*). Models use hotspot descriptors, or a combination of hotspot and molecular descriptors. The different methods indicate the hotspot prediction method by which the hotspot descriptors where generated from. (B) Data region analysis of predictions from each model. The prediction from each model are subset into the respective categories shown on the x-axis and values in matrix show PCC achieved by the given model for the given data region. (C) MCC values for off-rate classifier model predictions for classification data sets CDS1 in blue and CDS2 in red. CDS1 includes neutral mutations whereas CDS2 excludes neutral mutations; hence the detection of stabilizing mutants is enhanced in the latter, though results for CDS1 are more relevant for interface design scenarios. (D–F) are similar to (A–C) except that off-rate prediction models using subsets of molecular descriptors are investigated. CP – Coarse-Grain Potentials; AP – Atomic-Based Potentials; CP-AP – All Statistical Potentials; PB – Physics Based Energy Terms. As a benchmark comparison, results for *RFSpot_KFC2_Off-Rate_* (best performing off-rate predictor using hotspot descriptors) and *RF_Spot_KFC2_Off-Rate_+MOL* (best performing off-rate predictor using hotspot and molecular descriptors) are also included in (D–F).

**Figure 6:**
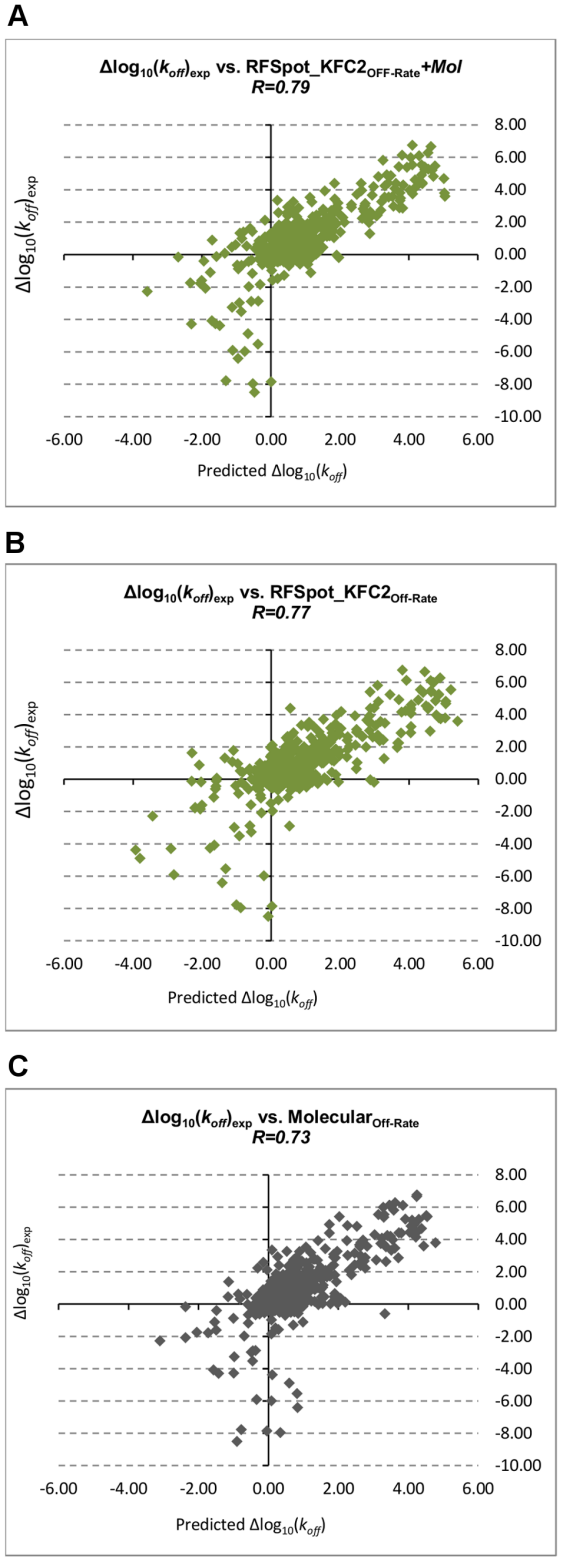
Off-rate prediction model scatter plots. The relationship between experimental values for Δlog_10_(*k_off_*) and predicted values for Δlog_10_(*k_off_*) with (A) *RFSpot_KFC2_Off-Rate_+MOL*, best performing off-rate prediction model combining hotspot and molecular descriptors. Hotspot descriptors for this model are generated using the *RFSpot_KFC2* hotspot prediction algorithm. (B) *RFSpot_KFC2_Off-Rate_+MOL*, best performing off-rate prediction model using only hotspot descriptors. Hotspot descriptors for this model are again generated using the *RFSpot_KFC2* hotspot prediction algorithm. (C) *Molecular_Off-Rate_*, off-rate prediction model using molecular descriptors. The addition of hotspot descriptors as observed in (A) to molecular descriptor model as shown in (B) notably improves the prediction of stabilizing mutants, which are all found in the lower left quadrant for *RFSpotKFC2_Off-Rate_+MOL*.

Confirming that energy estimates of single point-alanine mutations can be used to describe the effects of off-rate changes of single- and multi-point mutations not limited to alanine, we assess whether the whole set of 16 hotspot descriptors from each hotspot prediction algorithm can be combined synergistically in a model for off-rate prediction to achieve even higher correlations. A separate Random Forest (RF) regression model is trained on the 713 off-rate mutant dataset using the descriptors generated by each hotspot predictor (*RFSpot_Off-Rate_*, *RFSpot_KFC2_Off-Rate_*, *RFHotpoint1_Off-Rate_*, *RFHotpoint2_Off-Rate_*, *KFC2a_Off-Rate_* and *KFC2b_Off-Rate_*). In addition, models that add the set of 110 molecular descriptors to the hotspot descriptors (*RFSpot+Mol_Off-Rate_*, *RFSpot_KFC2+Mol_Off-Rate_*, *RFHotpoint1+Mol_Off-Rate_*, *RFHotpoint2+Mol_Off-Rate_*, *KFC2a+Mol_Off-Rate_* and *KFC2b+Mol_Off-Rate_*) are also built for comparison. Note that the ‘Off-Rate’ subscript is used to distinguish the off-rate predictor trained on hotspots, from the actual hotspot predictor generating the hotspot descriptors in question. The 20-Fold Cross-Validation (20-Fold CV) results are concatenated to form of a set of 713 test predictions and their PCC with Δlog_10_(*k_off_*) are shown in Figure 5A (See Table S1 for list of predictions for each model). The best performing off-rate predictor (*RFSpot_KFC2_Off-Rate_*, R = 0.79, see Figure 6A) combines the hotspot descriptors generated from *RFSpot_KFC2* hotspot predictor and the molecular feature set. In general, the models which combine both hotspot and molecular descriptors achieve higher correlations to the hotspot descriptor models, though which on their own, the latter still achieve correlations of R>0.7 using only 16 hotspot descriptors. Off-rate models using hotspot descriptors (Figure 6A and B), have more stabilizing mutations in the lower left quadrant, and hence such mutations tend to be less underestimated, than a model using molecular descriptors (Figure 6C).

#### Prediction patterns and data region analysis

To gain a better understanding of the stronger and weaker regions of correlation in the off-rate dataset, and how dependent this correlation is on the off-rate predictor in question, the predictions of each off-rate predictor are also assessed at different regions of the dataset referred to as data regions (see Materials and Methods) and presented in Figure 5B. All off-rate predictors obtain good correlation on core mutations, less so for support region mutations, and the weakest correlations are found on rim region mutations. The addition of molecular descriptors to the models, as presented in the lower half of the matrix, increases the accuracy of the predictors both at the core and support regions, though rim regions are still inadequately characterized. The hotspot descriptor predictors are better at capturing effects of mutants on Small-Interface-Area (SIA) than Large-Interface-Area (LIA) complexes. This discrepancy is alleviated with the addition of molecular descriptors to the models, where mutations in LIA complexes are characterized with similar correlations to SIA complexes. Single-point mutations to alanine are generally better characterized than single-point mutations to non-alanine, and the addition of molecular descriptors to the model again reduces this discrepancy. *RFHotpoint2_Off-Rate_*, *KFC2a_Off-Rate_* and *KFC2b_Off-Rate_* models show weak correlation for mutations to polar or charged residues, and better able to characterize mutations to hydrophobic residues. This discrepancy is not however observed in *RFSpot_Off-Rate_* and *RFSpot_KFC2_Off-Rate_* models. The low correlations for mutations to polar or charged residues for Hotpoint2*_Off-Rate_*, KFC2a*_Off-Rate_* and KFC2b*_Off-Rate_* are alleviated once the molecular descriptors are added to the models; and the highest correlations on these residue types are achieved by *RFSpot+Mol_Off-Rate_* and *RFSpot_KFC2+Mol_Off-Rate_*. In line with the PCCs shown in Table 3B, multi-point mutations are notably better characterized than single-point mutations, where in the former; correlations as high as R = 0.9 with Δlog_10_(*k_off_*) are achieved with certain models. This suggests that the subtleties of single-point mutations are harder to characterize than the collective effort of multi-point mutations. Note that, though theoretically, multi-point mutations have the potential to cause off-rate changes of larger magnitudes, this is not so in the present dataset, where the mean and standard deviation of|Δlog_10_(*k_off_*)| for multi-point mutations is 0.96 and 1.4 compared to 1.17 and 1.48 for SP mutations. Therefore, we cannot conclude that the reason for better prediction of multi-point mutations is related to being able to predict extreme changes in Δlog_10_(*k_off_*) better than subtle changes in Δlog_10_(*k_off_*). Results for more stringent forms of cross-validation and model predictions on data regions, which collect mutations on related complexes and interfaces together, are also presented in Text S3. Here it is observed that mutations on unseen complexes are markedly harder to predict, though on controlling for conformational changes, this difficulty is alleviated. Using specialized feature-selection models trained only on mutations from related complexes, and analyzing their descriptors shows that these are highly specific to certain classes of complexes. Therefore, such descriptors cannot generalize to unseen and unrelated complexes.

#### Prediction of stabilizing mutations

**Figure 7:**
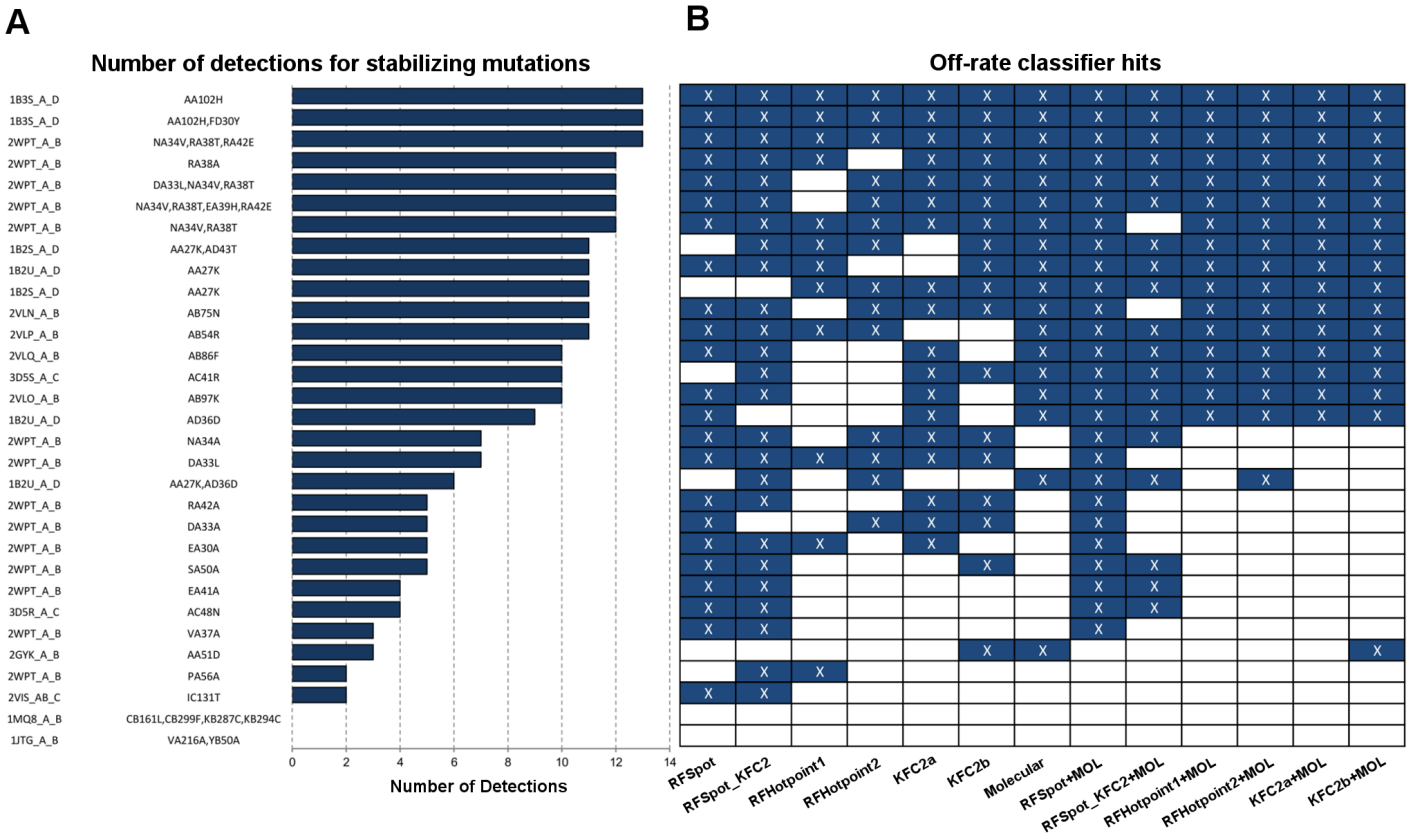
Detection of rare complex stabilizing mutations using off-rate classification models. (A) Ranked list of 31 stabilizing mutations (Δlog_10_(*k_off_*) <−1) in SKEMPI off-rate dataset. The list is ranked according to the number of off-rate prediction classification models that detect the mutation in question as stabilizing. Detections per model (B) are highlighted in white, and non-detections highlighted in black. The lower portion of (A) is dominated by single-point mutations to alanine residues, which suggests that the stabilizing effects of these mutations, as opposed to their more common neutralizing/destabilizing effects, are much harder to characterize.

Similar to the regression RF models, several RF classification models are also built for the detection of stabilizing (*i.e.* Δlog_10_(*k_off_*)<−1) mutants and results are presented for both Classifier Dataset 1 (CDS1) and Classifier Dataset 2 (CDS2). The MCC for the 20-Fold CV test predictions are presented in Figure 5C (Blue: CDS1, Red: CDS2) and related classifier performance measures are presented in Tables 4 A–B (See Table S2 and Table S3 for list of predictions for each model). As expected our ability to detect stabilizing mutants is diminished when neutral mutations are present. The highest MCC obtained for CDS1 is achieved by *RFSpot_KFC2_Off-RateC_* (MCC = 0.60, TPR = 0.45, FPR = 0.01) and *RFSpot+Mol_Off-RateC_* for CDS2 (MCC = 0.82, TPR = 0.84, FPR = 0.02). Figure 7A shows the list of 31 stabilizing mutants (Δlog_10_(*k_off_*)<−1) sorted according to the number of classifiers which detect the given mutation as stabilizing. Of particular interest are those stabilizing mutations which go undetected and therefore only data from CDS2 is used, as all mutations undetected in CDS2 were also undetected in CDS1, though not the contrary. Two stabilizing mutants go undetected by all the predictors, namely the double alanine mutant VA216A-YB50A on 1JTG and the 4-point mutant CB161L-CB299F-KB287C-KB294C on 1MQ8. Stabilizing mutations which are the harder to predict, as shown by the inability of a number of different off-rate classifiers to detect them (Figure 7B), generally involve a mutation to an alanine residue. Complex stabilizing alanine mutations have been previously reported [56], [57], and the likely interpretation is that several side-chains may sometimes hinder binding. For example, several alanine-shaving experiments have show an increase in binding affinity between protein binding partners, as found for an octa-alanine mutant of the hGH receptor which binds its hGHbp ligand 50-fold times tighter than the wild-type [56], [57].

**Table 4:** Performance of off-rate classification models for the detection of stabilizing mutations.

A								
Descriptor type	Off-rate prediction model	TPR	FPR	MCC	F1	ACC	Spec	Prec
Hotspot+Molecular	RFSpot+MOL	0.839	0.023	0.815	0.839	0.959	0.977	0.839
Hotspot	RFSpot_KFC2	0.806	0.028	0.778	0.806	0.951	0.972	0.806
Hotspot	RFSpot	0.742	0.028	0.735	0.767	0.943	0.972	0.793
Hotspot+Molecular	RFSpot_KFC2+MOL	0.677	0.019	0.723	0.750	0.943	0.981	0.840
Hotspot	KFC2a	0.613	0.023	0.659	0.691	0.931	0.977	0.792
Molecular	MOL	0.581	0.019	0.653	0.679	0.931	0.981	0.818
Hotspot+Molecular	Hotpoint2+MOL	0.548	0.019	0.629	0.654	0.927	0.981	0.810
Hotspot+Molecular	KFC2b+MOL	0.548	0.019	0.629	0.654	0.927	0.981	0.810
Hotspot	Hotpoint2	0.484	0.009	0.621	0.625	0.927	0.991	0.882
Hotspot+Molecular	KFC2a+MOL	0.516	0.019	0.604	0.627	0.922	0.981	0.800
Hotspot+Molecular	Hotpoint1+MOL	0.452	0.019	0.552	0.571	0.914	0.981	0.778
Hotspot	KFC2b	0.516	0.033	0.551	0.593	0.910	0.967	0.696
Molecular	CP	0.355	0.005	0.539	0.512	0.914	0.995	0.917
Molecular	PB	0.419	0.019	0.524	0.542	0.910	0.981	0.765
Molecular	CP_AP	0.323	0.005	0.510	0.476	0.910	0.995	0.909
Hotspot	Hotpoint1	0.387	0.019	0.496	0.511	0.906	0.981	0.750
Molecular	AP	0.355	0.028	0.428	0.458	0.894	0.972	0.647

B								
Descriptor type	Off-rate prediction model	TPR	FPR	MCC	F1	ACC	Spec	Prec
Hotspot	RFSpot_KFC2	0.452	0.006	0.594	0.583	0.964	0.994	0.824
Hotspot+Molecular	RFSpot_KFC2+MOL	0.355	0.000	0.585	0.524	0.964	1.000	1.000
Hotspot+Molecular	RFSpot+MOL	0.387	0.004	0.561	0.533	0.962	0.996	0.857
Hotspot+Molecular	Hotpoint2+MOL	0.355	0.002	0.557	0.512	0.962	0.998	0.917
Hotspot+Molecular	Hotpoint1+MOL	0.355	0.004	0.533	0.500	0.960	0.996	0.846
Hotspot	Hotpoint2	0.290	0.000	0.528	0.450	0.960	1.000	1.000
Hotspot	RFSpot	0.452	0.013	0.527	0.538	0.956	0.987	0.667
Molecular	MOL	0.323	0.004	0.503	0.465	0.958	0.996	0.833
Hotspot+Molecular	KFC2b+MOL	0.290	0.002	0.498	0.439	0.958	0.998	0.900
Molecular	CP_AP	0.258	0.000	0.497	0.410	0.958	1.000	1.000
Hotspot+Molecular	KFC2a+MOL	0.258	0.004	0.439	0.390	0.955	0.996	0.800
Hotspot	KFC2b	0.226	0.002	0.431	0.359	0.955	0.998	0.875
Molecular	CP	0.194	0.000	0.430	0.324	0.955	1.000	1.000
Molecular	PB	0.226	0.004	0.403	0.350	0.953	0.996	0.778
Hotspot	KFC2a	0.290	0.012	0.395	0.391	0.949	0.988	0.600
Molecular	AP	0.129	0.000	0.350	0.229	0.951	1.000	1.000
Hotspot	Hotpoint1	0.097	0.000	0.303	0.176	0.949	1.000	1.000

(A) Shows classification accuracy measures for the for the detection of stabilizing mutations (Δlog_10_(*k_off_*) <−1) from destabilizing mutations (Δlog_10_(*k_off_*) >1). This dataset contains 31 stabilizing mutants and 213 destabilizing mutants (referred to as CDS2). (B) Shows results for the detection of stabilizing mutations (Δlog_10_(*k_off_*) <−1) from neutral to destabilizing mutations (Δlog_10_(*k_off_*) >0). This dataset contains 31 stabilizing mutants and 503 neutral to destabilizing mutants (referred to as CDS1).

#### Off-rate prediction using molecular descriptors

The off-rate prediction models investigated so far concentrated on the use different off-rate prediction models which use hotspot descriptors generated from different hotspot predictor algorithms. Here the performance of models created from different categories of molecular descriptors is shown. These include Atomic Potentials (AP), Coarse-grain Potentials (CP) and Physics-Based energy terms (PB) (See Text S1). The same protocol as in the previous sections is followed, where regression models are trained on the 713 mutant dataset and 20-Fold CV results are analyzed as a whole and also separately on different regions of the data set. Classification models are also built on CDS1 and CDS2. As a benchmark comparison, the results of the best performing off-rate predictors built on hotspot descriptors (*RFSpot_KFC2_Off-Rate_*) and the best off-rate predictor built on both hotspot and molecular descriptors (*RFSpot_KFC2+Mol_Off-Rate_*) are also presented with those of the molecular descriptor models (See Figure 5D–F). The physics-based descriptors' model (PB*_Off-Rate_*, R = 0.72) which includes CHARMM [58], FoldX [43] and PyRosetta [59] energy terms performs better than the models with coarse-grain (CP*_Off-Rate_*, R = 0.68) and atomic (AP*_Off-Rate_*, R = 0.61) statistical potentials alone or combined (CP_AP*_Off-Rate_*, R = 0.69). *RFSpot_KFC2_Off-Rate_* (R = 0.76) built on hotspot descriptors only, achieves higher PCC than a model with all molecular descriptors combined (CP_AP_PB*_Off-Rate_*, R = 0.72), whereas the highest correlation is achieved when combining both molecular and hotspot descriptors (*RFSpot_KFC2+Mol_Off-Rate_*, R = 0.79) as already highlighted in the previous section. On analysis of the various regions of the off-rate dataset (Figure 5E) we observe that on all data regions, either the hotspot descriptor model or the molecular and hotspot descriptor models combined always perform better than the molecular descriptor models. This is most notable for SIA complexes and charged residues. Again mutations at the rim regions are the least accurately predicted, and multi-point mutants are better characterized than singe-point mutants. This is consistent with what is observed in Figure 5B for the hotspot descriptor models. The highest discriminatory power for the detection of stabilizing mutants, for both CDS1 and CDS2, is achieved by the off-rate models which make use of hotspot descriptors (Figure 5F).

### Specialized feature selection models for off-rate prediction

**Figure 8:**
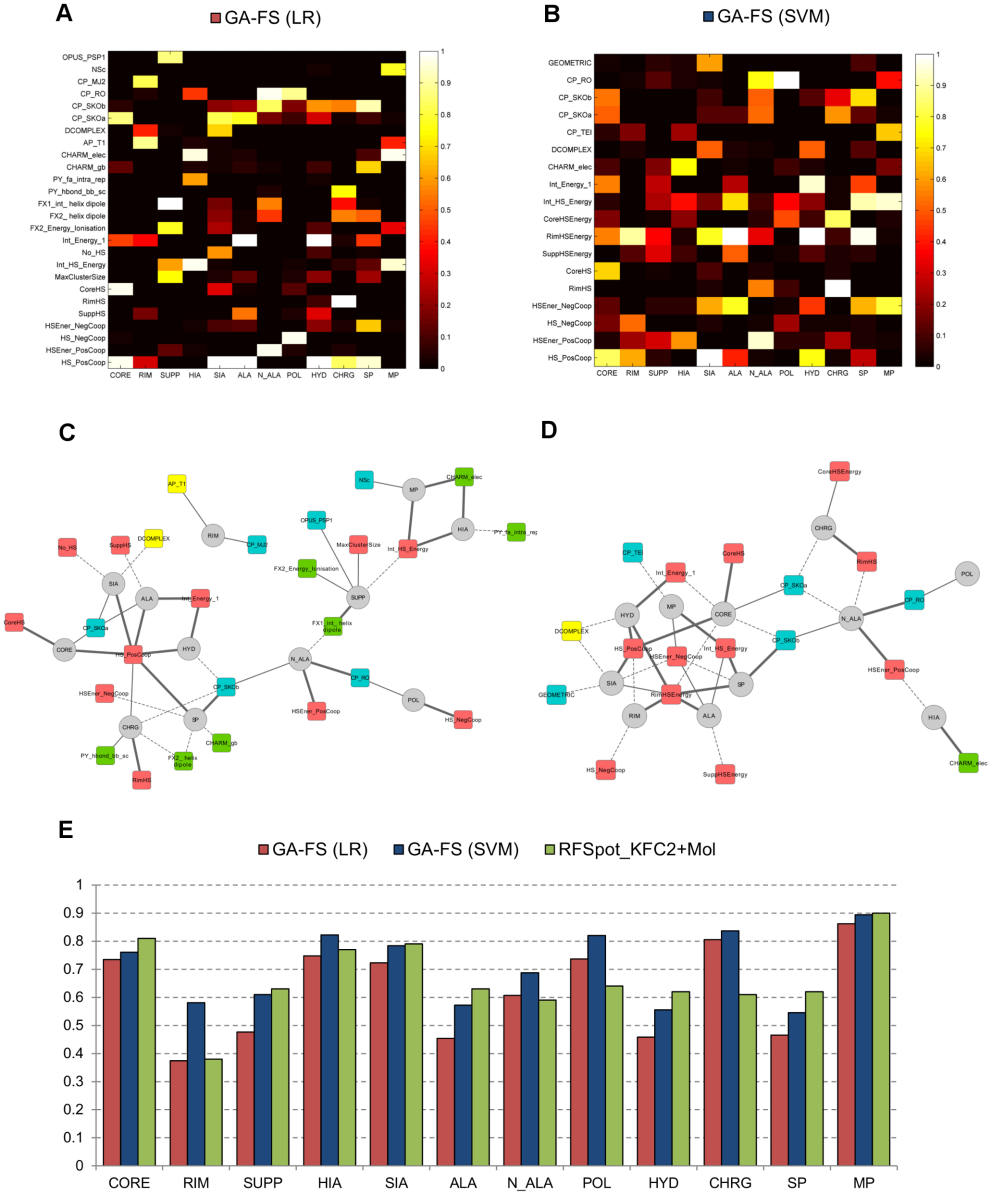
Specialized feature selection models and descriptor-data region networks. Feature selection models using a genetic algorithm are run for different data regions of the off-rate dataset for which both linear (using Linear Regression) and non-linear (using SVM regression) models are investigated. For each data region, the GA-FS is run 50 times designed to find an optimal feature set of size 5. Initial features available in the population are the 110 molecular descriptors and 16 hotspot descriptors generated by *RFspot_KFC2*. An inner-cross validation loop is used as a scoring function for driving the feature selection whereas and outer-cross validation loop is used to assess the model prediction accuracy. (A) and (B) shows the importance of the most selected features for each data region. The features shown are those that are part of the final model for any data region on more than 50% of the GA-FS runs, and the color bar displays this percentage. The features on the y-axis are ordered as: coarse-grain potentials, atomic-based potentials, physics-based energy terms and hotspot descriptors. (C) and (D) are descriptor-data region networks for (A) and (B) respectively. Circled nodes represent data regions and square nodes represent features; therefore, only edges between circle and square nodes are present. An edge is present if the feature is in the final model for the given data region in more than 50% of the GA-FS runs (dotted edge), between 70–90% of the GA-FS runs (normal edge), more than 90% of the GA-FS runs (bold edge). Coarse-grain potentials (blue), atomic-based potentials (yellow), physics-based energy terms (green), hotspot descriptors (pink) and data regions (gray). From the descriptor-data region networks, descriptors highly specific to certain classes of off-rate mutations can be observed. Conversely, as in the case of the GS-FS (SVM) data region network, a cluster of broadly-predictive hotspot descriptors is also shown. (E) Mean PCC of the optimal models found by the GA-FS runs for each data region. For comparison, PCC results on the data regions results are also shown for *RFSpot_KFC2_Off-Rate_+Mol*. Note that the latter model is trained on all 713 off-rate mutations, and the predictions are separated post prediction into data regions and analyzed for their PCC. This effectively compares the predictions of specialized models vs. one-fits-all model. Though we find no evidence that specialized models perform better than a one-fits-all model, certain subsets of mutations, such as those at the rim regions, show notable improvements when a specialized model is employed.

Previous analysis has been performed using models trained on all the 713 off-rate mutations in the dataset, of which the predictions were then subdivided into data regions for separate analysis. Here, descriptors, which are specific to the prediction of mutations within each data region, are investigated. To do so, separate models are built for the different data regions of the dataset using a Genetic Algorithm for Feature Selection (GA-FS) as described in Materials and Methods. All 110 molecular descriptors and 16 hotspot descriptors generated from the *RFSpot_KFC2* hotspot predictor are available for feature selection. The feature set size is set to 5 features to avoid over-fitting and both non-linear (using Support Vector Machines, SVM) and linear (using Linear Regression, LR) models are investigated. For every data region, 50 separate GA-FS runs are performed; an inner-cross validation loop is used for FS (And SVM parameter optimization), whereas an outer-cross validation loop is used for testing the final model, of which the results are summarized in Figure 8E (blue and red). The GA-FS models built on rim and support region mutations achieve markedly lower correlations than core region models, though a non-linear model increases the accuracy of the latter two models. There are no notable differences in the ability to model LIA and SIA complexes; however, multi-point mutations are markedly better predicted than single-point mutations. Polar and charged mutations show good correlation which decreases when considering hydrophobic residues.

#### Broadly predictive and highly specific descriptors for off-rate data regions

To uncover links between descriptors and certain classes of mutations, the descriptors important for the prediction of mutations within each data region are also analyzed. For each region, the descriptors which are part of the final model in at least half of the total number of runs are singled out for analysis and presented in heat maps which indicate their importance to the given data region (Figure 8A: GS-FS (LR) and Figure 8B GS-FS (SVM)). On the y-axis, the singled out descriptors are listed and categorized according to descriptor type (CP, AP, PB, and Hotspot Descriptors from top to bottom), and each data region shown on the x-axis. Globally, it is observed that whereas for LR models, top features are distributed throughout the four main feature categories, for the non-linear SVM models, 61% of the features are hotspot descriptors, suggesting that non-linear relationships between hotspot descriptors can be better exploited for the predictions of off-rates. To visualize the interconnections between descriptors and data regions, descriptor-data region networks are generated for both the LR (Figure 8C) and SVM (Figure 8D) GA-FS runs. An edge between a descriptor and a data region is shown if the given descriptor is part of the final GA-FS model in at least 50% of the GA-FS runs for the given data region (with increasing edge weight for >50%). For the LR model, two statistical potentials (*AP_T1* [55] , *CP_MJ2* [60]) are highly specific to rim region mutations, whereas others such as *HS_PosCoop*, as highlighted by its high degree, are broader in their predictive value and can explain off-rate changes in a number of data regions collectively. Interestingly, for the support region, *MaxClusterSize* is invoked which suggests that larger hotregions in the support regions may be important for complex stabilization. Whereas certain descriptor-data region relationships hold for both LR and SVM models, such as electrostatic contribution (*CHARMM_elec* [58]) for mutations on complexes of LIA, the ability to model non-linearities between features, invokes some different descriptors. Most notably, a key observation specific to the SVM descriptor-data region network, is a central cluster of highly interconnected hotspot descriptors and data regions, which involve *HS_PosCoop*, *HSEner_PosCoop*, *Int_HS_Energy* and *RimHSEnergy*.

Having looked at both the use of specialized models (GS-FS SVM/LR) for different types of complexes, mutations, and regions on the interface, and the use of a global one-fits-all models (such as *RFSpot_KFC2+Mol_Off-Rate_*) for off-rate prediction, it is important to highlight whether there is any advantage in having such specialized models for the prediction of off-rate mutations which fall under a given data region. In Figure 8E, the correlations for the various data regions are compared. For most of the regions, having one-fits-all model suffices, however, for mutations in the rim region, and mutations to charged or polar residues, having a specialized model markedly increases our ability to characterize off-rates in these data regions.

### Central and distributed stability regions in protein-protein complexes

One advantage of using hotspot descriptors to estimate off-rates is the ability to localize interface regions of high stability and assess how mutations affect the distribution of stabilities, within these regions. The importance of the core interface region is implicated largely due to the tendency of hotspots to preferentially occur in this region [24]. On the other hand rim residues seem to play a more secondary role of solvent shielders by providing an ideal dielectric constant for better interactions at the core [24]. In this section we analyze hotspot energies at specific regions of the interface, namely the core, rim and support regions and evaluate whether complex stability can be effectively disrupted homogenously across the interface or preferentially in a particular region. More specifically the role of rim residues is re-investigated in the light of off-rate changes upon mutations on complexes of various sizes and interface-areas.

*CoreHSEnergy*, *RimHSEnergy* and *SuppHSEnergy* represent the change in total hotspot energies limited to each region upon mutation. Effectively, the PCC of these descriptors with the off-rate expresses how well changes in the given region show themselves as changes in log_10_(*k_off_*) - irrespective of changes in hotspot energies in any other region. Therefore, by assessing the relative PCCs of the three regions we can gauge whether a given region acts independently and dominates in its contribution to complex stability compared to other regions. Given that we have 6 instances of each hotspot descriptor, as generated per each hotspot predictor, the correlations for each descriptor shown are the mean of each descriptor's correlation under the 6 hotspot predictors. Hence results can be considered to be independent of the hotspot predictor generating the hotspot descriptors. From the PCCs of the three hotspot region specific descriptors (*CoreHSEnergy* |R| = 0.48, *RimHSEnergy* |R| = 0.20 and *SuppHSEnergy* |R| = 0.38), it is observed that changes in the hotspot energies at the core affect the off-rate more significantly than the rim (p<<0.01) and support region (p<0.01). Given that 355 mutations affect hotspot energies in the core region compared to 148 and 182 for rim and support regions respectively, results may however be biased. For example, if fewer events are observed at the rim region, there is less chance of the rim region playing a significant role in off-rate changes, when looking at it globally over a population of complexes as is done presently. To remove this potential bias, the subset of mutations, which affect all three regions simultaneously, is extracted and the PCC recalculated. The PCCs still suggest dominance from the core region (|R| = 0.53), more significantly than the rim region (|R| = 0.22 p<<0.01).

#### Stability regions in SIA and LIA complexes

**Figure 9:**
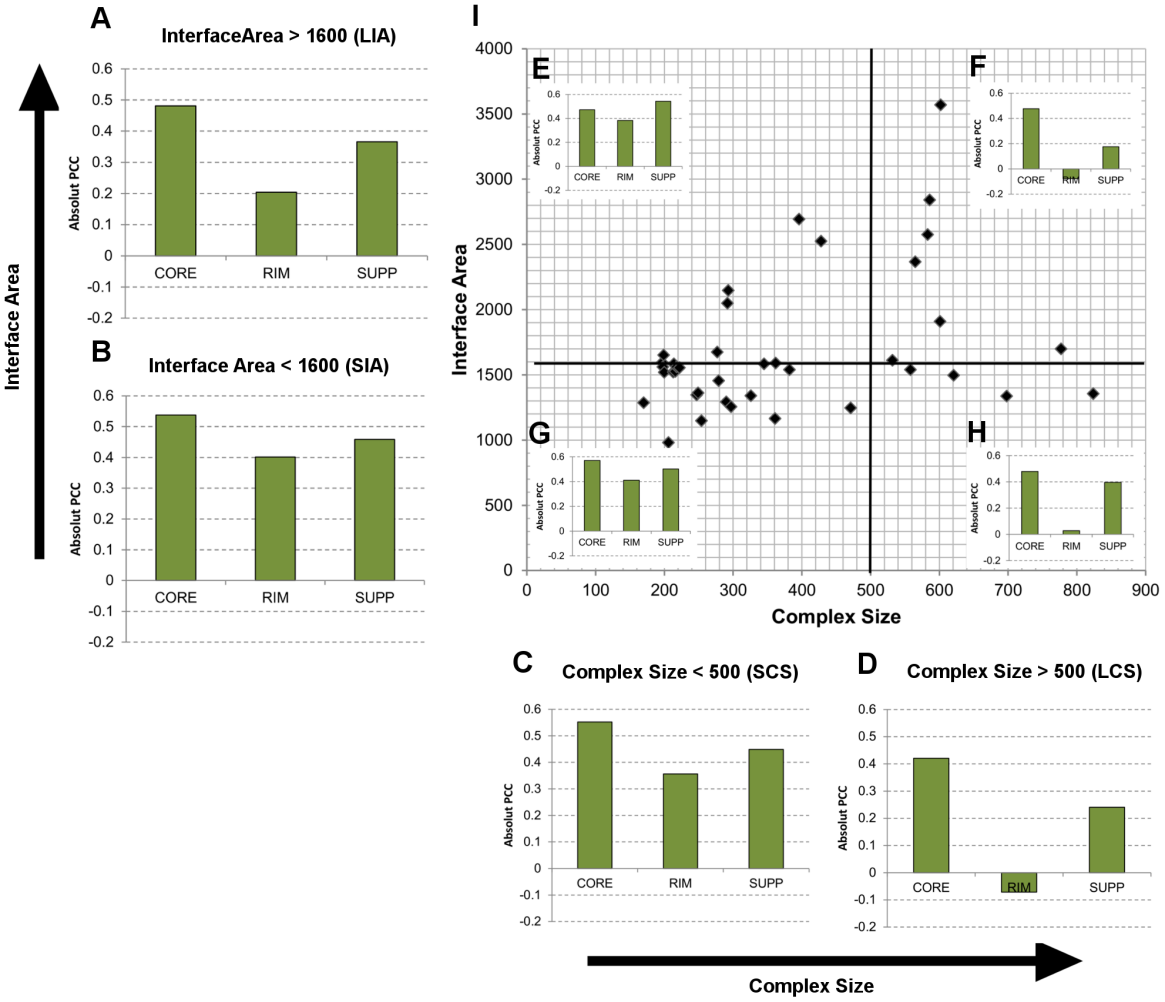
Stability regions, interface-area and complex-size. The changes in hotspot energies upon mutation are assessed at three interface regions, which enable us to explore changes in the distribution of stability for complexes of different size and interface-area. CORE, RIM and SUPP represent the PCCs of CoreHSEnergy/RimHSEnergy/SuppHSEnergy averaged for the 6 hotspot prediction algorithms with Δlog_10_(*k_off_*).(A) PCCs for mutants on Complexes with interface-area >1600 Å^2^ (LIA). (B) PCCs for mutants on complexes with interface-area <1600 Å^2^ (SIA). (C) PCCs for mutants on complexes with size <500 residues (SCS). (D) PCCs for mutants on complexes with size >500 residues (LCS). (E) LIA-SCS, (F) LIA-LCS, (G) SIA-SCS, (H) SIA-LCS. (I) Scatter plot of complex size vs. interface area for all complexes in off-rate mutant dataset. Here it is observed that complex stability is distributed across all three regions for small-size complexes (C, E and G), whereas the core becomes a localized region of stability for large-complex sizes (D, F, H). On analysis of the interface-area vs. complex-size subsets (E–H), the distribution of stability regions is affected primarily through complex-size irrespective of interface-area.

To investigate whether the relative importance of these three regions of stability change when considering complexes of different interface areas, the dataset is divided into Small-Interface-Area (SIA) complexes (<1600 Å^2^ buried surface area) and Large-Interface-Area (LIA) complexes (>1600 Å^2^ buried surface area). The threshold of 1600 Å^2^ is such that both subsets are of similar number of examples. The mean PCC for the *CoreHSEnergy*, *SuppHSEnergy* and *RimHSEnergy* for LIA and SIA complexes is calculated and shown in Figures 9A and 9B respectively. For the LIA complexes, a dominant contribution from the changes in core hotspot energies (*CoreHSEnergy* |R| = 0.48) and minimal contribution from *SuppHSEnergy* (|R| = 0.37) and *RimHSEnergy* (|R| = 0.20) is observed. Therefore, even though a given set of mutations might be affecting support or rim regions, it is the changes in hotspot energies at the core region which show up as the dominant changes in the off-rate (|R| = 0.48). For SIA complexes, changes in hotspot energies in both the in the rim regions show a highly significant 2-fold increase in correlation (p<<0.01). This renders all three regions with similar contributions to complex stability (*CoreHSEnergy* |R| = 0.56, *SuppHSEnergy* |R| = 0.46, *RimHSEnergy* |R| = 0.40). For LIA complexes, the ratio of mutations applied in positions that affect the core to those that affect the rim is 2∶1. On considering SIA complexes this ratio increases to 3∶1. Therefore, we negate the possibility that the increased presence of the rim hotspot energies from LIA to SIA is due to an increase in the number of mutations affecting these regions. Rather, we see an increase in correlation of *RimHSEnergy* in spite of a reduction in mutations affecting these regions. As an additional test which accounts for biases in the number of examples affecting each region, the correlations are calculated for only the mutations which make changes in the respective region, again taking an average over all 6 hotspot predictors' descriptors. Here no significant changes in correlation are observed in LIA and SIA complexes for the core and support region. For LIA complexes, changes in rim hotspot energies have minimal effect on the off-rate with |R| = 0.29, whereas for SIA complexes, a 1.75-fold increase (p<0.01) in correlation is observed (|R| = 0.51).

#### Complex size, interface area and stability regions

To probe further the difference in the distribution of stability regions in SIA and LIA complexes, the size of the complexes is also taken into consideration. The dataset is divided into the mutations which are found on Large-Complex-Size (LCS) (with 231 mutations) and Small-Complex-Size (SCS) complexes (with 482 mutations). The PCC for *CoreHSEnergy*, *RimHSEnergy*, *SuppHSEnergy* averaged over the descriptors from all hotspot predictors is calculated for both LCS and SCS (Figure 9C, D). Core hotspots are critical to the stability of LCS complexes whereas for SCS complexes, all three regions are important. This effect is synonymous with what is observed in LIA and SIA complexes, though the increase in correlation for *RimHSEnergy* (R = 0.07 to R = −0.36 p<<0.001) is more pronounced for complex size. Noting that fewer mutations are on LCS complexes, the percentage of mutants affecting each region in LCS, compared to that for SCS, is similar across the three regions (61%, 52% and 46% for core, rim and support regions respectively) and therefore shows no relationship to the changes seen in the PCC of the three regions from LCS to SCS. On the 50 complexes considered in the 713 off-rate mutant dataset, complex size and interface size show a correlation of R = 0.55 (Scatter plot in Figure 9I). The correlation is higher (R = 0.74) for complexes sizes of less than 500 residues, and becomes insignificant (R = 0.18) beyond complex sizes of 500 residues. The dataset is therefore further divided into four regions (Scatter Plot I), which include: SIA-SCS (191 mutations) , SIA-LCS (67 mutations), LIA-SCS (191 mutations), LIA-LCS (164 mutations) and again the PCC for *CoreHSEnergy*, *RimHSEnergy*, *SuppHSEnergy* averaged over the descriptors of all hotspot predicators is calculated and shown in Figure 9G: SIA-SCS, Figure 9H: SIA-LCS, Figure 9E: LIA-SCS and Figure 9F: LIA-LCS. Here it is observed that given a fixed complex size (SCS or LCS), moving from small interface areas to larger interface areas, the landscape for the contributions of the core, rim and support regions is unchanging. Therefore, independent of the interface area size, for low complex sizes the off-rate has the propensity to be affected equally from all regions of the interface, whereas for high-complex sizes, stability is primarily emanating from core hotspots. Further analysis of SCS and LCS complexes shows a greater sensitivity in off-rate changes upon mutations for SCS complexes; the mean |Δlog_10_(*k_off_*)| is 1.4 and 0.69 for SCS and LCS complexes respectively. Though the latter result is intuitive, in that changes on large complexes are less likely to have effects as significant as those on small complexes, the key finding here is that on dissection of the three interface regions, the reduction in the ability to make significant changes in LCS is not equally shared on the three regions. Rather, mutations at the core can still have notable effects on the stability of large complexes as in the case of smaller complexes.

From our findings we confirm that the higher sensitivity of SCS complexes to mutations manifests as an increase in the role of the rim regions and also possibly the support regions. Support regions represent residues which are generally buried both in the unbound and bound structures; therefore, hotspots in this region are primarily responsible for monomer stability as their disruption is likely to affect intra-protein contacts. Given the correlations observed between protein-size and protein stability [61], it is likely that the possible increased role of support regions in SCS complexes, though not as evident here as is for rim regions, is related to monomer size. Rim region residues on the other hand are generally exposed both in the unbound and bound states, but form inter-protein contacts in the complex state. Of particular interest is the observation that rim hotspots are unimportant for the stability of large complexes, even for small-interface-areas. However, given the small number of mutations affecting these complexes (67 mutations), to substantiate this observation, further experimental data may be required. Also for additional validation, analysis of the flexibility of rim regions and the contribution such flexibility is likely to make to the dissociation process, using for example MD simulations on large and small complexes with small-interface-areas, may give further insights.

### Effects of hotregion size, count and cooperativity on the off-rate

In this work we have shown that indeed changes in the energies of hotspots upon mutations have a direct relationship with the off-rate. More so, changes at certain regions of the interface such as the rim may affect the off-rate differently depending on its size, whereas the core is a critical stability region for complexes of a wide range of size and interface areas. Hotspots tend to cluster into tightly packed regions and the conservation of this type of organization suggests that they are important for protein-protein association [37]. The aforementioned analysis however is not performed in relation to binding free energies or off-rates for protein-protein interactions. Therefore, it is still not clear to which extent, the presence, number and size of hotregions is advantageous to complex stability. Using the hotspot descriptors and the experimental off-rates, some insights into this can be gained.

#### Hotregion size, count and complex stability

Analysis of the mean PCCs for *No_Clusters* (the change in the number of hotregions upon mutation, R = −0.15) and *MaxClusterSize* (the change in size of the largest hotregion R = −0.09), show no notable contribution to changes in the off-rate (Table 2). Both the change in interface hotspot energy, and change in the number of hotspots show higher correlations (R = −0.51 and R = −0.44 respectively). For *RFSpotKFC2*, both *No_Clusters and MaxClusterSize* show higher correlations than the average (R = −0.29, for both), and the combination of the two descriptors into one using multiplication increases the PCC with log_10_Δ(*k_off_*) to R = −0.48. Nevertheless, its correlation of R = 0.6 with the change in hotspot energies (*Int_HS_Energy*), suggests that the underlying mechanism might still be the change in hotspot energies, irrespective of hotregion size and count. Note also that this does not imply that larger hotregions do not provide added stability to the complex, but rather their disruption is not critical to complex stability. Understanding if there is any advantage, when attempting to increase complex stability, in having larger hotregions, or more hotregions, would ultimately require analysis which controls for the number of hotspots, varies the number of hotregions or their size and assesses changes in the off-rate. However, current experimental data is limited in size and diversity for this to be performed comprehensively.

#### Hotregion cooperativity and complex stability

Probing the importance of the tendency for hotspots to cluster into hotregions, and for that matter, the importance of both size and number of hotregions for complex stability, has also to be done in the light of hotspot cooperativity. Cooperativity within hotregions has been suggested to be a natural consequence of the tight packing ratios found for hotspot residues in hotregions [37]. This adds another layer of complexity in validating the role of hotregions, as under cooperativity, larger hotregions do not necessarily contribute more to complex stability. In turn, this knowledge is critical in order not to overestimate or underestimate the contribution of hotspot energies within hotregions. There are two caveats to this, firstly we need to address the question of what type of cooperativity exists within the hotregions and complexes in the dataset, and secondly we need to have a function which can model or in this case account for it. To our knowledge, this is the first work to include energetic descriptors which account for potential cooperative effects in an empirical scoring function.

#### Diversity of cooperative effects

The approach taken here is that no assumption is made before hand for any type of cooperativity prevalent in the complexes and hotregions within our dataset. Rather the two hypotheses of positive cooperativity (*HSEner_PosCoop*) and negative cooperativity (*HSEner_NegCoop*) are investigated and compared to the baseline hypothesis of additive hotspot energies (*Int_HS_Energy*) – (We refer to these three descriptors as the cooperativity descriptors). The motivations and design of these descriptors are detailed in Materials and Methods, but effectively, the higher the PCC of these descriptors with the off-rate, the more likely it is that hotregions on the complexes of the 713 off-rate mutant dataset, show the given type of cooperative/additive effect. In Figure S2, the PCCs of cooperativity descriptors with Δlog_10_(*k_off_*) are highlighted for every hotspot predictor investigated. From these results, we find no evidence for a prevalent form of cooperativity in hotregions, as the additivity assumption works generally better than positive or negative cooperativity assumption. Several alanine scanning experiments on protein-protein interactions indicate that mutations are, to a large extent, naturally additive [62]–[64]. Eleven residues in the helix-turn-helix motif of the N-terminal domain of Gamma repressor, found in a region important for DNA binding, were substituted to alanine using binomial mutagenesis [64]. The additivity of two mutations was tested by comparing the observed and expected frequencies of the pairwise substitutions. Most of the pairwise substitutions occurred among the active sequences at frequencies close to the product of their single frequencies, thus confirming their additive nature. More so, a model assuming additive interactions was able to predict the activity class of mutants with 90% accuracy [64]. In similar fashion, nineteen residues within the hGH site 1 for binding to the hGHR were randomized using a combinatorial, shotgun alanine-scanning library [62]. On comparison of the counts of double alanine-mutations in hGH site 1 variants selected for binding to the hGHR , from the 144 pairwise combinations, only 15 pairs (10%) behave in a cooperative manner. Still, the analysis of such experiments is not limited to only hotspot residues, and therefore cannot be generalized to those of hotregions; for example combinatorial mutant analysis of the TEM1-BLIP complex which is performed on residues in tight packed modules, and hence more akin to hotregions, shows that residues within a cluster tend to show strong positive cooperativity [65]. With this in mind, our results in Figure S2 are dependent on both the definition of a contact and that of a hotregion. There is no rule of thumb on how to define a contact or hotregion; in one example, the distance between radii balls, with origins set on each C-α atom of the residues in question, is used to define a contact between two hotspot residues [37]. A hotspot residue is added to a hotregion cluster if it is in contact with at least two existing hotspot residues. Our definition uses a more fine-grain approach as a contact between two hotspot residues is created if any of their atoms are at a distance less than their van der Waals radii +0.5 Å. Though for hotspot residues to be added in an existing hotregion, it only needs to be in contact with any other of the hotspot residues, and therefore might be a more lenient way of adding hotspot residues to a hotregion cluster, which in turn may render less packed hotregions. Other contact methods also include weighted contacts according to whether side-chain or backbone atoms are in contact [65]. Most importantly, these different definitions generate different clusters of different packing ratios depending on their leniency and stringency, and therefore may affect the levels of cooperativity observed.

Another factor which may account for the inconclusiveness regarding the more prevalent form of cooperativity, is the modeling of cooperativity functions itself. Finding the right weights to apply to hotregions to account for cooperativity is not trivial as experimental data (such as [65]) is not common enough to be able to learn cooperativity functions from experimental data. Last but not least, the diversity of interactions within the dataset may be better characterized with different cooperativity functions. Interestingly, this diversity of cooperative effects is also observed in the GA-FS runs performed on subsets of related complexes (see Figure S4 in Text S3). Namely we observe that *HSEner_PosCoop*, *HSEner_NegCoop* and *Int_HS_Energy* tend to be important for different sets of related complexes in a mutually exclusive manner. This re-stresses the importance of detecting when a given type of cooperativity is present as much as it is important to model or account for it accurately.

#### Effects of cooperativity on effective energetic contribution of hotregions

**Figure 10:**
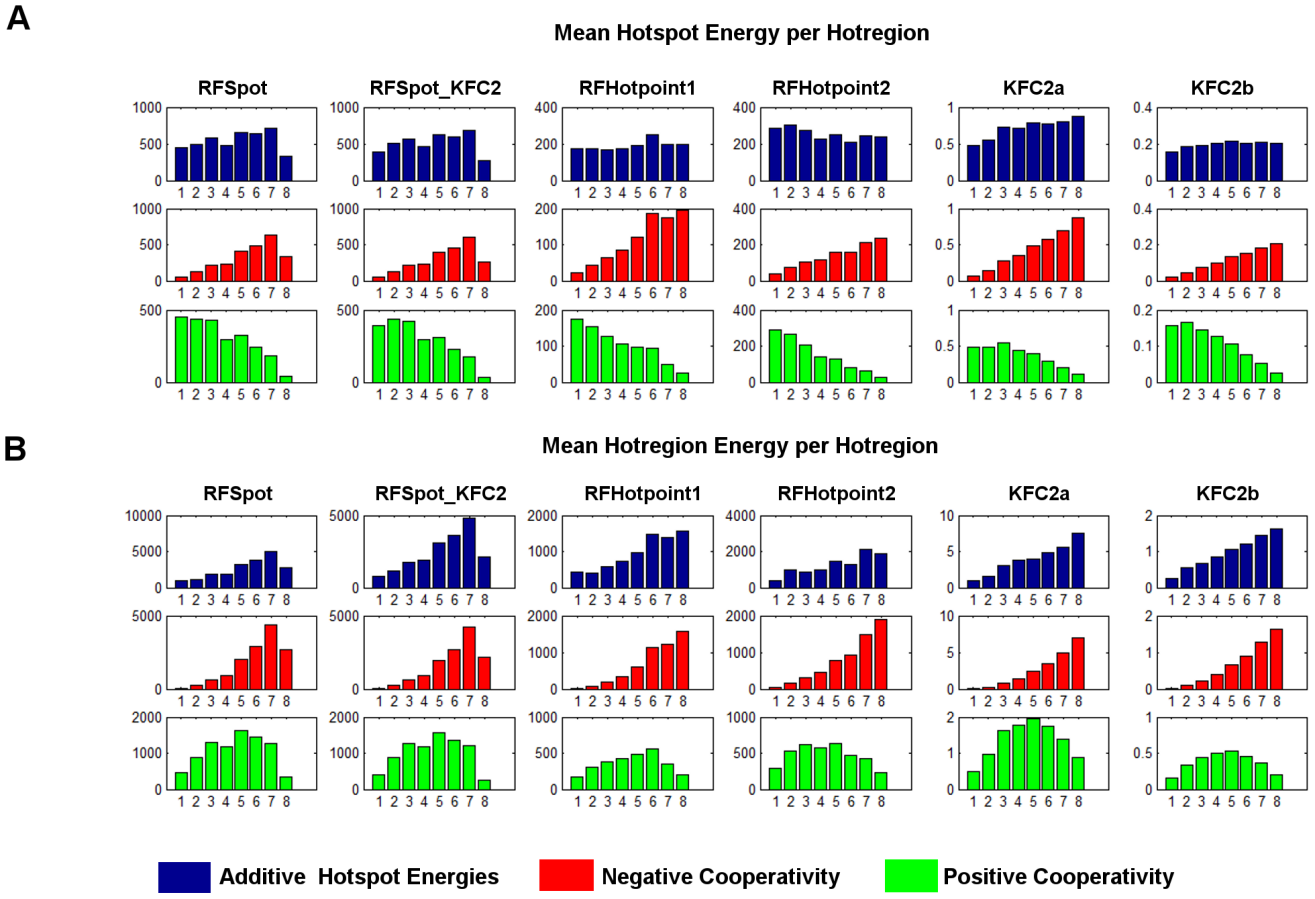
Effects of cooperativity on effective energetic contribution of hotregions. The summation of single-point alanine *ΔΔG*s of a hotregion may underestimate/overestimate its contribution if negative/positive cooperative effects are at play respectively. In this work, in order to account for potential cooperative effects, hotspot descriptors *HSEner_PosCoop*, *HSEner_NegCoop* apply linearly decreasing and increasing weights respectively to single-point alanine *ΔΔG*s within a hotregion. In turn *Int_HS_Energy*, based on the assumption the hotspot residues within the hotregion can be assumed to be additive, does not apply any weights. Here, the effects of accounting for cooperative/additive effects on the predicted hotspot and hotregions energies on all mutated complexes used in this work, is shown. (A) The mean hotspot energies for hotregion sizes of 1 to 8 hotspot residues. Each column shows the predictions of different hotspot predictors. (A) First row (blue), shows the raw mean hotspot energies, which essentially assumes all hotspots are additive within a hotregion. (A) Second row (red), assumes negative cooperativity within hotregions. To account for negative cooperativity, a linearly increasing weight is applied to the hotspot energies according to the size of the hotregion they are in (see Materials and Methods). (A) Third row (green), assumes positive cooperativity within hotregions and a linearly decreasing weight is applied to the hotspot energies according to the size of hotregion. (B) is similar to (A) but values are now the mean of the total hotregion energy of the given size. Effectively, the additive hotspot energy assumption results in hotregions contributing in a linearly increasing manner according to their size, the negative cooperativity assumption results in hotregions contributing in an increasing exponential-like manner as the hotregions increase in size, and the positive cooperativity assumption results in hotregions reaching a maximum contribution at around a hotregion size of 5, with their contribution decreasing beyond.

In order to understand better the effects of our cooperativity descriptors on the distribution of average hotspot energy, and the average hotregion energy for different hotregions sizes, we compare the average hotspot and hotregion energies of different hotregions sizes with no cooperative weightings (*i.e.* additive hotspot energy assumption), to those after positive cooperativity and negative cooperativity weightings are applied (Figure 10A–B). Analysis of the mean hotspot energies predicted by each hotspot predictor (first row in Figure 10A) shows a constant mean energy profile of hotspot energies within different hotregions. For the additive energy assumption (first row in Figure 10B) and the negative cooperativity assumption (second row in Figure 10B), a linear and exponential-like increase of energetic contribution from larger hotregions is shown respectively. For the positive cooperativity assumption, application of a linearly decreasing function on increasing hotregion sizes which have constant hotspot energies to start off with, results in a bell-shape contribution from hotregions. This suggests that maximum stability is provided by HR sizes of around 5; therefore, a saturation of HR contribution is achieved, beyond which larger hotregions do not necessarily increase complex stability.

### Off-rate prediction and conformational changes

**Figure 11:**
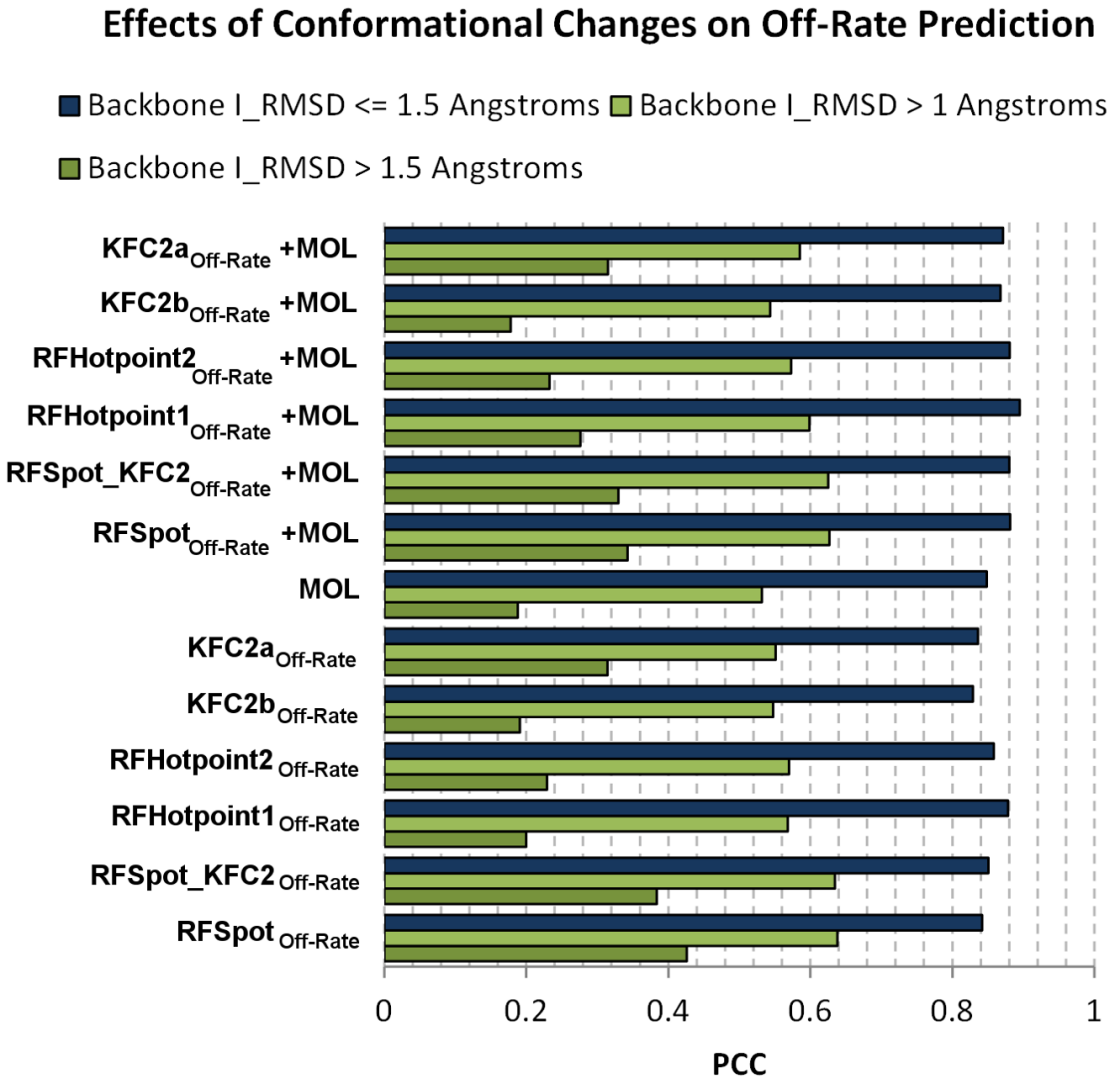
Effects of conformational changes and off-rate prediction. Predictions of the original 13 regression models developed for off-rate prediction. The predictions are assessed separately (PCC with Δlog_10_(*k_off_*)) for mutations on complexes which undergo significant backbone conformational changes of I_RMSD >1.5 Å (dark green), notable conformational changes of I_RMSD >1 Å (light green) and little to no conformational changes I_RMSD <1 Å (dark blue). Predicted accuracy is directly related to the magnitude of conformational change and becomes highly dependent on the model at higher levels of conformational changes. I_RMSD values were extracted from our previous work on the construction of a protein-protein affinity database [66].

Predictions of off-rate models are analyzed separately for mutations on complexes which undergo significant backbone conformational changes. The subset of complexes for which the unbound crystal structures of the *wild-type* complex are available, were singled out and their I_RMSD values for backbone conformational rearrangements were extracted from [66].This subset of complexes for which unbound crystal structures are available, amounts to 17 complexes and 332 mutations. 67 mutations on 4 complexes show significant conformational changes with (I_RMSD >1.5 Å) as defined in [66], and if the threshold is lowered to (I_RMSD >1 Å), this results in 119 mutations on 6 complexes. The PCCs for the off-rate model predictions with Δlog_10_(*k_off_*) are shown under three conformational change categories (Figure 11). The PCC, for complexes which show little to no conformational change (I_RMSD <1.5 Å), averaged over all prediction models, shows a correlation of R = 0.86, which decreases to R = 0.58 at (I_RMSD >1 Å) and R = 0.28 at (I_RMSD >1.5 Å). Though for the latter category, *RFSpot_Off-Rate_* achieves a correlation of R = 0.43. Changes in the different models are more apparent at complexes with higher conformational changes, most notably is the discrepancy in PCC between Molecular and *RFSpot_Off-Rate_* off-rate prediction models. This discrepancy is minimal at complexes with little conformational changes, ΔR = 0.01_I_RMSD <1.5 Å_ and increases to ΔR_I_RMSD >1 Å_ = 0.11 and ΔR_I_RMSD >1.5 Å_ = 0.24 for complexes with significant conformational changes. Reduction in the prediction accuracies for *wild-type* binding free energy prediction for complexes which undergo conformational change have also been noted [42], constituting an important challenge. Several factors may contribute to this, for example, complexes that are natively unstructured/disordered in local regions, may still remain disordered even in the bound state [67], [68]. Binding site variability has also been observed in certain complexes where the variability is not explained by experimental or procedural inaccuracies [69] and the off-rate may also be affected directly by the unbinding mechanism [70]. In all these examples, having a single snap-shot i.e. one conformational state for the complex we wish to calculate off-rate changes for, may not provide a picture comprehensive enough to predict off-rates. Methods for modeling conformational changes which in turn can be used to generate relevant snap-shots, are still one of the main limitations in current docking algorithms [71]. The generation of relevant snap-shots might also possibly involve the characterization of encounter complexes and their stability, where both the computational generation and experimental measurement of such states is still major challenge [50].

### Conclusions

In this work we take a comprehensive look at the determinants of complex dissociation in relation to interface hotspot energies and organization. Though the *ΔΔG* of a mutation may manifest itself as change in the off-rate as well as the on-rate [72], several lines of evidence suggest a dominant contribution from the off-rate [44]–[46]. Using experimental values on 713 mutations, in this work we also find evidence for a stronger relationship of *ΔΔG* with Δlog_10_(*k_off_*). More importantly, our investigations show that the change in the off-rate of a protein-protein interaction can be sufficiently explained by the re-distribution of hotspot energies caused by that mutation. Hence, the *ΔΔG* of single-point alanine mutations, and readily available hotspot predictors, can indeed be used as a starting point for the estimation of off-rate mutations to any residue type and also multi-point mutations. Given this, the novelty in our approach is in the way we quantify the effects of a mutation on the dissociation rate of a protein-protein interaction. Namely, instead of directly calculating a number of features pre- and post-mutation, a complete computational alanine scan is performed at the interface pre- and post-mutation. Using the single-point alanine energies from the scans we generate a set of hotspot descriptors which describe both local and global changes caused by the mutation in question. These include changes in the size and distribution of hotregions, cooperative effects within hotregions and changes in localized stability regions such as the core, rim and support regions. Using these sets of hotspot descriptors and a number of computational experiments, we are able to gain new insights into the determinants of protein-protein dissociation.

The predictive ability of the hotspot descriptors, in estimating Δlog_10_(*k_off_*), is first assessed independent of a learning model. Emphasis is given, both to numerical estimation and detection of stabilizing mutations (Δlog_10_(*k_off_*)<−1). As a benchmark comparison, the performance of the hotspot descriptors is compared to a diverse set of molecular descriptors, varying from physics-based energy terms to coarse-grain and atom-based statistical potentials. Here we find consistently higher predictive abilities for the hotspot descriptors, in estimating Δlog_10_(*k_off_*). The results suggest that both the synergistic and distributional information within hotspot energies may be exploited to uncover the more causative changes in complex stability. More importantly, it proposes an alternative way of modeling single-point and multi-point mutations to any residue type, which is that of mapping them to functions using only alanine *ΔΔG* energies.

To assess the predictive abilities of hotspot descriptors when combined in learning models, several machine learning models trained on Δlog_10_(*k_off_*) are also investigated. The best regression model, which combines both molecular and hotspot descriptors, *RFSpot_KFC2_Off-Rate_+Mol*, achieves a PCC of 0.79 with experimental off-rates. Model predictions are also assessed on different subsets of mutations defined as data regions. The data regions enable us to identify, classes of mutations which are consistently harder to characterize, data set biases and prediction patterns. We find that core and multi-point mutations are the most accurately predicted; however, mutations at rim regions are consistently harder to characterize. In terms of the prediction of stabilizing mutations, a pattern emerges where mutants to alanine which stabilize the complex are harder to detect. To uncover relationships between different subsets of off-rate mutations and descriptors, we develop linear and non-linear feature-selection models trained on data-regions. Descriptor-data region networks generated from these models, enable us to identify descriptors highly specific to certain classes of mutations and those which are broadly important to a number of different regions simultaneously.

The results gained in this work are particularly useful from a computational design perspective. Off-rate classification models for stabilizing mutation prediction (Δlog_10_(*k_off_*)<−1), achieve a MCC of 0.59, which increases to 0.82 when neutral mutations are excluded. We find that hotspot descriptors which are able to capture the intricacies of off-rate changes related to the re-distribution of hotspot energies and positive cooperative effects play a key role in detecting such mutations. Secondly, we underline the importance of performing a computational alanine scan, if possible, before optimizing an interface. This presents a distributional context that one may exploit and apply mutations accordingly, and thus adopt a biomimetic design strategy mirroring that taken by evolution. For example, our results indicate that the distribution of the critical stability regions across protein-protein interfaces is a function of complex size. Though large-size complexes investigated here show more robustness to mutations than small-size complexes, here we show the insensitivity to mutations is not shared equally across all parts of the interface, as changes in the core can still significantly affect complex unbinding for large complexes. Conversely for small complexes, the increase in insensitivity to mutations is distributed homogenously across the interface, with hotspots in the rim region becoming jointly critical for complex longevity. This suggests that the accurate characterization of rim hotspots is important in the design of small complex interfaces. Further advances in characterization of off-rate mutations are likely to be achieved upon improved modeling of cooperative effects within hotregions and that of conformational changes.

## Materials and Methods

### Method summary

Six hotspot prediction algorithms (*RFSpot*, *RFSpot_KFC2*, *RFHotpoint1*, *RFHotpoint2*, *KFC2a* and *KFC2b*) are used for the generation of hotspot descriptors, which are subsequently used for the prediction of off-rates. The method is explained in Figure 1 and requires that the hotspot predictor in question generates a prediction for each residue at the interface, both pre- and post-mutation, akin to an alanine-scan. The energies from single-point alanine mutations of the pre- and post-mutation scans are then used to calculate a set of 16 hotspot descriptors. For each hotspot predictors, its own set of hotspot descriptors is generated. The hotspot descriptors enable us to use the energies from single-point alanine mutations, particularly those which are hotspots, in order to describe the effects of off-rate mutations to non-alanine mutations and also multi-point mutations.

### SKEMPI alanine dataset

The prediction of hotspots is an active area of research and several hotspot prediction algorithms have been developed [25]–[35]. One short-coming of these algorithms is that they have been trained and tested on very limited alanine scanning databases, namely ASEdb [73] and BID [74]. The shortcoming of these datasets as benchmarks has been highlighted in [25], [41]. To address these limitations we recently assembled the largest database of mutations to date, with 3047 experimentally determined structures and binding kinetics, including free energy changes, dissociation/association rates and enthalpies/entropies where available [41]. All single-point alanine mutations, limited to the complex interfaces, were extracted from the SKEMPI database. This totals to a set of 635 non-redundant mutations with experimental *ΔΔG* in 59 different complexes and 154 hotspot residues with *ΔΔG* > = 2 kcal/mol (Table S16 in Text S4). All hotspots represent the positive training examples and anything, which is not a hotspot (*ΔΔG* <2 kcal/mol) as negative training examples.

### Hotspot predictor design and performance analysis

#### RFSpot and RFSpot_KFC2

For each training example in the SKEMPI alanine dataset, and hence *wild-type* complex PDB structure, a number of molecular descriptors, describing various aspects of the interaction, were calculated. These descriptors have already proven successful in our previous work related to the prediction of *wild-type* binding free energies [42] and *wild-type* kinetic rate constants [19]. A full list and explanation of the molecular descriptors can be found in the Text S1. After calculation of the molecular descriptors on the *wild-type* complex PDB structure, each respective structural mutation was made using FoldX [43] and the same set of molecular descriptors recalculated. Each descriptor, fed into the learning model, is determined as the difference between the mutant and *wild-type* descriptor value:
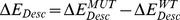
(4)


As a learning algorithm the RF classifier model is employed [75], using 1000 trees and an mtry (*i.e.* number of random variables sampled as candidates for a split) of 15. The RF learner is well suited for high dimension datasets, such as the one described here with 110 features. Throughout the manuscript, we refer to this RF hotspot classifier algorithm as *RFSpot*. *RFSpot_KFC2* is a similar classifier model to *RFSpot* with the difference that it adds to the 110 molecular features set, 13 features from the original *KFC2a* and *KFC2b* models. These include: *res_hp*, *pos_per*, *delta_tot*, *core_rim*, *rot5*, *plast4*, *plast5*, *fp10* from KFC2a and *res_size*, *ratio5*, *rot4*, *hp5*, *fp9* from *KFC2b*. Details on the calculation of each specific descriptors are described in [30], most notably they include features which position the mutation using solvent accessibility. This enables the model to exploit the fact that hotspots tend to occur in regions of low solvent accessibility [24] and is the key difference between *RFSpot* and *RFSpot_KFC2*.

#### RFHotpoint1 and RFHotpoint2, KFC2a, KFC2b

Similar to *RFSpot* and *RFSpot_KFC2*, *RFHotpoint1* and *RFHotpoint2*, are RF hotspot classifiers trained on the SKEMPI alanine dataset which use only features from the original *Hotpoint* server as features, these include: *relativeComplexASA*, *relativeMonomerASA*, *pairPotential*, *complexASA* as described in [28]. *RFHotpoint2* differs from *RFHotpoint1* in that for the former, the threshold is lowered to allow for more hotspot detections at the cost of a higher FPR. The reason behind developing the *RFHotpoint* models is due to the fact that the original *Hotpoint* server does not associate an energetic or confidence value to its hotspot prediction, hence hotspot descriptors which make use of hotspot energies cannot be calculated. *RFHotpoint* models therefore enable us to use *Hotpoint* features, trained on a larger dataset of SKEMPI instead of ASEdB (as in the original Hotpoint algorithm) and most importantly, associated confidence values to our hotspot predictions using the RF model. To validate *RFHotpoint1* and *RFHotpoint2* as a representative alternative to *Hotpoint*, predictions from *Hotpoint* server were generated for the SKEMPI alanine dataset. Any predictions for mutations in ASEdB were also removed since Hotpoint uses ASEdB as training data. The predictions are compared to the 20-fold test predictions of *RFHotpoint1* and *RFHotpoint2* for the same mutations and classification results are presented in Table S15 in Text S4. Both *RFHotpoint1* and RFHotpoint2 achieve higher MCCs than *Hotpoint*. For *KFC2a* and *KFC2b*, no models needed to be re-trained again, as the original predictions from the *KFC2* server have associated with them an energetic value which can be directly used for the calculation of the hotspot descriptors.

#### Hotspot energies

In the RF classifier model used in *RFSpot*, *RFSpot_KFC*, *RFHotpoint1* and *RFHotpoint2*, each tree in the 1000-tree forest makes is own class prediction (Hotspot/Non-Hotspot) of the mutation in question. The class which accumulates the majority of tree-votes is the predicted class, and the difference in the number of votes for the hotspot class relative to the non-hotspot class (Votes_Hotspot_- Votes_Non-Hotspot_) indicates the model's confidence in the predicted class. In this work, these confidence values are used as an estimation of hotspot *ΔΔG*s. The rational is that the higher the confidence value, the more trees have predicted this to be a hotspot, implying that larger numbers of different feature subsets consider this to be a hotspot also. Given that several different aspects of the protein interaction have vouched for the example to be a hotspot, we expect the hotspot *ΔΔG* to be larger. To confirm this, RF regression models are trained on the same training data as *RFSpot, RFSpot_KFC2, RFHotpoint1 and RFHotpoint2* RF classifiers, in order to generate true *ΔΔG* predictions and compared to the confidence values generated by each of them. Note that *RFHotpoint1* and *RFHotpoint2* use the same confidence values and only differ by their threshold on those confidence values; hence one correlation for the confidence values is presented for both. The confidence values of the classifier models, show correlations of R = 0.88, R = 0.86, R = 0.86 with the regression models' *ΔΔG* predictions for *RFSpot*, *RFSpot_KFC2* and *RFHotpoint1&2* respectively. Therefore, apart from the differences in their absolute values, the confidence values do provide relative values which have a direct linear relationship to *ΔΔG*. On assessment of the MCCs of the regression RF models at a threshold of > = 2 kcal/mol, the regression models achieve lower MCCs to that of the classifier models, all of which are the result of higher FPR. Given that 75% of the *ΔΔG* data is of the negative non-hotspot class, minimal increases in the FPR add a significant number of false-positives which would subdue the gain of additional hotspots correctly detected. Therefore, the use of a classifier in *RFSpot, RFSpot_KFC2, RFHotpoint1 and RFHotpoint2*, enables us to achieve a lower false positive rate, to that of a regression model, but still be able to have confidence values which relate directly to *ΔΔG*. For the sake of simplicity, we refer to the *ΔΔG* confidence values extracted by the method described here as *ΔΔG*s throughout the manuscript.

#### Performance of hotspot predictors on SKEMPI alanine dataset

The predictive accuracy of the hotspot predictors from which the hotspot descriptors are generated from (i.e *RFSpot*, *RFSpot_KFC2*, *RFHotpoint1*, *RFHotpoint2*, *KFC2a* and *KFC2b*), is assessed on the SKEMPI alanine dataset using a number of classification performance measures. For *RFSpot*, *RFSpot_KF2*, *RFHotspoint1* & *RFHotpoint2*, the predictions results from a 20–Fold CV are used, whereas for KFC2a and KFC2b the predictions from KFC2 [30] server are used. Note that for *KFC2a* and *KFC2b*, the predictions for the data which is in SKEMPI and not in ASEdB is presented, as KFC2 server algorithm uses ASEdB mutations for model design and training. The predictions are compared to a number of hotspot prediction algorithms (KFC2 [30] HotPoint [28], Robetta [35], RFMirror [32], and TSVM [31]). Details on each hotspot predictors and the sources of their predictions are presented in Table S10 in Text S4. The performance of each hotspot predictor is shown in Table S11 in Text S4 and a list of their predictions in Table S16. Note however that Table S11 in Text S4, though ranked according to MCC, shows their performance on different mutations, and therefore cannot be relatively compared. A relative comparison between two predictors can only be performed on the intersections of mutations for which both algorithms provide unbiased predictions, which is beyond the scope of this work. However, this comparison is made for the two hotspot predictors developed in this work namely *RFSpot* and *RFSpot_KFC2*. Table S13 in Text S4 shows the performance of *RFSpot* against all other hotspot predictors on their data intersection, similarly for *RFSpot_KFC2* in Table S14 in Text S4. Though *RFSpot* excels at having a low false-positive-rate (FPR), its true-positive-rate (TPR) is compromised. We note that some hotspot prediction algorithms introduce features, which are based on statistical tendencies of hotspots [30], [32]. For example, the inclusion of W, Y, R as features, since all three show a predisposition to be hotspot residues [36], or the inclusion of the accessible surface area of the residue in question, where it is known that hotspots have a predisposition to occur at the core of the interface [24]. To maintain an unbiased prediction scheme, based purely on molecular and physical descriptors, we intentionally avoid the inclusion of such descriptors in *RFSpot*, and this is the probable reason for its low TPR. It is understood that the addition of descriptors which relate to solvent accessibility may increase the TPR of *RFSpot* as this would enable the RF learner to distinguish between mutations performed at the core as opposed to those at the rim, where less hotspots occur [24]. Indeed, it has been shown that a predictor with just 3 solvent accessibility features can result in a sensitivity of 0.87 (*i.e.* TP/(TP+FN)) for the BID test set [30]. This is also confirmed using *RFSpot_KFC2* which introduces features that relate to solvent accessibility and upon setting the threshold to achieve the same FPR to that of *RFSpot*, the TPR is increased from 0.27 (in *RFSpot*) to 0.49 (in *RFSpot_KFC2*). With this in mind, low solvent accessibility is not a sufficient indicator of hotspots as most residues at the core are still non-hotspots [24], [30]. Such models are biased towards predicting hotspots at the core regions as a result of a statistical tendency, and may lay the risk of not being able to detect mutations in other regions as accurately. The risk is even higher if the training set is small and other regions outside the core are underrepresented. Though *RFSpot_KFC2* uses such solvent accessibility descriptors, the model uses other molecular features and is trained on a more diverse alanine dataset of SKEMPI as opposed to the ASEdB. Therefore, though still present, the bias towards the prediction of hotspots towards the core is not as strong.

### Hotspot descriptor calculation and dataset

As depicted in Figure 1, for any given complex, a computational alanine scanning is first performed on the *wild-type* interface using a hotspot prediction algorithm. This enables calculation of the set of hotspot descriptors described in Table 1. The respective single-point or multi-point mutation is then applied using FoldX [43], and another computational alanine scan is performed on the mutated interface, again using the same hotspot prediction algorithm invoked for the *wild-type* scan, from which a new set of hotspot descriptors are calculated. The energetic value contributed by each hotspot descriptor is then the difference in its energetic value pre- and post-mutations:
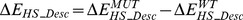
(5)


The hotspot descriptors are calculated for a set of 713 mutations from the SKEMPI database [41]. Therefore, in total, for each hotspot prediction algorithm, we make 50 *wild-type* and 713 mutant computational alanine scans. To ensure that off-rate predictions are not made *via* hotspots models trained on the same examples, all 713 computational alanine-scans made by *RFSpot, RFspot_KFC2, RFHotspoint1 and RFHotspoint2* are strictly 20-Fold-test predictions for mutations common between the off-rate and hotspot datasets, and test predictions for the rest. Therefore, all hotspot predictions on which the hotspot descriptors are calculated are unbiased and not susceptible to over-fitting. Each mutation in the 713 off-rate mutant dataset has available the experimental *wild-type* and mutant off-rates and the respective PDB structure. This off-rate dataset is the largest assembled to date and experimental off-rates within this set range cover a range of Δlog_10_(*k_off_*) of −8.5 to 6.8, with *k_off_* units of s^−1^ and represent a diverse set of interactions as listed in (Dataset S1).

### Hotspot descriptors

Hotspots provide a very rich source of information, which can be exploited on many levels. Firstly, the occurrence of a hotspot is not limited to any particular physical phenomena. Instead hotspots are the result of the synergistic effect of different phenomena together. These may include evolutionary pressures, along with physicochemical and structural properties [76]. Thus, mapping all the critical points for each to an interface produces a complex distribution. However, the description of an interface though hotspots is conceptually much simpler. From a computational stand-point, the advantage is that one is able to represent an interface with a much smaller set of features without compromising accuracy, as the effects of several phenomena is still encompassed within the hotspots themselves. This reduction in feature set size is also particularly attractive in the context of learning algorithms. A second attractive attribute of hotspots is their distributional properties. Hotspots tend to cluster into hotregions, within which, hotspots are suggested to be energetically cooperative [37], [65]. It has also been shown that hotspots tend to occur more at the core regions as opposed to the rims; however, low solvent accessibility is not a sufficient property for a residue to be a hotspot [24]. Understanding how these two aspects of hotspot structure and organization, relate to the off-rate of a complex, is critical for an accurate characterization of changes in the off-rate caused by mutations. The aim of the hotspot descriptors designed here (Table 1) is therefore to present hotspots in different positional contexts, which may affect complex destabilization to differing degrees. The relevance of each descriptor to off-rate variation is then assessed with different feature importance measures and the key determinants of the dissociation process reported.

#### Interface hotspot descriptors

*Int_Energy_1* is the difference in the sum of the single-point alanine *ΔΔG*s of all interface residues N, pre- and post-mutation.
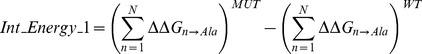
(6)
*Int_HS_Energy* is the difference in the sum of the single-point alanine *ΔΔG*s of all hotspot residues N_HS_, pre- and post-mutation.
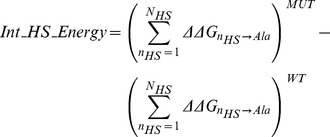
(7)
*No_HS* is the change in number of hotspots predicted at the interface pre- and post-mutation. This can be considered to be a coarse-grained version of *Int_HS_Energy*.

#### Solvent accessible region hotspot descriptors

To account for the different solvent accessible regions where hotspots may occur at the interface, the following hotspot *ΔΔG*s are summed separately for the core, rim and support regions and termed as *CoreHSEnergy*, *RimHSEnergy* and *SuppHSEnergy* respectively. Therefore, these hotspot descriptors are similar to *Int_HS_Energy* but limited to counting *ΔΔG* for hotspots that fall in the given region. In addition, *CoreHS*, *RimHS* and *SuppHS* desfcriptors, count the hotspot changes within each region. Again these can be considered as coarse-grained versions of their respective counterparts. The core, rim and support regions of the complex interface are defined according to [77]. Core residues are generally exposed in the unbound configuration but buried in the bound state. Rim regions are generally exposed in both the bound and unbound states whereas support residues are generally buried in both states. The thresholds chosen in defining these regions are such that each region has a similar number of residues [77].

#### Hotregion cooperativity descriptors

The cooperativity of a pair of residues m_1_ and m_2_, can be calculated by comparing the gain of adding each residue separately from a neutral reference state of both *wild-type* residues mutated to alanine (*ΔΔG*_A1,A2→A1,m2_+*ΔΔG*_A1,A2→m1,A2_) to that of adding both residues concurrently, given the same reference state(*ΔΔG*_A1,A2→m1,m2_) [78]. Namely, let A_1_ and A_2_ represent the alanine mutation of m_1_ and m_2_ respectively, then
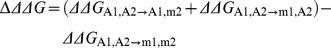
(8)


If Δ*ΔΔG* is positive, this indicates positive cooperativity as the contribution of both residues together is more stabilizing than the sum of their parts. Conversely if the Δ*ΔΔG* is negative, this indicates negative cooperativity, whereas if the Δ*ΔΔG* is close to zero, then such pairs can be considered to be effectively independent of each other hence their contributions to be additive in relation to each other.

Expanding *ΔΔG*_A1,A2→A1,m2_ and *ΔΔG*_A1,A2→m1,A2_ we get
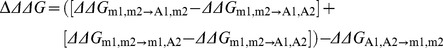
(9)

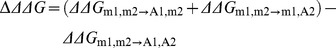
(10)


In this work, we only make single point-mutations during the alanine scan and calculate the energetics associated with such complex states as in eqn (10): *ΔΔG*_m1,m2→A1,m2_ and *ΔΔG*_m1,m2→m1,A2_. The summation of these energies is then used as an estimate of the off-rate. If hotspots within a cluster are additive, then the summation of *ΔΔG*_m1,m2→A1,m2_+*ΔΔG*_m1,m2→m1,A2_ would be a sufficient estimate of the cluster's contribution to the off-rate. However if m1 and m2 are positively cooperative, then their contribution towards the off-rate using the summation *ΔΔG*_m1,m2→A1,m2_+*ΔΔG*_m1,m2→m1,A2_ would be an overestimate of the true contribution *ΔΔG*_m1,m2→A1,A2_, hence the positive value for Δ*ΔΔG*. Therefore in this case, to account for positive cooperativity we down-weight the summation of *ΔΔG*_m1,m2→A1,m2_+*ΔΔG*_m1,m2→m1,A2_. Conversely if m1 and m2 are negatively cooperative, then a positive weighting would be more suitable to account for the underestimation. Further, higher order cooperativity effects involving three or more residues are known [78] and it is likely that many binding modules exhibit such complexity, where it is not possible to decouple the contributions from each individual residues. However, if we assume that cooperativity effects are taking place, the weighting applied should also reflect the number of residues suspected to be cooperative. With this in mind, the cooperativity hotspot descriptors are designed as follows; given a set of predicted hotspots at the interface, each hotspot is categorized according to the hotregion cluster size it is found in. As *Int_HS_Energy* assumes hotspot contribution is additive, the sum of the hotspot energies is independent of the hotspot locations eqn (7). On the other hand, *HSEner_PosCoop* and *HSEner_NegCoop* are the sum of the hotspot energies downweighted/upweighted using simple linearly decreasing/increasing functions related to the size of the hotregion the given respective hotspot is in:
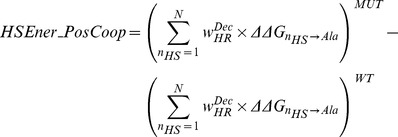
(11)

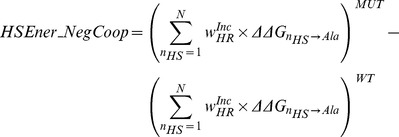
(12)where w_HR_^Dec^ = (0.125, 0.25, 0.375, 0.5, 0.625, 0.75, 0.875, 1) and w_HR_^Inc^ = (1, 0.875, 0.75, 0.625, 0.5, 0.375, 0.25, 0.125) for hotspot n_HS_ in a hotregion of sizes (HR = 1, 2, 3, 4, 5, 6, 7, 8+) respectively. Though more complex non-linear weightings could be investigated, such as ones fitted to the off-rate data itself, this would require sacrificing parts of the data for fitting. With this in mind, all hotspot descriptors designed in this work were independent of any off-rate data. Coarse-grained versions *HS_PosCoop* and *HS_NegCoop*, which weight hotspot counts instead of energies, are also implemented in the model. One should note that since the energetic contribution of a hotregion taken as a whole is considered to be additive and independent of other hotregions [37], [65] we only aim to investigate and account for intra-hotregion cooperativity using these descriptors as opposed to inter-hotregion cooperativity.

#### Hotspot coverage related descriptors

Other hotspot descriptors relate to the spread of hotspots across the interface. The intuition here is that a heterogeneous distribution of hotspots across the interface might be more beneficial to complex stability than if hotspots where concentrated onto a specific region of the interface only. *AVG_HS_PathLength* is the average path length between all possible pairs of hotspots at the interface, normalized to the average path length of all possible pairs of a random set of residues at the interface. The path length between two residues is calculated as the least number of contacting residues linking them together. Two residues are considered to be in contact if any of their atoms are at a distance smaller than the sum of their van der Waals radii +0.5 Angstroms. *No_Clusters* counts the number of unique hot regions, where it is likely that more hotregions may span the interface given that separate hotregions are not in contact. *MaxClusterSize* counts the change in the number of hotspots in the largest hotregion.

#### Definition of a hotregion

Some of the hotspot descriptors use hotregion information within them (*No_Clusters*, *MaxClusterSize*, *HSEner_PosCoop/HS_PosCoop* and *HSEner_NegCoop/HS_NegCoop*). A hotregion is created whenever two or more hotspot residues are in contact. Two hotspot residues are considered to be in contact if any of their atoms are at a distance smaller than the sum of their van der Waals radii +0.5 Å. A hotspot residue is added to an existing hotregion, if any of its atoms makes contact with any of the hotspot residues already in the hotregion.

### Feature importance measures

The importance of the descriptors used in this work, in relation to the dissociation rates, is assessed using three methods. The first method is the global correlation of a given descriptor with the target variable, which in this case is the experimental off-rate Δlog_10_(*k_off_*). To calculate this, the Pearson's Correlation Coefficient (PCC) is used. A second method is the Mann-Whitney U-test, which checks whether a set of two independent observations have smaller or larger values than the other. The test is used to assess the coarse-grain predictive power of our descriptors in discriminating between stabilizing mutants from neutral to destabilizing mutants. Several other classification related measures are used for this same purpose also, namely:

**True-Positive-Rate (TPR)/Recall:** 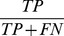


**False-Positive-Rate (FPR):** 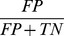


**Specificity:** 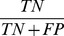


**Precision:** 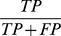


**Accuracy:** 
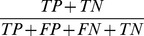


**Matthew's Correlation Coefficient (MCC):** 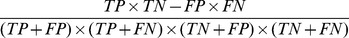


**F1-Score:** 
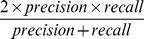


where TP = True-Positive, FP = False-Positive, TN = True-Negative, FN = False-Negative. A third method used is an assessment of descriptor importance in the context of a learning model where several of the descriptors are combined together to make a prediction. For this the built-in Random Forest Feature Importance measure (RFFI) is used [75]. Note that unlike the PCC, Mann-Whitney U-test and the above mentioned classification measures, the RFFI calculates feature importance as a function of other features in the model.

### Data regions

The 713 off-rate mutations from SKEMPI are also subdivided into the following data regions for analysis: Single-Point (SP) alanine mutations, 361; SP non-alanine mutations, 155; SP mutations, 516; Multi-Point (MP) mutations, 197; SP mutations to polar (Q, N, H, S, T, Y, C, M, W) residues, 39; SP mutations to hydrophobic (A, I, L, F, V, P, G) residues, 309; SP mutations to charged (R, K, D, E) residues, 68; mutations exclusively on core regions, 272; rim regions, 79; support regions, 114; mutations on complexes of Large-Interface-Area (>1600 Å^2^) , 355 and Small-Interface-Area (<1600 Å^2^), 358.

### Genetic Algorithm Feature Selection (GA-FS)

The GA-FS algorithm runs feature selection on subsets of the off-rate mutation dataset defined as data regions. Two separate GA-FS runs are performed, one for Linear Regression models and another for Support Vector Machine (RBF) Regression Models (using the *LIBSVM* package). Two separate 10-fold cross-validation loops are used. One to assess prediction accuracy on the off-rate mutations for the given data region and the second to derive the optimal feature set. A 10-fold inner-cross validation loop is used within the GA-FS fitness function to drive the feature selection process with reference to Pearson's Correlation Coefficients. After the GA has converged, the LR/SVM model is tested for its accuracy on the outer-loop fold. This process is repeated 10 times such that all 10 outer loop folds are used as a test set validation for the final model. Therefore, the accuracy of the final model is tested on data which is not used to derive the feature set. As an initial feature set available for selection, 110 molecular descriptors and 16 hotspot descriptors from the best performing off-rate prediction model *RFSpot_KFC2* are available. A fixed feature set size of 5 is chosen so as to avoid overfitting on smaller sized data regions. Therefore, the genome size for the GS-FS (LR) is 5 whereas that for GA-FS (SVM) is 7 to also optimize the cost and gamma parameters of the RBF. Available cost parameters values are quantized into 111 bins ranging from 2^−5^ to 2^6^. Gamma parameter values are quantized into 1300 bins ranging from 2^−8^ to 2^5^. The GA's initial population size was set at 1000 individuals, and generated such that the initial population included at least one instance of each of the 126 features. Tournament selection was employed with a size of 8 individuals. Uniform random crossover was used with a crossover fraction set to 50% and a mutation rate that exponentially decreased as the number of generations applied increased. Note that for each data region 50 separate GA-FS runs were performed.

### Off-rate Classification Data Sets (CDS1 and CDS2)

To assess the discriminatory power of the hotspot and molecular descriptors, the 713 off-rate mutations are partitioned into (Δlog_10_(*k_off_*)<−1), representing the stabilizing portion of the dataset, and (Δlog_10_(*k_off_*)>0), representing the neutral to destabilizing portion of the dataset (referred to as CDS1 –Classification Dataset 1). The motivations behind the thresholds of CDS1 are two-fold. Firstly, previous error estimates show that experimental noise in the data can be as high as 2kcal/mol [41], [42]. Experimental noise causes miscategorization errors when converting Δlog_10_(*k_off_*) from continuous values to categorical bins, and therefore, the exclusion of data-points within [−1, 0] should reduce sufficiently the number of miscategorization errors between stabilizing and neutral/de-stabilizing mutations. Secondly, being able to detect stabilizing mutations from neutral ones is an important aspect of interface design. As shown in Figure S1, the range within [0, 1] contains 43% of the data. Therefore, the removal of Δlog_10_(*k_off_*) within the range [−1,0] still allows a sufficient amount of neutral mutations. This data subset, results in a dataset of 501 neutral to destabilizing mutations (referred to as non-stabilizing mutations) and 31 stabilizing mutations (See Dataset S2). To further investigate the discrimination ability of the descriptors, an additional threshold satisfying |Δlog_10_(*k_off_*)| >1 is also investigated (Dataset S3). This dataset which removes most of the neutrals is referred to CDS2.

## Supporting Information

Dataset S1 **Off-rate dataset (713 mutants).**
(CSV)

Dataset S2 **CDS1 (534 stabilizing vs. neutral to destabilizing mutants).**
(CSV)

Dataset S3 **CDS2 (244 stabilizing vs. destabilizing mutants).**
(CSV)

Figure S1 **Distribution of Δlog_10_(** ***k_off_*** **) values in SKEMPI.** Distribution of Δlog_10_(*k_off_*) shows that the data is biased towards destabilizing mutations (Δlog_10_(*k_off_*)>0) and a significant portion of the data consists of neutral to stabilizing off-rate mutants (0<Δlog_10_(*k_off_*)<1).
(PDF)

Figure S2 **PCCs of hotspot cooperativity descriptors with Δlog_10_(k_off_).**
(PDF)

Table S1 **Off-rate model regression predictions.**
(CSV)

Table S2 **Off-rate model classifier predictions (CDS1).**
(CSV)

Table S3 **Off-rate model classifier predictions (CDS2).**
(CSV)

Table S4 **Descriptor performance measures CDS1.**
(CSV)

Table S5 **Descriptor performance measures CDS2.**
(CSV)

Text S1 **Molecular descriptors set.**
(PDF)

Text S2 **Hotspot descriptor consistencies and biases.**
(PDF)

Text S3 **Effects of cross validation routine.**
(PDF)

Text S4 **Hotspot predictor predictions and performance.**
(PDF)

Text S5 **Descriptor performance measures (PCC_UTest_AUC).**
(PDF)
