# Supplementary figures and images for: Characterizing Changes in the Rate of Protein-Protein Dissociation upon Interface Mutation Using Hotspot Energy and Organization
Source: PLoS Comput Biol. 2013 Sep 5;9(9):e1003216. doi: 10.1371/journal.pcbi.1003216 (PMC3764008; doi:10.1371/journal.pcbi.1003216)

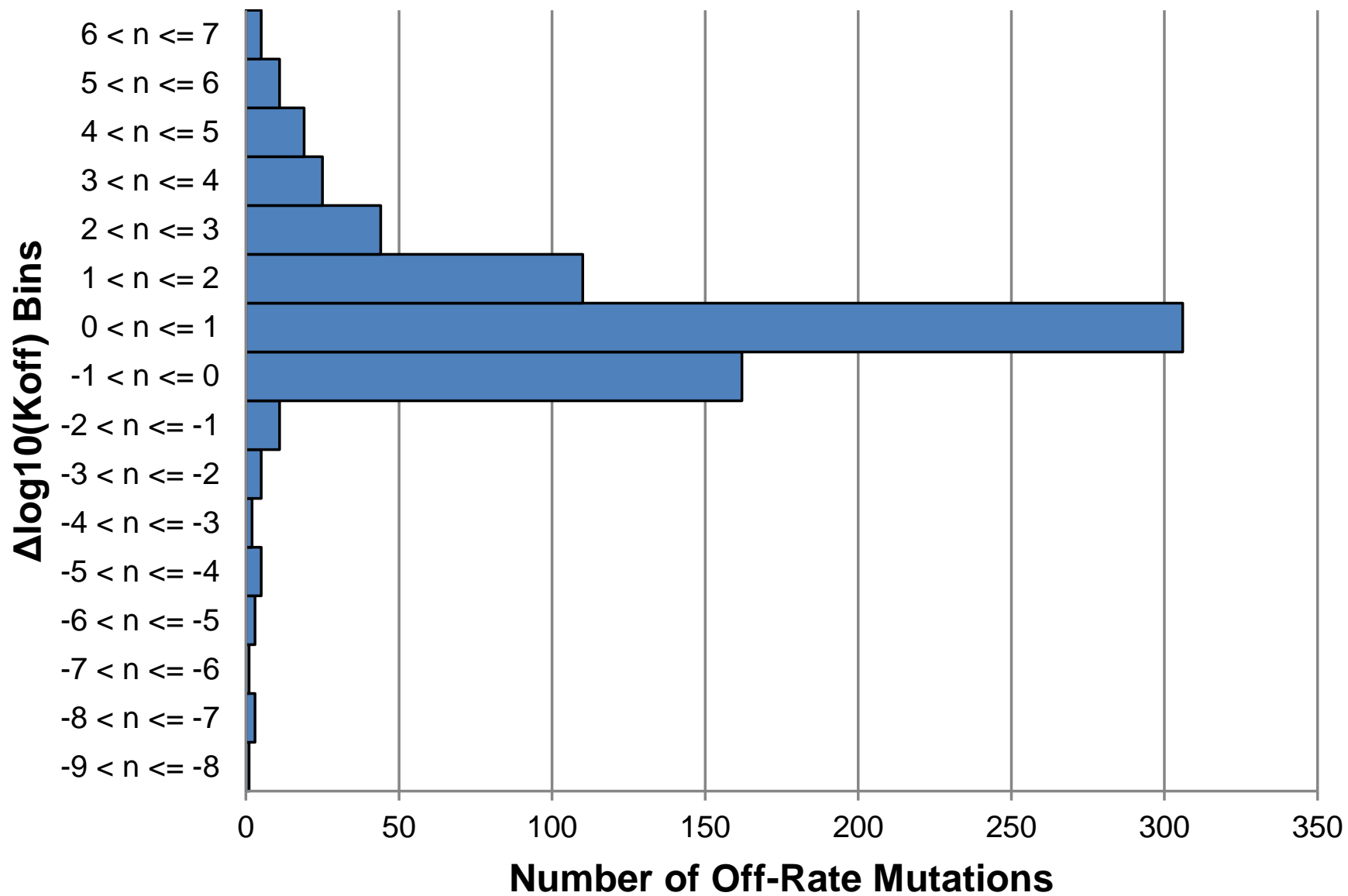

Supplement: Figure S1 — Distribution of Δlog10( koff ) values in SKEMPI. Distribution of Δlog10(koff) shows that the data is biased towards destabilizing mutations (Δlog10(koff)>0) and a significant portion of the data consists of neutral to stabilizing off-rate mutants (0<Δlog10(koff)<1). (PDF) [file pcbi.1003216.s004.pdf]

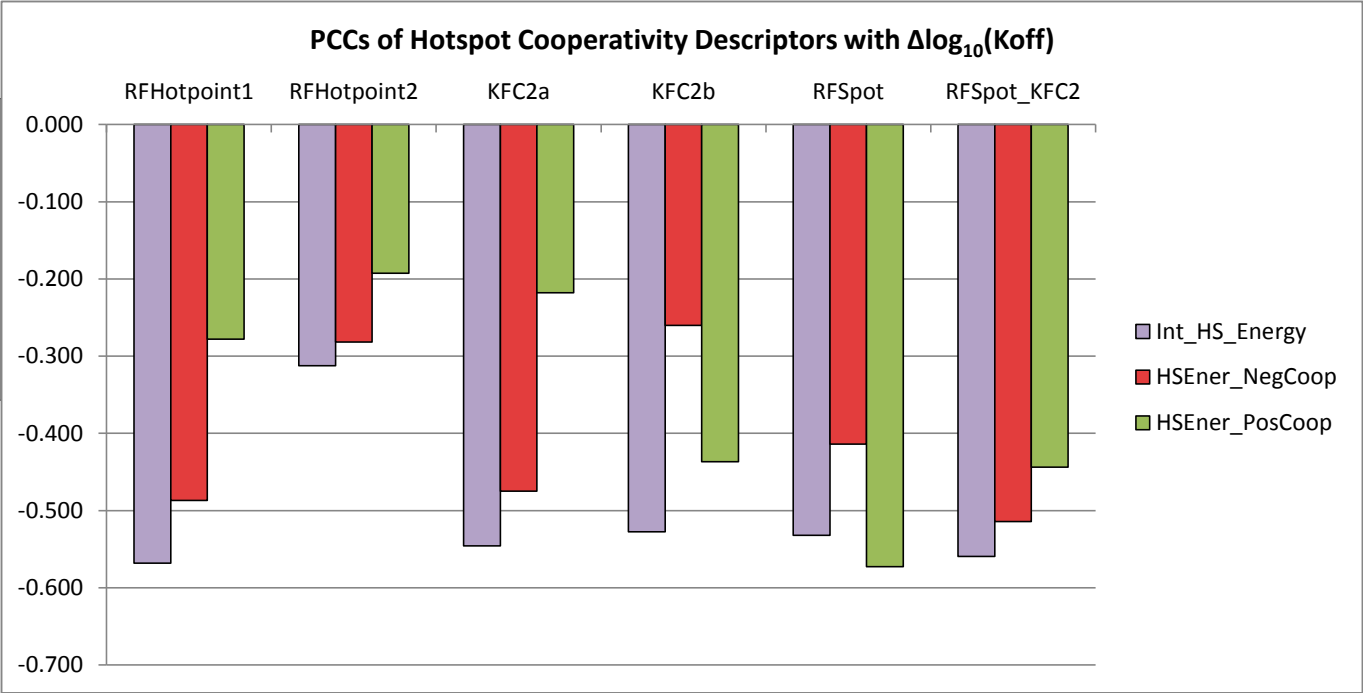

Supplement: Figure S2 — PCCs of hotspot cooperativity descriptors with Δlog10(koff). (PDF) [file pcbi.1003216.s005.pdf]
