# Supplementary material for: Characterizing Changes in the Rate of Protein-Protein Dissociation upon Interface Mutation Using Hotspot Energy and Organization
Source: PLoS Comput Biol. 2013 Sep 5;9(9):e1003216. doi: 10.1371/journal.pcbi.1003216 (PMC3764008; doi:10.1371/journal.pcbi.1003216)
Supplement: Text S1 — Molecular descriptors set. (PDF) [file pcbi.1003216.s011.pdf]

Supplementary Text S1: Molecular Descriptor Set

Molecular Descriptor	Description
DCOMPLEX	Atom Level Statistical Potential [1]
GEOMETRIC	Geometric packing potential function
OPUS_PSP1	Orientation-dependent Statistical All-atom Potential Derived from Side-chain Packing term 1 [2]
OPUS_PSP2	Orientation-dependent Statistical All-atom Potential Derived from Side-chain Packing term 2 [2]
OPUS_PSP3	Orientation-dependent Statistical All-atom Potential Derived from Side-chain Packing term 3 [2]
NIP	Normalized interface packing (NIP) [3]
NSc	Normalized Surface complementarity (NSc) score [3]
CHARM_total	CHARMM energy term [4]
CHARM_elec	CHARMM energy term [4]
CHARM_gb	CHARMM energy term [4]
CHARM_sasa	CHARMM energy term [4]
CHARM_gb+sasa	CHARMM energy term [4]
CHARM_vdwaals	CHARMM energy term [4]
ACE19_HYDRophobic	CHARMM19 force field ACE19_COUL - Hydrophobic Burial Term
ACE19_SELF	CHARMM19 force field ACE19_SELF - Electrostatic self-energy (Solvation)
ACE19_SCREENing	CHARMM19 force field ACE19_SCRE - Screening contribution (Solvation)
ACE19_COULomb	CHARMM19 force field ACE19_COUL - Hydrophobic Burial Term
ACE19_SOLVation	CHARMM19 force field ACE19_SOLV - sum of ACE19_SELF+ACE19_SCRE
ACE19_INTERaction	CHARMM19 force field ACE19_INTE - sum of ACE19_SCRE+ACE19_COUL
ACE19_SASL	CHARMM19 force field ACE19_SASL- SASA Solvation energy
PY_fa_atr	Lennard-jones attractive [5]
PY_fa_dun	Internal energy of side-chain rotamers as derived from Dunbrack's statistics based pair term [5]
PY_fa_pair	Favors salt bridges [5]
PY_fa_sol	Lazaridis-jarplus Solvation Energy[5]
PY_hbond_lr_bb	Backbone-backbone H-bonds distant in primary sequence[5]
PY_hbond_sr_bb	Backbone-backbone H-bonds close in primary sequence[5]
PY_fa_intra_rep	Lennard-jones repulsive between atoms in the same residue[5]
PY_fa_rep	Lennard-jones repulsive [5]
PY_hbond_bb_sc	H-bond energy sidechain-backbone[5]
PY_hbond_sc	H-bond energy sidechain-sidechain [5]
PY_pro_close	Proline ring closure energy[5]
FX1_int_BackHbond	Fold-X energy term [6]
FX1_int_SideHbond	Fold-X energy term [6]
FX1_int_Energy_VdW	Fold-X energy term [6]
FX1_int_Electro	Fold-X energy term [6]
FX1_int_Energy_SolvP	Fold-X energy term[6]
FX1_int_Energy_SolvH	Fold-X energy term[6]
FX1_int_Energy_vdwclash	Fold-X energy term[6]
FX1_int_energy_torsion	Fold-X energy term[6]
FX1_int_backbone_vdwclash	Fold-X energy term[6]
FX1_int_Entropy_sidec	Fold-X energy term[6]
FX1_int_Entropy_mainc	Fold-X energy term[6]
FX1_int_water bonds	Fold-X energy term[6]
FX1_int_ helix dipole	Fold-X energy term[6]
FX1_int_loop_entropy	Fold-X energy term[6]
FX1_int_cis_bond	Fold-X energy term[6]
FX1_int_disulfide	Fold-X energy term[6]
FX1_int_kn electrostatic	Fold-X energy term[6]
FX1_int_partial covalent interactions	Fold-X energy term[6]
FX1_int_Energy_Ionisation	Fold-X energy term[6]
FX1_int_Entropy Complex	Fold-X energy term[6]
FX2_BackHbond	Fold-X energy term[6]
FX2_SideHbond	Fold-X energy term[6]
FX2_Energy_VdW	Fold-X energy term[6]
FX2_Electro	Fold-X energy term[6]
FX2_Energy_SolvP	Fold-X energy term[6]
FX2_Energy_SolvH	Fold-X energy term[6]
FX2_Energy_vdwclash	Fold-X energy term[6]
FX2_energy_torsion	Fold-X energy term[6]
FX2_backbone_vdwclash	Fold-X energy term[6]
FX2_Entropy_sidec	Fold-X energy term[6]
FX2_Entropy_mainc	Fold-X energy term[6]
FX2_water bonds	Fold-X energy term[6]
FX2_ helix dipole	Fold-X energy term[6]
FX2_loop_entropy	Fold-X energy term[6]
FX2_cis_bond	Fold-X energy term[6]
FX2_disulfide	Fold-X energy term[6]
FX2_kn electrostatic	Fold-X energy term[6]
FX2_partial covalent interactions	Fold-X energy term[6]
FX2_Energy_Ionisation	Fold-X energy term[6]
FX2_Entropy Complex	Fold-X energy term[6]
DFIRE	Atom Based Statistical Potential [7]
CP_BFKV	Residue Level Statistical Potential [8]
CP_BL	Residue Level Statistical Potential [9]

CP_BT	Residue Level Statistical Potential [10]
CP_GKS	Residue Level Statistical Potential [11]
CP_HLPL	Residue Level Statistical Potential [12]
CP_MJPL	Residue Level Statistical Potential [12]
CP_MJ3h	Residue Level Statistical Potential [13]
CP_MJ2h	Residue Level Statistical Potential [14]
CP_MJ1	Residue Level Statistical Potential [15]
CP_MJ2	Residue Level Statistical Potential [14]
CP_MSBM	Residue Level Statistical Potential [16]
CP_MS	Residue Level Statistical Potential [17]
CP_Qa	Residue Level Statistical Potential [18]
CP_Qm	Residue Level Statistical Potential [18]
CP_Qp	Residue Level Statistical Potential [18]
CP_RO	Residue Level Statistical Potential [19]
CP_SKOb	Residue Level Statistical Potential [20]
CP_SKOa	Residue Level Statistical Potential [20]
CP_SJKG	Residue Level Statistical Potential [21]
CP_TD	Residue Level Statistical Potential [22]
CP_TEl	Residue Level Statistical Potential [23]
CP_TEs	Residue Level Statistical Potential [23]
CP_TS	Residue Level Statistical Potential [24]
CP_VD	Residue Level Statistical Potential
AP_DARS	Statistical Potential [25]
AP_URS	Statistical Potential [25]
AP_MPS	Statistical Potential [25]
DDFIRE	dDFIRE Statistical Potential [26]
AP_DOPE	Standard Dope Potential [27]
AP_DOPE_HR	High-Resolution Dope Potential [27]
CP_RMFCEN1	6 bin distance-dependent centroid-centroid potential [28]
CP_RMFCEN2	7 bin distance-dependent centroid-centroid potential [28]
CP_RMFCA	Alpha-Carbon Potential [29]
CP_SKOIP	Statistical Intermolecular Contact Potential [30]
CP_TB	Docking Contact Potential [31]
CP_TSC	2-Bin Docking Potential [32]
AP_T1	Two-step docking atomistic potential [32]
AP_T2	Two-step docking atomistic potential [32]

References

1. Liu, S., et al., *A physical reference state unifies the structure-derived potential of mean force for protein folding and binding.* Proteins, 2004. **56**(1): p. 93-101.
2. Lu, M., A.D. Dousis, and J. Ma, *OPUS-PSP: an orientation-dependent statistical all-atom potential derived from side-chain packing.* J Mol Biol, 2008. **376**(1): p. 288-301.
3. Mitra, P. and D. Pal, *New measures for estimating surface complementarity and packing at protein-protein interfaces.* FEBS Lett, 2010. **584**(6): p. 1163-8.
4. Brooks, B.R., et al., *CHARMM: the biomolecular simulation program.* J Comput Chem, 2009. **30**(10): p. 1545-614.
5. Chaudhury, S., S. Lyskov, and J.J. Gray, *PyRosetta: a script-based interface for implementing molecular modeling algorithms using Rosetta.* Bioinformatics, 2010. **26**(5): p. 689-91.
6. Schymkowitz, J., et al., *The FoldX web server: an online force field.* Nucleic Acids Res, 2005. **33**(Web Server issue): p. W382-8.
7. Zhang, C., et al., *The dependence of all-atom statistical potentials on structural training database.* Biophys J, 2004. **86**(6): p. 3349-58.
8. Bastolla, U., et al., *How to guarantee optimal stability for most representative structures in the Protein Data Bank.* Proteins, 2001. **44**(2): p. 79-96.
9. Bryant, S.H. and C.E. Lawrence, *An empirical energy function for threading protein sequence through the folding motif.* Proteins, 1993. **16**(1): p. 92-112.

10. Betancourt, M.R. and D. Thirumalai, *Pair potentials for protein folding: choice of reference states and sensitivity of predicted native states to variations in the interaction schemes.* Protein Sci, 1999. **8**(2): p. 361-9.
11. Godzik, A., A. Kolinski, and J. Skolnick, *Are proteins ideal mixtures of amino acids? Analysis of energy parameter sets.* Protein Sci, 1995. **4**(10): p. 2107-17.
12. Park, B. and M. Levitt, *Energy functions that discriminate X-ray and near native folds from well-constructed decoys.* J Mol Biol, 1996. **258**(2): p. 367-92.
13. Miyazawa, S. and R.L. Jernigan, *Self-consistent estimation of inter-residue protein contact energies based on an equilibrium mixture approximation of residues.* Proteins, 1999. **34**(1): p. 49-68.
14. Miyazawa, S. and R.L. Jernigan, *Residue-residue potentials with a favorable contact pair term and an unfavorable high packing density term, for simulation and threading.* J Mol Biol, 1996. **256**(3): p. 623-44.
15. Miyazawa, S. and R.L. Jernigan, *Estimation of effective interresidue contact energies from protein crystal structures: quasi-chemical approximation.* Macromolecules, 1985. **18**(3): p. 534-552.
16. Simons, K.T., et al., *Assembly of protein tertiary structures from fragments with similar local sequences using simulated annealing and Bayesian scoring functions.* J Mol Biol, 1997. **268**(1): p. 209-25.
17. Mirny, L.A. and E.I. Shakhnovich, *How to derive a protein folding potential? A new approach to an old problem.* J Mol Biol, 1996. **264**(5): p. 1164-79.
18. Boniecki, M., et al., *Protein fragment reconstruction using various modeling techniques.* J Comput Aided Mol Des, 2003. **17**(11): p. 725-38.
19. Robson, B. and D.J. Osguthorpe, *Refined models for computer simulation of protein folding. Applications to the study of conserved secondary structure and flexible hinge points during the folding of pancreatic trypsin inhibitor.* J Mol Biol, 1979. **132**(1): p. 19-51.
20. Skolnick, J., A. Kolinski, and A. Ortiz, *Derivation of protein-specific pair potentials based on weak sequence fragment similarity.* Proteins, 2000. **38**(1): p. 3-16.
21. Skolnick, J., et al., *Derivation and testing of pair potentials for protein folding. When is the quasichemical approximation correct?* Protein Sci, 1997. **6**(3): p. 676-88.
22. Thomas, P.D. and K.A. Dill, *An iterative method for extracting energy-like quantities from protein structures.* Proc Natl Acad Sci U S A, 1996. **93**(21): p. 11628-33.
23. Tobi, D., et al., *On the design and analysis of protein folding potentials.* Proteins, 2000. **40**(1): p. 71-85.
24. Tanaka, S. and H.A. Scheraga, *Medium- and long-range interaction parameters between amino acids for predicting three-dimensional structures of proteins.* Macromolecules, 1976. **9**(6): p. 945-50.
25. Chuang, G.Y., et al., *DARS (Decoys As the Reference State) potentials for protein-protein docking.* Biophys J, 2008. **95**(9): p. 4217-27.
26. Yang, Y. and Y. Zhou, *Specific interactions for ab initio folding of protein terminal regions with secondary structures.* Proteins, 2008. **72**(2): p. 793-803.
27. Shen, M.Y. and A. Sali, *Statistical potential for assessment and prediction of protein structures.* Protein Sci, 2006. **15**(11): p. 2507-24.
28. Rajgaria, R., S.R. McAllister, and C.A. Floudas, *Distance dependent centroid to centroid force fields using high resolution decoys.* Proteins, 2008. **70**(3): p. 950-70.
29. Rajgaria, R., S.R. McAllister, and C.A. Floudas, *A novel high resolution Calpha--Calpha distance dependent force field based on a high quality decoy set.* Proteins, 2006. **65**(3): p. 726-41.
30. Lu, H., L. Lu, and J. Skolnick, *Development of unified statistical potentials describing protein-protein interactions.* Biophys J, 2003. **84**(3): p. 1895-901.

31. Tobi, D. and I. Bahar, *Optimal design of protein docking potentials: efficiency and limitations.* Proteins, 2006. **62**(4): p. 970-81.
32. Tobi, D., *Designing coarse grained-and atom based-potentials for protein-protein docking.* BMC Struct Biol, 2010. **10**: p. 40.
