# Supplementary material for: Characterizing Changes in the Rate of Protein-Protein Dissociation upon Interface Mutation Using Hotspot Energy and Organization
Source: PLoS Comput Biol. 2013 Sep 5;9(9):e1003216. doi: 10.1371/journal.pcbi.1003216 (PMC3764008; doi:10.1371/journal.pcbi.1003216)
Supplement: Text S2 — Hotspot descriptor consistencies and biases. (PDF) [file pcbi.1003216.s012.pdf]

Hotspot Descriptors: Consistencies and Biases

Here we investigate consistencies in correlations achieved by the hotspot descriptors with $\Delta log_{10}(Koff)$ as generated from different hotspot predictor algorithms and presented in Table 1 in the main text. Each of the *Int_HS_Energy* descriptors generated by all 6 hotspot predictors, apart from *RFHotpoint2*, show a correlation of $0.53 <= |R| <= 0.57$ with $\Delta log_{10}(Koff)$. Though each hotspot predictor algorithm is developed using diverse features and each with their own biases, the correlation shown by *Int_HS_Energy* is consistent throughout. Analysis of the remaining hotspot descriptors show that, whereas the change in total interface hotspot energy (*Int_HS_Energy*) is consistent in correlation for the different hotspot prediction algorithms with a low variance of σ=0.01, when looking at mutants in particular regions of the interface, such as *CoreHSEnergy*/*RimHSEnergy*/*SuppHSEnergy*, variances in correlations increase (*σ=0.10, σ=0.19, σ=0.19* respectively). The higher variance in these regions is expected and more than probably emanates from the biases present in the features and datasets used in the original hotspot predictions models. For example KFC2 models are dominated by solvent accessibility features [1] which biases hotspot predictions towards the core. This bias is further emphasized also because few rim hotspot mutations are available to train on as in *ASEdB* [2]. This results in poor correlations for $RimHSEnergy_{KFC2a}$ and $RimHSEnergy_{KFC2b}$ (*R=-0.01, R=0* respectively) as a minimal number of hotspots are detected in these regions. Higher correlations are observed for the support regions with *R=-0.62*, *R=-0.49* for $SuppHSEnergy_{KFC2a}$ and $SuppHSEnergy_{KFC2a}$ respectively. *RFSpot* and *RFSpot_KFC2* show the most consistent correlations for the three regions, with (*R=-0.54*, *R=-0.56*), for $CoreHSEnergy_{RFSpot}$, and $CoreHSEnergy_{RFSpot\_KFC2}$, (*R=-0.3*, *R=-0.38*) for $RimHSEnergy_{RFSpot}$ and $RimHSEnergy_{RFSpot\_KFC2}$, and (*R=-0.38*, *R=-0.47*) for $SuppHSEnergy_{RFSpot}$, and $SuppHSEnergy_{RFSpot\_KFC2}$. *RFHotpoint1* on the other hand shows the lowest correlation for the support region and a highest for the core region whereas *RFHotpoint2*, shows poor correlations for the three regions. The low correlations for RF*Hotpoint2* are expected given it is the poorest hotspot predictor of all 6 with an MCC of 0.218 and a high False-Positive-Rate of 0.277 for hotspot prediction (See Supplementary Text S4). For the cooperativity descriptors, variances are also higher to that of the *Int_HS_Energy* descriptors; σ=0.11, and *σ=0.15* for *HSEnerg_NegCoop* and *HSEner_PosCoop* respectively. Unlike *Int_HS_Energy* which only depends on the number of hotspots and their energies, these descriptors are a function of the number and size of hotregions in the complex. Therefore biases of hotspot predictors towards certain interface regions may result in larger hotregions or joining of two hotregions, which in turn directly affects the values of these descriptors.

References

1. Zhu, X. and J.C. Mitchell, *KFC2: a knowledge-based hot spot prediction method based on interface solvation, atomic density, and plasticity features.* Proteins, 2011. **79**(9): p. 2671-83.
2. Tong, W., L. Li, and Z. Weng, *Computational prediction of binding hotspots.* Conf Proc IEEE Eng Med Biol Soc, 2004. **4**: p. 2980-3.
