# Supplementary material for: Characterizing Changes in the Rate of Protein-Protein Dissociation upon Interface Mutation Using Hotspot Energy and Organization
Source: PLoS Comput Biol. 2013 Sep 5;9(9):e1003216. doi: 10.1371/journal.pcbi.1003216 (PMC3764008; doi:10.1371/journal.pcbi.1003216)
Supplement: Text S3 — Effects of cross validation routine. (PDF) [file pcbi.1003216.s013.pdf]

Effects of Cross-Validation Routine

The results presented in the main text which use 20-fold cross validation for the generation of test predictions, are an estimate of predictive power given one already has some mutant information on the complex in question to train upon. This type of cross validation is not a valid estimate of the generalization ability on an unseen complex. Therefore, two additional cross-validation mechanisms were also applied; Leave-Complex-Out CV (LCO-CV), where all mutations of a complex are left out as a test set and Leave-Homology-Out CV (LHO-CV); a more stringent form of cross-validation which accounts for homology and interface similarity as suggested in [1, 2]. The PCCs of the test predictions with ΔKoff, of the two CV routines, are shown in Figure S3.

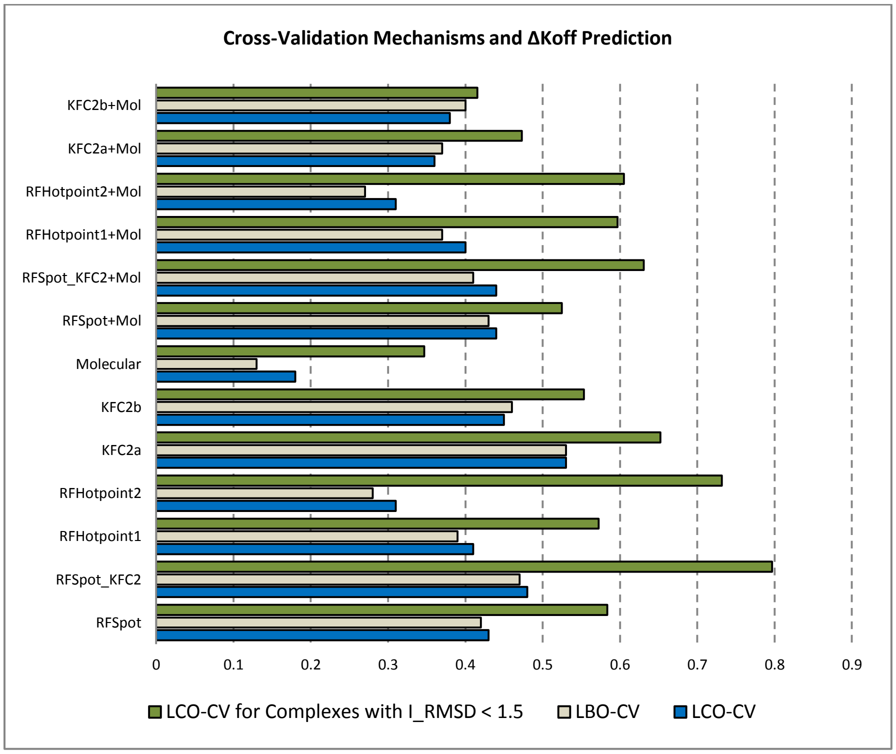

Figure S3. PCCs for Off-Rate Prediction Models with 713 off-rate mutant dataset from SKEMPI. Leave-Complex-Out CV(LCO-CV), Leave-Homolgy-Out CV (LHO-CV) and LCO-CV for complex which undergo minimal to no conformational changes with I_RMSD < 1.5 Å as defined in [3]. The models differ by their features sets. First 6 use hotspot descriptor sets, followed by molecular descriptor set model (Molecular), and models which combine both (+Mol).

Keeping in mind that 20-Fold CV results for models are at R > 0.7, the LCO-CV and LHO-CV, the models severely over-fit. In essence, the predictive ability of the hotspot descriptors such as $HSEner\_PosCoop_{RFSpot}$ (|R|=0.57), $Int\_HS\_Energy_{Hotpoint1}$ (|R|=0.57) and $SuppHSEnergy_{KFC2a}$ (|R|=0.62) is being impeded by the learning model and noise from other features. It is important to note that the LHO-CV might not be well suited for certain practical purposes. For example, if one wishes to be able to predict mutations on an enzyme inhibitor complex, it would be natural to have such complexes in the training set, unlike what is actually done here in LHO-CV. The largest amount of over-fitting is observed for the molecular descriptor model, which is alleviated with the hotspot descriptor models and In both CV mechanisms, the correlations achieved by the hotspot descriptor models, is higher than that achieved by the molecular descriptor set model.

To understand potential reasons behind the large reduction in prediction accuracies for LCO/LHO-CV mechanisms, LCO-CV was also performed on the subset of 14 complexes and 265 mutations which show little to no conformational change. It is observed that the reduction in our ability to model the effects of mutations on unseen/unrelated is largely affected by conformational changes. For example for $\text{RFSpot\_KFC2}_{\text{Off-Rate,}}$ the correlation achieved is as high as 0.8 when limited to rigid complexes, even though LCO-CV is being performed.

To further investigate potential differences in complexes across LHO folds, models are built for mutations only within a fold and the most important features are highlighted and compared to the features from models built on other LHO folds. Genetic Algorithm Feature Selection (GA-FS) is used to build such specialized off-rate prediction models and both linear and non-linear models are investigated. Table S9 summarizes the results of the GS-FS runs, the complexes within each set and the number of mutations per set. The PCCs with ΔKoff confirm that predictions on mutations where information from related complexes can be exploited may be highly accurate.

Table S9. Specialized Feature Selection Models for related sets of complexes. The PCCs shown are an average over the Leave-One-Out Cross Validation (LOO-CV) prediction results over 50 runs. LOO-CV results are an outer-wrapper validation loop, whereas an inner-wrapper validation loop is used to optimize the feature set and SVM Cost and gamma parameters.

SET_IDs	SET1	SET2	SET3	SET4	SET5	SET6	SET7	SET8	SET9	SET10	SET11
**GS-FS (SVM) PCC with ΔKoff**	0.55	0.89	0.66	0.97	0.94	0.91	0.84	0.75	0.71	0.92	0.68
**GS-FS (LR) PCC ΔKoff**	0.47	0.86	0.49	0.95	0.92	0.85	0.77	0.72	0.68	0.79	0.63
**Mutation Count**	58	62	79	39	74	87	63	36	84	100	31
**PDB_IDs**	1A22_A_B	1A4Y_A_B	1B2S_A_D	1CBW_FGH_I	1DAN_HL_UT	1EMV_A_B	1FC2_C_D	1IAR_A_B	1JRH_LH_I	1JTG_A_B	1KTZ_A_B
		1Z7X_W_X	1B2U_A_D	1GL0_E_I		1FR2_A_B	1LFD_A_B		1NMB_N_LH		1REW_AB_C
			1B3S_A_D	1GL1_A_I		2GYK_A_B	1MAH_A_F		2I26_N_L		2QJ9_AB_C
			1BRS_A_D	1TM1_E_I		2VLN_A_B	1MQ8_A_B		2VIR_AB_C		2QJA_AB_C
			1X1W_A_D	2FTL_E_I		2VLO_A_B	2AJF_A_E		2VIS_AB_C		2QJB_AB_C
			1X1X_A_D	2SIC_E_I		2VLP_A_B	2B42_A_B		2VLJ_ABC_DE		
						2VLQ_A_B	2GOX_A_B		2VLR_ABC_DE		
						2WPT_A_B	3D5R_A_C		3HFM_HL_Y		
							3D5S_A_C				
							3BP8_A_C				
							3BK3_A_C				

The Features that make it to the final models (Figure S4) indicate a heterogeneity set of features employed for each set of related complexes, and no one-feature-fits-all may be identified. This again may contribute to the reduction in PCCs when using LHO-CV mechanisms, as mutations on unseen complexes may be better predicted using features which were not prominent in the training set mutations. Biases related to different experimental methods from which the *ΔKoff* of the mutations where calculated are also known to have significant effects on the prediction of binding free energies [4, 5] and may also play a role in the reduction of accuracy when using LHO- and LCO-CV mechanisms

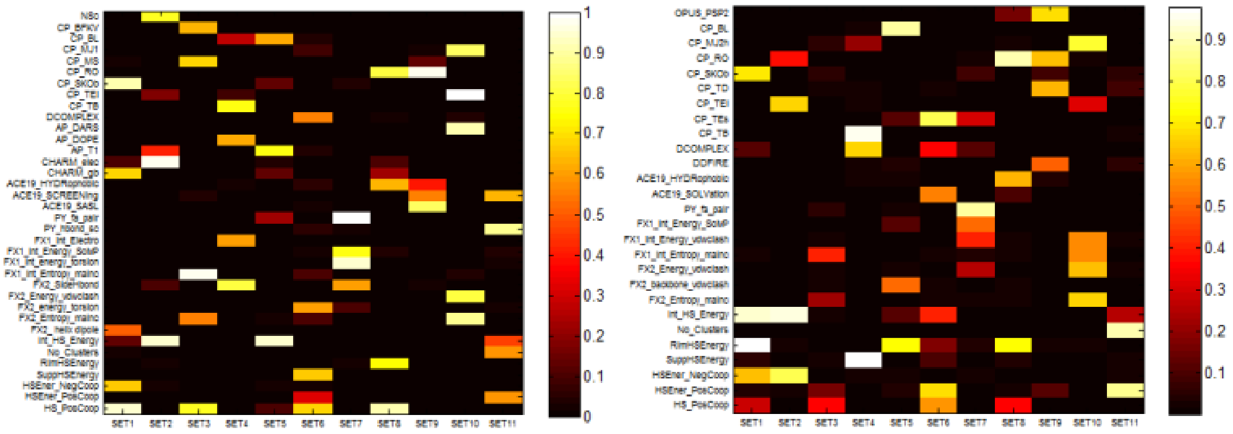

Figure S4 LEFT: GA-FS (LR), RIGHT: GA-FS (SVM). The colour bar indicates the percentage number of times the given feature made it to the feature set of the final model after a GA-FS run. Features shown are those which make it to the final model more than 50% of the time for at least one set on the x-axis.

References

1. Moal, I.H. and J. Fernandez-Recio, *SKEMPI: a Structural Kinetic and Energetic database of Mutant Protein Interactions and its use in empirical models.* Bioinformatics, 2012. **28**(20): p. 2600-7.
2. Kastritis, P.L. and A.M. Bonvin, *On the binding affinity of macromolecular interactions: daring to ask why proteins interact.* J R Soc Interface, 2013. **10**(79): p. 20120835.
3. Kastritis, P.L., et al., *A structure-based benchmark for protein-protein binding affinity.* Protein Sci, 2011. **20**(3): p. 482-91.
4. Kastritis, P.L. and A.M. Bonvin, *Are scoring functions in protein-protein docking ready to predict interactomes? Clues from a novel binding affinity benchmark.* J Proteome Res, 2010. **9**(5): p. 2216-25.
5. Moal, I.H., R. Agius, and P.A. Bates, *Protein-protein binding affinity prediction on a diverse set of structures.* Bioinformatics, 2011. **27**(21): p. 3002-9.
