# Supplementary material for: Characterizing Changes in the Rate of Protein-Protein Dissociation upon Interface Mutation Using Hotspot Energy and Organization
Source: PLoS Comput Biol. 2013 Sep 5;9(9):e1003216. doi: 10.1371/journal.pcbi.1003216 (PMC3764008; doi:10.1371/journal.pcbi.1003216)
Supplement: Text S4 — Hotspot predictor predictions and performance. (PDF) [file pcbi.1003216.s014.pdf]

Supplementary Text S4: Hotspot Predictor Predictions and Performance

All hotspot prediction algorithms and performance measures in this section are related to how well the hotspot predictors are able to detect ΔΔGs of single-point alanine mutations which satisfy ΔΔG > 2kcal/mol i.e. the prediction of hotspots. Though *RFSpot*, *RFSpot_KFC2*, *KF2a*, *KFC2b*, *RFHotpoint1* and *RFHotpoint2* are subsequently used for the generation of hotspot descriptors which are then used for the prediction of off-rate mutations, this supplementary section does not show any predictions or performance analysis for off-rate mutations. Table S10 summarizes each hotspot predictors analyzed. Table S16 shows the classification performance of each hotspot predictor on SKEMPI Alanine ΔΔG Data. Note however that performance values for each predictor are not comparable as each predictor's performance is calculated on different subsets of mutations according to what was available or presented in their original works. Performance comparison on intersections of mutations, are however shown in Table S13 for *RFSpot* vs. other hotspot predictors (Table S12) and Table S14 for *RFspot_KFC2* vs. other hotspot predictors (Table S12). Table S15 shows the comparison between server predictions from *Hotpoint* with that of *RFhotpoint1* and *RFHotpoint2* on their intersection of predictions. Table S16 shows the list of predictions of every hotspot predictor considered on SKEMPI alanine data, for which predictions where available in the original work and for predictions on mutations which were not used in the model training in the case of servers (i.e *KFC2a*, *KFC2b* and *Hotpoint*).

Table S10. Hotspot Predictors Analyzed in this section.

Hotpot Predictor	Description and Source of Predictions
**RFSpot**	**RFSpot***: Random Forest Model trained on SKEMPI alanine data, with molecular features.<br>**RFSpot_KFC2***: Random Forest Model trained on SKEMPI alanine data, with molecular features and features from KFC2a and KFC2b for which KFC2a and KFC2b Features from KFC2 server where available. Random Forest threshold adjusted to achieve same FPR of RFSpot for comparison.
**KFC2 [1]**	**KFC2a_Orig**: uses predictions on ASEdB from [1]<br>**KFC2b_Orig**: uses predictions on ASEdB from [1]<br>**KFC2a ***: uses server predictions on SKEMPI alanine data which is not in ASEdB from KFC2 Server<br>**KFC2b***: uses server predictions on SKEMPI alanine data which is not in ASEdB from KFC2 Server
**Robetta [2]**	**Robetta**: uses predictions on ASEdB from [2]
**Hotpoint [3]**	**Hotpoint_Orig**: uses predictions on ASEdB from [3]<br>**Hotpoint** uses server prediction on SKEMPI alanine data which is not in ASEdB from Hotpoint Prediction Server<br>**RFHotpoint1***: Random Forest Model trained on SKEMPI alanine data, with original Hotpoint Features for which Hotpoint Features from Hotpoint server where available<br>**RFHotpoint2***: Random Forest Model trained on SKEMPI alanine data , with Hotpoint Features for which Hotpoint Features from server where available. Random Forest Threshold lowered to allow for more TPs
**RFMirror [4]**	**RFMirror**: uses predictions on ASEdB from [4]
**SVM score [5]**	**SVM score**: uses predictions on ASEdB from [5]
**TSVM score [5]**	**TSVM score**: uses predictions on ASEdB from [5]

** Indicate Hotspot Predictors used for the generation of hotspot descriptors and further analyzed in main manuscript for off-rate prediction.*

Table S11. Performance of Hotspot Predictors on SKEMPI.
Performance of Hotspot Predictors on intersection of original data used in original hotspot predictors and SKEMPI. Predictors are ranked according to MCC.

Predictor	Intersection with SKEMPI	TPR	FPR	MCC	F1	Acc	Spec	Prec
TSVM score	TSVM score	0.673	0.136	0.508	0.619	0.823	0.864	0.574
RFSpot_KFC2*	RFSpot_KFC2*	0.490	0.083	0.452	0.560	0.814	0.917	0.652
SVM score	SVM score	0.615	0.152	0.438	0.566	0.798	0.848	0.525
RFMirror	RFMirror	0.500	0.094	0.434	0.545	0.816	0.906	0.600
KFC2a*	KFC2a*	0.734	0.279	0.402	0.568	0.724	0.721	0.463
KFC2b	KFC2b	0.452	0.103	0.383	0.509	0.789	0.897	0.583
Hotpoint_Orig	Hotpoint_Orig	0.552	0.196	0.368	0.593	0.707	0.804	0.640
KFC2b*	KFC2b*	0.436	0.129	0.328	0.477	0.764	0.871	0.526
Robetta	Robetta	0.458	0.155	0.295	0.440	0.769	0.845	0.423
RFSpot*	RFSpot*	0.268	0.083	0.237	0.350	0.761	0.917	0.506
RFHotpoint1*	RFHotpoint1*	0.319	0.125	0.229	0.395	0.711	0.875	0.517
RFHotpoint2*	RFHotpoint2*	0.504	0.277	0.218	0.466	0.658	0.723	0.433
KFC2a_Orig	KFC2a_Orig	0.258	0.124	0.159	0.314	0.727	0.876	0.400
Hotpoint	Hotpoint	0.500	0.379	0.113	0.424	0.584	0.621	0.368

** Indicate Hotspot Predictors used for the generation of hotspot descriptors and further analyzed in main manuscript for off-rate prediction.*

Supplementary Text S4: Hotspot Predictor Predictions and Performance

Table S12. Performance of Hotspot Predictors on SKEMPI.

Performance of Hotspot Predictors on intersection of original data used in original hotspot predictors and SKEMPI. Positioned for comparison with Table S13 and Table S14.

Predictor	Intersection with SKEMPI	TPR	FPR	MCC	F1	Acc	Spec	Prec
**RFSpot_KFC2***	**RFSpot_KFC2***	0.490	0.083	0.452	0.560	0.814	0.917	0.652
**RFSpot***	**RFSpot***	0.268	0.083	0.237	0.350	0.761	0.917	0.506
**KFC2a***	**KFC2a***	0.734	0.279	0.402	0.568	0.724	0.721	0.463
**KFC2b***	**KFC2b***	0.436	0.129	0.328	0.477	0.764	0.871	0.526
**Hotpoint**	**Hotpoint**	0.500	0.379	0.113	0.424	0.584	0.621	0.368
**RFHotpoint1***	**RFHotpoint1***	0.319	0.125	0.229	0.395	0.711	0.875	0.517
**RFHotpoint2***	**RFHotpoint2***	0.504	0.277	0.218	0.466	0.658	0.723	0.433
**KFC2a_Orig**	**KFC2a_Orig**	0.258	0.124	0.159	0.314	0.727	0.876	0.400
**KFC2b_Orig**	**KFC2b_Orig**	0.452	0.103	0.383	0.509	0.789	0.897	0.583
**Robetta**	**Robetta**	0.458	0.155	0.295	0.440	0.769	0.845	0.423
**Hotpoint_Orig**	**Hotpoint_Orig**	0.552	0.196	0.368	0.593	0.707	0.804	0.640
**RFMirror**	**RFMirror**	0.500	0.094	0.434	0.545	0.816	0.906	0.600
**SVM score**	**SVM score**	0.615	0.152	0.438	0.566	0.798	0.848	0.525
**TSVM score**	**TSVM score**	0.673	0.136	0.508	0.619	0.823	0.864	0.574

** Indicate Hotspot Predictors used for the generation of hotspot descriptors and further analyzed in main manuscript for off-rate prediction.*

Table S13. RFSpot and Hotspot Predictors.

Performance of RFSpot on intersection of original data used in original hotspot predictors and SKEMPI. Highlighted in Blue are instances where RFSpot performs better than respective hotspot predictor, as compared with values from Table S12.

Predictor	Intersection with SKEMPI	TPR	FPR	MCC	F1	Acc	Spec	Prec
**RFSpot**	**RFSpot_KFC2***	0.268	0.083	0.237	0.350	0.761	0.917	0.506
**RFSpot**	**RFSpot***	0.268	0.083	0.237	0.350	0.761	0.917	0.506
**RFSpot**	**KFC2a***	0.255	0.080	0.230	0.340	0.756	0.920	0.511
**RFSpot**	**KFC2b***	0.255	0.080	0.230	0.340	0.756	0.920	0.511
**RFSpot**	**Hotpoint**	0.279	0.118	0.199	0.361	0.698	0.882	0.511
**RFSpot**	**RFHotpoint1***	0.291	0.110	0.223	0.374	0.713	0.890	0.526
**RFSpot**	**RFHotpoint2***	0.291	0.110	0.223	0.374	0.713	0.890	0.526
**RFSpot**	**KFC2a_Orig**	0.323	0.072	0.316	0.417	0.781	0.928	0.588
**RFSpot**	**KFC2b_Orig**	0.323	0.072	0.316	0.417	0.781	0.928	0.588
**RFSpot**	**Robetta**	0.417	0.062	0.418	0.500	0.835	0.938	0.625
**RFSpot**	**Hotpoint_Orig**	0.310	0.087	0.287	0.429	0.680	0.913	0.692
**RFSpot**	**RFMirror**	0.296	0.079	0.272	0.376	0.784	0.921	0.516
**RFSpot**	**SVM score**	0.269	0.073	0.252	0.350	0.786	0.927	0.500
**RFSpot**	**TSVM score**	0.269	0.073	0.252	0.350	0.786	0.927	0.500

** Indicate Hotspot Predictors used for the generation of hotspot descriptors and further analyzed in main manuscript for off-rate prediction.*

Table S14. RFSpot_KFC2 and Hotspot Predictors.

Performance of RFSpot_KFC2 on intersection of original data used in original hotspot predictors and SKEMPI. Highlighted in Blue are instances where RFSpot_KFC2 performs better than respective hotspot predictor, as compared with values from Table S12.

Predictor	Intersection with SKEMPI	TPR	FPR	MCC	F1	Acc	Spec	Prec
**RFSpot_KFC2**	**RFSpot_KFC2***	0.490	0.083	0.452	0.560	0.814	0.917	0.652
**RFSpot_KFC2**	**RFSpot***	0.490	0.083	0.452	0.560	0.814	0.917	0.652
**RFSpot_KFC2**	**KFC2a***	0.500	0.098	0.436	0.556	0.803	0.902	0.627
**RFSpot_KFC2**	**KFC2b***	0.500	0.098	0.436	0.556	0.803	0.902	0.627
**RFSpot_KFC2**	**Hotpoint**	0.535	0.133	0.424	0.582	0.765	0.867	0.639
**RFSpot_KFC2**	**RFHotpoint1***	0.511	0.113	0.431	0.574	0.776	0.887	0.655
**RFSpot_KFC2**	**RFHotpoint2***	0.511	0.113	0.431	0.574	0.776	0.887	0.655
**RFSpot_KFC2**	**KFC2a_Orig**	0.452	0.082	0.419	0.528	0.805	0.918	0.636
**RFSpot_KFC2**	**KFC2b_Orig**	0.452	0.082	0.419	0.528	0.805	0.918	0.636
**RFSpot_KFC2**	**Robetta**	0.583	0.052	0.583	0.651	0.876	0.948	0.737
**RFSpot_KFC2**	**Hotpoint_Orig**	0.483	0.087	0.451	0.596	0.747	0.913	0.778
**RFSpot_KFC2**	**RFMirror**	0.500	0.063	0.495	0.581	0.841	0.937	0.692
**RFSpot_KFC2**	**SVM score**	0.519	0.068	0.499	0.587	0.844	0.932	0.675
**RFSpot_KFC2**	**TSVM score**	0.519	0.068	0.499	0.587	0.844	0.932	0.675

** Indicate Hotspot Predictors used for the generation of hotspot descriptors and further analyzed in main manuscript for off-rate prediction.*

Table S15. Performance comparison of RFHotpoint1 and RFHotpoint2 with server prediction of Hotpoint on SKEMP.

Hotpot Predictor	TPR	FPR	MCC	F1	Acc	Spec	Prec
Hotpoint	0.500	0.379	0.113	0.424	0.584	0.621	0.368
RFHotpoint1	0.360	0.128	0.268	0.437	0.715	0.872	0.554
RFHotpoint2	0.570	0.303	0.253	0.505	0.658	0.697	0.454

Supplementary Text S4: Hotspot Predictor Predictions and Performance

Table S16. Hotpot predictor predictions on intersection with SKEMPI. 20-Fold CV results presented for hotspot predictors developed in this work: RFSpot, RFSpot_KFC2, RFHotpoint1 and RFHotpoint2. Source of predictions and description of hotspot predictors listed in Table S10. Performance results for predictions listed in TableS11-S14. 1: Hotspot, -1:Non-Hotspot, -: Prediction not available.

PDB_MUT ID	Truth	DDG	RFSpot_KFC2*	RFSpot*	KFC2*	KFC2*	Hotpoint	RFHotpoint1*	RFHotpoint2*	KFC2a_Orig	KFC2b_Orig	Robetta	Hotpoint_Orig	RFMirror	SVM score	TSVM score
1A22.pdb_AC182A	-1	1.010	-1	-1	-1	-1	1	-1	-1	--	--	--	--	--	--	--
1A22.pdb_AD171A	-1	0.791	-1	-1	--	--	--	-1	-1	--	--	1	--	1	1	1
1A22.pdb_AE174A	-1	-0.925	-1	-1	--	--	--	--	--	--	--	-1	--	-1	-1	-1
1A22.pdb_AE56A	-1	0.411	-1	-1	--	--	--	-1	-1	--	--	-1	--	-1	-1	-1
1A22.pdb_AE65A	-1	-0.473	-1	-1	--	--	--	--	--	--	--	-1	--	-1	-1	-1
1A22.pdb_AF176A	-1	0.411	-1	-1	--	--	--	--	--	--	--	-1	--	1	-1	-1
1A22.pdb_AF191A	-1	0.191	-1	-1	-1	-1	--	--	--	--	--	--	--	--	--	--
1A22.pdb_AF25A	-1	-0.447	-1	-1	--	--	--	--	--	--	--	-1	--	-1	-1	-1
1A22.pdb_AH18A	-1	-0.486	-1	-1	--	--	--	-1	-1	--	--	-1	--	-1	-1	-1
1A22.pdb_AH21A	-1	0.155	1	-1	--	--	--	1	1	--	--	-1	--	-1	-1	-1
1A22.pdb_AI179A	-1	0.806	-1	-1	--	--	--	1	1	--	--	-1	--	-1	-1	-1
1A22.pdb_AK168A	-1	-0.155	-1	-1	--	--	--	1	1	--	--	1	--	1	-1	-1
1A22.pdb_AK172A	1	2.015	1	-1	--	--	--	-1	-1	--	--	-1	--	-1	-1	-1
1A22.pdb_AL45A	-1	1.225	-1	-1	--	--	--	1	1	--	--	-1	--	1	-1	-1
1A22.pdb_AN63A	-1	0.314	-1	-1	--	--	--	--	--	--	--	-1	--	1	-1	-1
1A22.pdb_AP48A	-1	0.411	-1	-1	-1	-1	-1	-1	-1	--	--	--	--	--	--	--
1A22.pdb_AP61A	-1	1.209	-1	-1	-1	-1	--	--	--	--	--	--	--	--	--	--
1A22.pdb_AQ22A	-1	-0.220	-1	-1	--	--	--	-1	-1	--	--	-1	--	-1	-1	-1
1A22.pdb_AQ46A	-1	0.108	-1	-1	--	--	--	-1	-1	--	--	-1	--	-1	-1	-1
1A22.pdb_AQ68A	-1	0.588	-1	-1	--	--	--	-1	-1	--	--	-1	--	-1	-1	-1
1A22.pdb_AR167A	-1	0.278	-1	-1	--	--	--	1	1	--	--	-1	--	-1	-1	-1
1A22.pdb_AR178A	1	2.426	1	-1	--	--	--	-1	-1	--	--	-1	--	-1	-1	-1
1A22.pdb_AR183A	-1	0.543	-1	-1	--	--	--	--	--	--	--	-1	--	-1	--	--
1A22.pdb_AR64A	-1	1.643	1	1	--	--	--	-1	-1	--	--	1	--	-1	1	1
1A22.pdb_AS51A	-1	0.348	-1	-1	--	--	--	--	--	--	--	-1	--	1	-1	-1
1A22.pdb_AS62A	-1	0.155	-1	-1	--	--	--	1	1	--	--	-1	--	-1	-1	-1
1A22.pdb_AT175A	-1	1.907	-1	-1	--	--	--	-1	1	--	--	1	--	1	1	-1
1A22.pdb_AY164A	-1	0.348	-1	-1	--	--	--	-1	-1	--	--	-1	--	-1	-1	-1
1A22.pdb_AY42A	-1	0.199	-1	-1	--	--	--	-1	-1	--	--	1	--	-1	-1	-1
1A22.pdb_BC308A	-1	0.000	-1	-1	-1	-1	1	1	1	--	--	--	--	--	--	--
1A22.pdb_BC322A	-1	0.000	-1	-1	1	-1	1	-1	1	--	--	--	--	--	--	--
1A22.pdb_BD326A	-1	0.994	-1	-1	--	--	--	--	--	--	--	--	--	-1	-1	-1
1A22.pdb_BD364A	-1	1.487	-1	-1	--	--	--	-1	-1	--	--	--	--	-1	-1	1
1A22.pdb_BE244A	-1	1.693	-1	-1	--	--	--	-1	-1	--	--	--	--	-1	-1	-1
1A22.pdb_BE275A	-1	-0.094	-1	-1	--	--	--	-1	-1	--	--	--	--	-1	-1	-1
1A22.pdb_BE320A	-1	-0.182	-1	-1	--	--	--	-1	-1	--	--	--	--	-1	-1	-1
1A22.pdb_BE327A	-1	0.971	-1	-1	--	--	--	-1	1	--	--	--	--	-1	-1	-1
1A22.pdb_BI303A	-1	1.608	-1	-1	--	--	--	-1	-1	--	--	--	--	-1	-1	-1
1A22.pdb_BI305A	-1	1.942	-1	-1	--	--	--	--	--	--	--	--	--	-1	-1	-1
1A22.pdb_BI365A	1	2.131	-1	-1	--	--	--	-1	-1	--	--	--	--	-1	-1	-1
1A22.pdb_BK310A	-1	0.043	-1	-1	-1	-1	--	--	--	--	--	--	--	--	--	--
1A22.pdb_BK321A	-1	0.081	-1	-1	--	--	--	--	--	--	--	--	--	-1	-1	-1
1A22.pdb_BK367A	-1	-0.018	-1	-1	--	--	--	-1	-1	--	--	--	--	-1	-1	-1
1A22.pdb_BK415A	-1	0.785	-1	-1	-1	-1	--	--	--	--	--	--	--	--	--	--
1A22.pdb_BN418A	-1	0.296	-1	-1	--	--	--	1	1	--	--	--	--	-1	1	-1
1A22.pdb_BP306A	1	3.306	-1	-1	1	-1	-1	1	1	--	--	--	--	--	--	--
1A22.pdb_BQ274A	-1	0.000	-1	-1	--	--	--	-1	-1	--	--	--	--	-1	-1	-1
1A22.pdb_BQ366A	-1	0.017	-1	-1	--	--	--	-1	-1	--	--	--	--	-1	-1	-1
1A22.pdb_BQ416A	-1	0.891	-1	-1	--	--	--	--	--	--	--	--	--	-1	--	--
1A22.pdb_BR243A	1	2.116	1	-1	--	--	--	-1	-1	--	--	--	--	1	1	1
1A22.pdb_BR271A	-1	0.536	-1	-1	--	--	--	-1	1	--	--	--	--	1	-1	-1
1A22.pdb_BR417A	-1	0.274	-1	-1	--	--	--	-1	-1	--	--	--	--	-1	-1	-1
1A22.pdb_BS298A	-1	-0.055	-1	-1	--	--	--	--	--	--	--	--	--	-1	-1	-1
1A22.pdb_BS302A	-1	-0.182	-1	-1	--	--	--	--	--	--	--	--	--	-1	-1	-1
1A22.pdb_BS324A	-1	0.274	-1	-1	--	--	--	--	--	--	--	--	--	-1	-1	-1
1A22.pdb_BS419A	-1	0.034	-1	-1	--	--	--	-1	-1	--	--	--	--	-1	-1	-1
1A22.pdb_BT301A	-1	1.762	-1	-1	-1	-1	--	--	--	--	--	--	--	--	-1	-1
1A22.pdb_BT395A	-1	-0.094	-1	-1	-1	-1	--	--	--	--	--	--	--	--	--	--
1A22.pdb_BV371A	-1	-0.617	-1	-1	--	--	--	-1	-1	--	--	--	--	-1	-1	-1
1A22.pdb_BW276A	-1	0.514	1	1	--	--	--	-1	-1	--	--	--	--	1	-1	-1
1A22.pdb_BW280A	-1	-0.018	-1	-1	--	--	--	--	--	--	--	--	--	-1	--	--
1A4Y.pdb_AD435A	1	3.486	-1	1	--	--	--	-1	-1	-1	1	-1	-1	1	1	1
1A4Y.pdb_AE344A	-1	0.179	-1	-1	--	--	--	-1	-1	-1	-1	-1	1	-1	-1	-1
1A4Y.pdb_AE401A	-1	0.884	-1	-1	--	--	--	-1	-1	-1	-1	-1	--	-1	-1	-1
1A4Y.pdb_AI459A	-1	0.679	-1	-1	--	--	--	-1	-1	-1	-1	-1	--	-1	-1	-1
1A4Y.pdb_AK320A	-1	-0.310	-1	-1	--	--	--	-1	-1	-1	-1	-1	-1	-1	-1	-1
1A4Y.pdb_AS289A	-1	0.042	-1	-1	--	--	--	1	1	-1	-1	-1	-1	-1	-1	-1
1A4Y.pdb_AW261A	-1	0.101	-1	-1	--	--	--	--	--	-1	-1	-1	-1	-1	-1	-1
1A4Y.pdb_AW263A	-1	1.171	-1	1	--	--	--	-1	1	-1	-1	1	--	-1	-1	-1
1A4Y.pdb_AW318A	-1	1.501	-1	-1	--	--	--	-1	1	-1	-1	1	--	-1	-1	-1
1A4Y.pdb_AW375A	-1	1.035	-1	-1	--	--	--	1	1	-1	-1	1	--	1	-1	-1
1A4Y.pdb_AY434A	1	3.262	-1	1	--	--	--	-1	1	-1	-1	1	1	1	1	1

Supplementary Text S4: Hotspot Predictor Predictions and Performance

**1A4Y.pdb_AY437A**	-1	0.836	1	1	--	--	--	-1	-1	-1	-1	1	--	-1	1	1
**1A4Y.pdb_BE108A**	-1	-0.323	-1	-1	--	--	--	-1	-1	-1	-1	-1	-1	-1	-1	-1
**1A4Y.pdb_BH114A**	-1	0.657	-1	-1	--	--	--	1	1	-1	-1	-1	--	-1	-1	-1
**1A4Y.pdb_BH13A**	-1	-0.297	-1	-1	--	--	--	--	--	--	--	-1	--	-1	-1	-1
**1A4Y.pdb_BH84A**	-1	0.170	-1	-1	--	--	--	--	--	-1	-1	-1	1	-1	-1	-1
**1A4Y.pdb_BH8A**	-1	0.904	-1	-1	--	--	--	-1	-1	-1	-1	-1	--	-1	-1	-1
**1A4Y.pdb_BN68A**	-1	0.118	-1	-1	--	--	--	-1	-1	-1	-1	-1	-1	-1	-1	-1
**1A4Y.pdb_BQ12A**	-1	0.300	-1	-1	--	--	--	-1	-1	-1	-1	-1	1	-1	-1	-1
**1A4Y.pdb_BR31A**	-1	0.251	-1	-1	--	--	--	-1	-1	-1	-1	1	-1	-1	1	1
**1A4Y.pdb_BR32A**	-1	0.910	-1	-1	--	--	--	-1	-1	-1	-1	-1	--	-1	-1	-1
**1A4Y.pdb_BR5A**	1	2.309	1	-1	--	--	--	--	--	-1	-1	1	1	-1	1	1
**1A4Y.pdb_BW89A**	-1	0.240	-1	-1	--	--	--	-1	-1	-1	-1	1	-1	-1	-1	1
**1AHW.pdb_CL176A**	-1	0.987	-1	-1	--	--	--	--	--	--	--	-1	--	-1	--	--
**1AHW.pdb_CT167A**	-1	-0.074	-1	-1	--	--	--	-1	-1	-1	-1	-1	-1	-1	1	1
**1AHW.pdb_CT170A**	-1	1.106	-1	-1	--	--	--	-1	-1	-1	-1	-1	--	-1	-1	-1
**1AHW.pdb_CT197A**	-1	1.346	-1	-1	--	--	--	--	--	--	--	-1	--	-1	-1	-1
**1AHW.pdb_CV198A**	-1	-0.314	-1	-1	--	--	--	-1	-1	-1	-1	-1	-1	-1	-1	-1
**1AHW.pdb_CY157A**	-1	-1.890	-1	-1	-1	-1	--	--	--	--	--	--	--	--	--	--
**1AK4.pdb_DG489A**	1	3.442	-1	-1	-1	-1	1	-1	-1	--	--	--	--	--	--	--
**1AK4.pdb_DH487A**	1	2.374	-1	-1	-1	-1	-1	-1	-1	--	--	--	--	--	--	--
**1AK4.pdb_DI491A**	-1	1.604	1	-1	-1	-1	-1	-1	-1	--	--	--	--	--	--	--
**1AK4.pdb_DP485A**	1	2.450	-1	-1	-1	-1	-1	-1	-1	--	--	--	--	--	--	--
**1AK4.pdb_DP490A**	1	3.537	-1	-1	1	-1	1	1	1	--	--	--	--	--	--	--
**1AK4.pdb_DP493A**	1	2.047	-1	-1	-1	-1	-1	-1	-1	--	--	--	--	--	--	--
**1AK4.pdb_DV486A**	1	2.356	-1	-1	-1	-1	--	--	--	--	--	--	--	--	--	--
**1BRS.pdb_AE60A**	-1	-0.326	-1	-1	--	--	--	-1	1	-1	1	-1	-1	-1	1	1
**1BRS.pdb_AE73A**	1	2.348	-1	-1	--	--	--	--	--	--	--	-1	--	-1	-1	-1
**1BRS.pdb_AH102A**	1	6.146	-1	1	--	--	--	1	1	-1	-1	1	1	1	1	1
**1BRS.pdb_AK27A**	1	5.381	1	-1	--	--	--	-1	1	-1	-1	-1	-1	-1	-1	1
**1BRS.pdb_AN58A**	1	3.066	-1	-1	--	--	--	-1	-1	-1	-1	-1	-1	-1	-1	-1
**1BRS.pdb_AR59A**	1	5.245	1	1	--	--	--	-1	-1	-1	-1	1	-1	1	1	1
**1BRS.pdb_AR87A**	1	5.565	1	-1	--	--	--	--	--	-1	1	1	1	-1	1	1
**1BRS.pdb_DD35A**	1	4.503	-1	-1	--	--	--	1	1	1	1	-1	1	1	1	1
**1BRS.pdb_DD39A**	1	7.651	-1	1	--	--	--	-1	-1	1	-1	1	1	1	1	1
**1BRS.pdb_DE76A**	-1	1.364	-1	-1	--	--	--	-1	-1	-1	-1	-1	--	-1	-1	-1
**1BRS.pdb_DE80A**	-1	0.543	-1	-1	-1	-1	--	--	--	--	--	-1	--	--	--	--
**1BRS.pdb_DT42A**	-1	1.858	-1	-1	--	--	--	-1	-1	-1	-1	-1	--	-1	1	1
**1BRS.pdb_DY29A**	1	3.471	1	1	--	--	--	-1	-1	1	-1	1	-1	-1	1	1
**1CBW.pdb_IG12A**	-1	0.686	-1	-1	-1	-1	--	--	--	--	--	--	--	--	--	--
**1CBW.pdb_IG36A**	-1	0.964	-1	-1	-1	-1	1	1	1	--	--	--	--	--	--	--
**1CBW.pdb_IG37A**	-1	0.821	-1	-1	-1	-1	1	-1	-1	--	--	--	--	--	--	--
**1CBW.pdb_II18A**	-1	1.416	-1	-1	-1	-1	--	--	--	--	--	--	--	--	--	--
**1CBW.pdb_II19A**	-1	0.143	-1	-1	-1	-1	-1	-1	-1	--	--	--	--	--	--	--
**1CBW.pdb_IK15A**	1	2.015	1	1	1	1	-1	1	1	--	--	--	--	--	--	--
**1CBW.pdb_IP13A**	-1	-0.056	-1	-1	-1	-1	-1	-1	-1	--	--	--	--	--	--	--
**1CBW.pdb_IR17A**	-1	0.554	1	-1	1	1	-1	1	1	--	--	--	--	--	--	--
**1CBW.pdb_IR39A**	-1	0.222	-1	-1	-1	-1	-1	1	1	--	--	--	--	--	--	--
**1CBW.pdb_IT11A**	-1	0.222	-1	-1	-1	-1	--	--	--	--	--	--	--	--	--	--
**1CBW.pdb_IV34A**	-1	0.052	-1	-1	-1	-1	--	--	--	--	--	--	--	--	--	--
**1CHO.pdb_IE19A**	1	2.333	-1	-1	1	-1	-1	-1	-1	--	--	--	--	--	--	--
**1CHO.pdb_IG32A**	-1	-1.092	-1	-1	-1	-1	--	--	--	--	--	--	--	--	--	--
**1CHO.pdb_IK13A**	-1	0.181	-1	-1	-1	-1	--	--	--	--	--	--	--	--	--	--
**1CHO.pdb_IL18A**	1	4.766	1	1	1	1	1	-1	1	--	--	--	--	--	--	--
**1CHO.pdb_IN36A**	-1	-1.364	-1	-1	-1	-1	-1	-1	-1	--	--	--	--	--	--	--
**1CHO.pdb_IP14A**	-1	0.381	-1	-1	-1	-1	-1	-1	-1	--	--	--	--	--	--	--
**1CHO.pdb_IR21A**	1	3.192	-1	-1	-1	-1	-1	-1	-1	--	--	--	--	--	--	--
**1CHO.pdb_IT17A**	1	4.159	-1	-1	1	-1	-1	-1	1	--	--	--	--	--	--	--
**1CHO.pdb_IY20A**	1	2.543	1	1	1	1	1	1	1	--	--	--	--	--	--	--
**1DAN.pdb_HM175A**	-1	0.744	-1	-1	1	1	1	1	1	--	--	--	--	--	--	--
**1DAN.pdb_TD44A**	-1	1.380	-1	-1	--	--	--	-1	1	-1	-1	--	--	-1	1	-1
**1DAN.pdb_TD58A**	-1	1.989	-1	-1	--	--	--	-1	-1	1	-1	--	-1	-1	1	1
**1DAN.pdb_TD61A**	-1	0.242	-1	1	--	--	--	--	--	1	-1	--	-1	-1	-1	-1
**1DAN.pdb_TE24A**	-1	0.658	-1	-1	--	--	--	-1	-1	-1	-1	--	--	-1	-1	-1
**1DAN.pdb_TF50A**	-1	0.438	-1	-1	--	--	--	1	1	-1	-1	--	--	-1	-1	-1
**1DAN.pdb_TF76A**	-1	1.105	-1	-1	--	--	--	-1	-1	-1	-1	--	--	-1	-1	-1
**1DAN.pdb_TG43A**	-1	0.065	-1	-1	-1	-1	-1	-1	-1	--	--	--	--	--	--	--
**1DAN.pdb_TI22A**	-1	0.645	-1	-1	--	--	--	-1	-1	-1	1	--	--	-1	-1	-1
**1DAN.pdb_TK15A**	-1	-0.397	-1	-1	--	--	--	--	--	--	--	--	--	-1	--	--
**1DAN.pdb_TK20A**	1	2.439	1	-1	--	--	--	-1	-1	1	-1	--	-1	-1	1	1
**1DAN.pdb_TK41A**	-1	0.322	-1	-1	--	--	--	--	--	-1	-1	--	-1	-1	-1	-1
**1DAN.pdb_TK46A**	-1	0.895	-1	-1	--	--	--	-1	-1	--	--	--	--	-1	-1	-1
**1DAN.pdb_TK48A**	-1	0.921	-1	-1	--	--	--	--	--	-1	-1	--	--	-1	-1	-1
**1DAN.pdb_TN18A**	-1	0.180	-1	-1	--	--	--	-1	-1	--	--	--	--	-1	-1	-1
**1DAN.pdb_TQ37A**	-1	0.729	-1	-1	--	--	--	-1	-1	1	1	--	--	-1	-1	-1
**1DAN.pdb_TS42A**	-1	-0.069	-1	-1	--	--	--	--	--	-1	-1	--	-1	-1	-1	-1
**1DAN.pdb_TS47A**	-1	-0.127	-1	-1	--	--	--	1	1	-1	-1	--	-1	-1	-1	-1
**1DAN.pdb_TT17A**	-1	0.121	-1	-1	--	--	--	-1	-1	1	-1	--	-1	-1	-1	-1
**1DAN.pdb_TW45A**	-1	1.500	-1	-1	--	--	--	-1	1	-1	1	--	--	1	-1	-1

**1DAN.pdb_TY51A**	-1	-0.127	-1	-1	-1	-1	--	--	--	--	--	--	--	--	-1	-1
**1DAN.pdb_TY78A**	-1	0.628	-1	-1	-1	-1	--	--	--	--	--	--	--	--	--	--
**1DAN.pdb_UE128A**	-1	0.086	-1	-1	--	--	--	-1	-1	-1	-1	--	-1	-1	-1	-1
**1DAN.pdb_UE208A**	-1	-0.005	-1	-1	--	--	--	--	--	-1	-1	--	-1	-1	-1	-1
**1DAN.pdb_UF140A**	1	2.215	-1	1	--	--	--	-1	-1	-1	-1	--	--	-1	-1	-1
**1DAN.pdb_UL133A**	-1	-0.028	1	-1	--	--	--	-1	-1	-1	-1	--	-1	-1	-1	-1
**1DAN.pdb_UN138A**	-1	0.000	-1	-1	-1	-1	--	--	--	--	--	--	--	--	--	--
**1DAN.pdb_UP92A**	-1	-0.186	-1	-1	-1	-1	--	--	--	--	--	--	--	--	--	--
**1DAN.pdb_UQ110A**	-1	1.305	-1	-1	--	--	--	-1	-1	-1	-1	--	--	-1	-1	-1
**1DAN.pdb_UR131A**	-1	0.000	-1	-1	--	--	--	-1	-1	-1	-1	--	-1	-1	-1	-1
**1DAN.pdb_UR135A**	-1	0.986	-1	-1	--	--	--	-1	-1	-1	-1	--	--	-1	-1	-1
**1DAN.pdb_UT132A**	-1	0.000	-1	-1	--	--	--	-1	-1	1	1	--	-1	-1	-1	-1
**1DAN.pdb_UT203A**	-1	0.135	-1	-1	--	--	--	-1	-1	-1	-1	--	-1	-1	-1	-1
**1DAN.pdb_UV207A**	-1	1.570	1	1	--	--	--	-1	1	-1	-1	--	1	1	-1	-1
**1DAN.pdb_UY94A**	-1	0.312	1	1	--	--	--	1	1	-1	-1	--	--	-1	1	1
**1DQJ.pdb_AN31A**	1	2.014	-1	-1	-1	-1	-1	-1	-1	--	--	--	--	--	--	--
**1DQJ.pdb_AN32A**	1	4.093	1	-1	1	-1	-1	1	1	--	--	--	--	--	--	--
**1DQJ.pdb_AS91A**	-1	1.433	-1	-1	-1	-1	-1	-1	-1	--	--	--	--	--	--	--
**1DQJ.pdb_AY50A**	1	2.679	1	-1	-1	-1	1	-1	-1	--	--	--	--	--	--	--
**1DQJ.pdb_AY96A**	-1	1.136	-1	-1	1	1	1	-1	-1	--	--	--	--	--	--	--
**1DQJ.pdb_BD32A**	1	2.014	1	-1	1	-1	-1	-1	-1	--	--	--	--	--	--	--
**1DQJ.pdb_BW98A**	1	4.934	1	1	1	1	1	1	1	--	--	--	--	--	--	--
**1DQJ.pdb_BY33A**	1	5.527	1	1	1	1	1	-1	-1	--	--	--	--	--	--	--
**1DQJ.pdb_BY50A**	1	6.891	1	-1	1	1	1	1	1	--	--	--	--	--	--	--
**1DQJ.pdb_BY53A**	-1	1.181	1	1	1	1	1	-1	-1	--	--	--	--	--	--	--
**1DQJ.pdb_CD101A**	-1	1.453	-1	1	1	-1	-1	-1	1	--	--	--	--	--	--	--
**1DQJ.pdb_CK96A**	1	6.159	1	1	1	1	-1	-1	1	--	--	--	--	--	--	--
**1DQJ.pdb_CK97A**	1	3.521	1	1	1	1	-1	1	1	--	--	--	--	--	--	--
**1DQJ.pdb_CL75A**	-1	1.453	1	1	1	1	1	1	1	--	--	--	--	--	--	--
**1DQJ.pdb_CN93A**	-1	0.650	-1	-1	1	-1	-1	-1	-1	--	--	--	--	--	--	--
**1DQJ.pdb_CR21A**	-1	1.169	-1	-1	1	-1	-1	-1	-1	--	--	--	--	--	--	--
**1DQJ.pdb_CS100A**	-1	0.776	-1	-1	1	-1	1	-1	-1	--	--	--	--	--	--	--
**1DQJ.pdb_CT89A**	-1	0.841	-1	-1	-1	-1	-1	-1	-1	--	--	--	--	--	--	--
**1DQJ.pdb_CW62A**	-1	0.758	-1	-1	-1	-1	-1	-1	-1	--	--	--	--	--	--	--
**1DQJ.pdb_CW63A**	-1	1.347	-1	-1	-1	-1	1	-1	-1	--	--	--	--	--	--	--
**1DQJ.pdb_CY20A**	1	3.287	1	-1	1	1	1	1	1	--	--	--	--	--	--	--
**1DVF.pdb_AH30A**	-1	1.650	-1	-1	-1	-1	-1	-1	-1	-1	-1	--	--	--	-1	-1
**1DVF.pdb_AS93A**	-1	1.163	-1	-1	-1	-1	--	--	--	--	--	--	--	--	-1	-1
**1DVF.pdb_AW92A**	-1	0.341	1	1	1	1	1	-1	1	-1	-1	--	-1	--	-1	-1
**1DVF.pdb_AY32A**	1	2.031	1	-1	1	1	--	--	--	-1	1	--	-1	--	-1	-1
**1DVF.pdb_AY49A**	-1	1.629	-1	-1	-1	-1	--	--	--	-1	-1	--	--	--	-1	-1
**1DVF.pdb_AY50A**	-1	0.688	-1	-1	-1	-1	--	--	--	-1	-1	--	--	--	-1	-1
**1DVF.pdb_BD100A**	1	2.791	-1	-1	--	--	--	-1	1	-1	1	--	-1	-1	-1	-1
**1DVF.pdb_BD54A**	1	4.283	-1	-1	--	--	--	1	1	-1	1	--	-1	-1	1	1
**1DVF.pdb_BD58A**	-1	1.601	-1	-1	--	--	--	-1	-1	-1	-1	--	--	-1	-1	-1
**1DVF.pdb_BE98A**	1	4.189	-1	-1	--	--	--	-1	-1	-1	1	--	1	1	-1	-1
**1DVF.pdb_BN56A**	-1	1.163	-1	-1	--	--	--	-1	-1	-1	-1	--	--	-1	-1	-1
**1DVF.pdb_BR99A**	-1	1.875	-1	-1	--	--	--	--	--	-1	-1	--	--	-1	-1	-1
**1DVF.pdb_BT30A**	-1	0.908	-1	-1	--	--	--	-1	-1	-1	-1	--	--	-1	-1	-1
**1DVF.pdb_BW52A**	1	4.135	-1	-1	--	--	--	-1	-1	-1	1	--	1	1	1	1
**1DVF.pdb_BY32A**	-1	1.832	-1	-1	--	--	--	--	--	-1	-1	--	--	-1	-1	-1
**1DVF.pdb_CY49A**	-1	1.861	-1	-1	-1	-1	1	-1	-1	-1	-1	--	--	--	-1	-1
**1DVF.pdb_DD52A**	-1	1.683	-1	-1	-1	-1	--	--	--	--	--	--	--	--	-1	-1
**1DVF.pdb_DH33A**	-1	1.861	-1	-1	--	--	--	-1	-1	-1	-1	--	--	-1	-1	-1
**1DVF.pdb_DI101A**	1	2.683	-1	-1	--	--	--	-1	-1	--	--	--	--	--	--	--
**1DVF.pdb_DK30A**	-1	1.004	-1	-1	--	--	--	--	--	-1	-1	--	--	-1	-1	-1
**1DVF.pdb_DN55A**	-1	1.861	-1	-1	-1	-1	--	--	--	--	--	--	--	--	--	--
**1DVF.pdb_DQ104A**	-1	1.629	-1	-1	1	-1	1	-1	1	--	--	--	--	--	--	--
**1DVF.pdb_DR106A**	1	4.093	-1	-1	1	1	1	-1	1	--	--	--	--	--	--	--
**1DVF.pdb_DY102A**	1	4.742	1	1	--	--	--	-1	-1	--	--	--	--	--	--	--
**1EAW.pdb_AD106A**	-1	0.654	-1	-1	-1	-1	--	--	--	--	--	--	--	--	--	--
**1EAW.pdb_AD230A**	1	2.229	-1	-1	-1	-1	-1	1	1	--	--	--	--	--	--	--
**1EAW.pdb_AD62A**	-1	1.503	-1	-1	-1	-1	1	-1	-1	--	--	--	--	--	--	--
**1EAW.pdb_AF107A**	-1	0.892	-1	-1	-1	-1	-1	-1	-1	--	--	--	--	--	--	--
**1EAW.pdb_AF65A**	-1	-0.429	-1	-1	-1	-1	--	--	--	--	--	--	--	--	--	--
**1EAW.pdb_AH153A**	-1	-0.014	-1	-1	-1	-1	1	-1	1	--	--	--	--	--	--	--
**1EAW.pdb_AI41A**	-1	-0.823	-1	-1	1	1	1	1	1	--	--	--	--	--	--	--
**1EAW.pdb_AQ38A**	-1	-0.519	-1	-1	-1	-1	-1	-1	-1	--	--	--	--	--	--	--
**1EAW.pdb_AR63A**	-1	0.588	-1	-1	1	-1	-1	-1	1	--	--	--	--	--	--	--
**1EAW.pdb_AT108A**	-1	0.255	-1	-1	-1	-1	--	--	--	--	--	--	--	--	--	--
**1EAW.pdb_AT160A**	-1	0.089	-1	-1	-1	-1	--	--	--	--	--	--	--	--	--	--
**1EAW.pdb_AY67A**	-1	-0.079	-1	-1	1	1	1	-1	1	--	--	--	--	--	--	--
**1EFN.pdb_AI96A**	-1	1.451	1	-1	1	1	1	-1	1	--	--	--	--	--	--	--
**1EMV.pdb_AC23A**	-1	0.922	-1	-1	-1	-1	1	-1	-1	--	--	--	--	--	--	--
**1EMV.pdb_AD51A**	1	5.918	-1	-1	-1	-1	--	--	--	--	--	--	--	--	--	--
**1EMV.pdb_AE30A**	-1	1.417	-1	-1	1	-1	1	-1	-1	--	--	--	--	--	--	--
**1EMV.pdb_AE41A**	1	2.084	-1	-1	-1	-1	-1	-1	-1	--	--	--	--	--	--	--
**1EMV.pdb_AG49A**	-1	1.486	-1	-1	-1	-1	--	--	--	--	--	--	--	--	--	--

**1EMV.pdb_AH46A**	-1	0.832	-1	-1	-1	-1	--	--	--	--	--	--	--	--	--	--
**1EMV.pdb_AI53A**	-1	0.848	-1	-1	1	1	1	-1	-1	--	--	--	--	--	--	--
**1EMV.pdb_AL33A**	1	3.419	1	-1	1	1	1	-1	-1	--	--	--	--	--	--	--
**1EMV.pdb_AN24A**	-1	0.139	-1	-1	-1	-1	--	--	--	--	--	--	--	--	--	--
**1EMV.pdb_AP47A**	-1	0.437	-1	-1	-1	-1	-1	-1	-1	--	--	--	--	--	--	--
**1EMV.pdb_AP56A**	-1	1.243	-1	-1	-1	-1	--	--	--	--	--	--	--	--	--	--
**1EMV.pdb_AS28A**	-1	0.173	-1	-1	-1	-1	--	--	--	--	--	--	--	--	--	--
**1EMV.pdb_AS29A**	-1	0.956	-1	-1	-1	-1	-1	-1	-1	--	--	--	--	--	--	--
**1EMV.pdb_AS48A**	-1	0.007	-1	-1	-1	-1	-1	-1	-1	--	--	--	--	--	--	--
**1EMV.pdb_AS50A**	1	2.188	-1	-1	1	-1	1	1	1	--	--	--	--	--	--	--
**1EMV.pdb_AT27A**	-1	0.728	-1	-1	-1	-1	--	--	--	--	--	--	--	--	--	--
**1EMV.pdb_AT38A**	-1	0.900	-1	-1	-1	-1	--	--	--	--	--	--	--	--	--	--
**1EMV.pdb_AV34A**	1	2.579	1	-1	1	-1	1	1	1	--	--	--	--	--	--	--
**1EMV.pdb_AV37A**	-1	1.665	-1	-1	1	-1	1	1	1	--	--	--	--	--	--	--
**1EMV.pdb_AY54A**	1	4.837	1	1	1	1	1	1	1	--	--	--	--	--	--	--
**1EMV.pdb_AY55A**	1	4.637	1	1	1	-1	-1	-1	-1	--	--	--	--	--	--	--
**1EMV.pdb_BF86A**	1	3.881	1	1	1	1	1	1	1	--	--	--	--	--	--	--
**1EMV.pdb_BK97A**	-1	1.961	1	1	1	1	-1	-1	-1	--	--	--	--	--	--	--
**1EMV.pdb_BN72A**	-1	1.165	-1	-1	1	-1	-1	-1	-1	--	--	--	--	--	--	--
**1EMV.pdb_BN75A**	1	2.336	1	-1	1	1	-1	-1	-1	--	--	--	--	--	--	--
**1EMV.pdb_BQ92A**	-1	-0.278	-1	-1	-1	-1	1	-1	-1	--	--	--	--	--	--	--
**1EMV.pdb_BR54A**	-1	1.666	-1	-1	-1	-1	--	--	--	--	--	--	--	--	--	--
**1EMV.pdb_BS74A**	-1	-0.241	-1	-1	1	-1	-1	-1	-1	--	--	--	--	--	--	--
**1EMV.pdb_BS77A**	-1	-0.233	-1	-1	-1	-1	-1	-1	-1	--	--	--	--	--	--	--
**1EMV.pdb_BS78A**	-1	-0.540	-1	-1	1	-1	--	--	--	--	--	--	--	--	--	--
**1EMV.pdb_BS84A**	-1	-0.109	-1	-1	1	-1	-1	-1	-1	--	--	--	--	--	--	--
**1EMV.pdb_BT87A**	-1	0.159	-1	-1	-1	-1	-1	-1	-1	--	--	--	--	--	--	--
**1EMV.pdb_BV98A**	-1	1.089	-1	-1	-1	-1	--	--	--	--	--	--	--	--	--	--
**1F47.pdb_AD4A**	-1	0.692	-1	-1	--	--	--	-1	-1	--	--	--	--	-1	--	--
**1F47.pdb_AD7A**	-1	1.734	-1	-1	--	--	--	--	--	--	--	--	--	-1	--	--
**1F47.pdb_AF11A**	1	2.445	-1	1	--	--	--	1	1	--	--	--	--	-1	--	--
**1F47.pdb_AI8A**	1	2.516	1	1	--	--	--	1	1	--	--	--	--	-1	--	--
**1F47.pdb_AK14A**	-1	-0.043	-1	-1	-1	-1	--	--	--	--	--	--	--	--	--	--
**1F47.pdb_AL12A**	1	2.295	-1	-1	--	--	--	-1	-1	--	--	--	--	-1	--	--
**1F47.pdb_AL6A**	-1	0.925	-1	-1	--	--	--	-1	-1	--	--	--	--	-1	--	--
**1F47.pdb_AP9A**	-1	-0.058	-1	-1	-1	-1	--	--	--	--	--	--	--	--	--	--
**1F47.pdb_AQ15A**	-1	-0.046	-1	1	--	--	--	-1	-1	--	--	--	--	-1	--	--
**1F47.pdb_AY5A**	-1	0.869	-1	1	--	--	--	-1	-1	--	--	--	--	-1	--	--
**1FC2.pdb_CI150A**	1	5.277	1	-1	--	--	--	1	1	--	--	--	--	1	-1	-1
**1FC2.pdb_CK154A**	-1	1.527	-1	-1	--	--	--	-1	-1	--	--	--	--	-1	-1	-1
**1FC2.pdb_CN147A**	-1	0.606	-1	-1	--	--	--	-1	-1	--	--	--	--	-1	-1	-1
**1FCC.pdb_CD40A**	-1	0.272	-1	1	--	--	--	-1	-1	--	--	-1	--	-1	-1	-1
**1FCC.pdb_CE42A**	-1	0.385	-1	-1	--	--	--	-1	-1	--	--	-1	--	-1	-1	-1
**1FCC.pdb_CK28A**	-1	1.256	1	1	--	--	--	1	1	--	--	-1	--	-1	1	1
**1FCC.pdb_CK31A**	1	3.478	-1	-1	--	--	--	-1	1	--	--	-1	--	1	1	1
**1FCC.pdb_CN35A**	1	2.365	-1	-1	--	--	--	-1	1	--	--	-1	--	1	1	1
**1FCC.pdb_CT25A**	-1	0.240	-1	-1	--	--	--	--	--	--	--	-1	--	-1	-1	-1
**1FCC.pdb_CW43A**	1	3.773	1	1	--	--	--	-1	-1	--	--	1	--	1	1	1
**1FFW.pdb_BC213A**	-1	0.204	-1	-1	-1	-1	-1	-1	-1	--	--	--	--	--	--	--
**1FFW.pdb_BD207A**	-1	0.096	-1	-1	-1	-1	-1	-1	-1	--	--	--	--	--	--	--
**1FFW.pdb_BE171A**	-1	0.717	-1	-1	-1	-1	--	--	--	--	--	--	--	--	--	--
**1FFW.pdb_BE178A**	-1	0.639	-1	-1	1	-1	1	-1	-1	--	--	--	--	--	--	--
**1FFW.pdb_BF214A**	1	3.646	1	1	1	1	1	-1	-1	--	--	--	--	--	--	--
**1FFW.pdb_BH181A**	-1	0.034	-1	-1	-1	-1	-1	-1	-1	--	--	--	--	--	--	--
**1GC1.pdb_CD63A**	-1	-0.319	-1	-1	--	--	--	-1	-1	-1	-1	-1	-1	-1	-1	-1
**1GC1.pdb_CE85A**	-1	1.323	-1	-1	--	--	--	--	--	--	--	1	--	-1	-1	-1
**1GC1.pdb_CH27A**	-1	0.283	-1	-1	--	--	--	-1	1	-1	-1	-1	-1	-1	-1	-1
**1GC1.pdb_CK29A**	-1	0.536	-1	-1	--	--	--	1	1	-1	-1	1	--	-1	-1	-1
**1GC1.pdb_CK35A**	-1	0.322	-1	-1	--	--	--	-1	1	1	-1	-1	-1	-1	-1	-1
**1GC1.pdb_CK46A**	-1	1.431	-1	-1	-1	-1	-1	-1	-1	--	--	--	--	--	--	--
**1GC1.pdb_CL44A**	-1	1.056	-1	-1	--	--	--	-1	-1	-1	-1	-1	--	-1	-1	-1
**1GC1.pdb_CN32A**	-1	0.183	-1	-1	--	--	--	--	--	-1	-1	-1	-1	-1	-1	-1
**1GC1.pdb_CN52A**	-1	0.708	-1	-1	--	--	--	-1	-1	-1	-1	-1	--	-1	-1	-1
**1GC1.pdb_CQ25A**	-1	0.032	-1	-1	--	--	--	--	--	-1	-1	-1	-1	-1	-1	-1
**1GC1.pdb_CQ33A**	-1	0.105	-1	-1	--	--	--	-1	-1	-1	-1	-1	-1	-1	-1	-1
**1GC1.pdb_CQ40A**	-1	-0.411	-1	-1	--	--	--	-1	-1	1	-1	-1	1	1	1	-1
**1GC1.pdb_CQ64A**	-1	0.443	-1	-1	--	--	--	-1	-1	-1	-1	-1	--	-1	-1	-1
**1GC1.pdb_CR59A**	-1	1.176	-1	-1	--	--	--	-1	-1	-1	-1	-1	--	-1	1	1
**1GC1.pdb_CS23A**	-1	0.293	-1	-1	--	--	--	--	--	--	--	-1	--	-1	-1	-1
**1GC1.pdb_CS42A**	-1	0.000	-1	-1	--	--	--	-1	-1	-1	-1	-1	-1	-1	1	1
**1GC1.pdb_CS60A**	-1	-0.089	-1	-1	--	--	--	-1	-1	-1	-1	-1	-1	-1	-1	-1
**1GC1.pdb_CT45A**	-1	-0.149	-1	-1	--	--	--	-1	-1	-1	-1	-1	-1	-1	-1	-1
**1GCQ.pdb_CP595A**	-1	0.768	-1	-1	1	-1	-1	-1	-1	--	--	--	--	--	--	--
**1GCQ.pdb_CP608A**	-1	0.121	-1	-1	-1	-1	-1	-1	-1	--	--	--	--	--	--	--
**1GCQ.pdb_CP609A**	-1	0.085	-1	-1	-1	-1	-1	-1	-1	--	--	--	--	--	--	--
**1GCQ.pdb_CP657A**	-1	1.316	-1	-1	1	-1	-1	-1	-1	--	--	--	--	--	--	--
**1H9D.pdb_BG61A**	1	2.077	-1	-1	-1	-1	-1	-1	-1	--	--	--	--	--	--	--
**1H9D.pdb_BL103A**	-1	0.940	-1	-1	1	1	1	-1	1	--	--	--	--	--	--	--

**1H9D.pdb_BN104A**	1	2.304	-1	-1	1	-1	1	1	1	--	--	--	--	--	--	--
**1H9D.pdb_BQ67A**	-1	1.364	-1	-1	1	-1	1	-1	-1	--	--	--	--	--	--	--
**1H9D.pdb_BR3A**	-1	1.162	-1	-1	1	-1	-1	-1	-1	--	--	--	--	--	--	--
**1H9D.pdb_BV4A**	-1	1.402	-1	-1	-1	-1	--	--	--	--	--	--	--	--	--	--
**1IAR.pdb_AF82A**	-1	-0.086	-1	-1	-1	-1	--	--	--	--	--	--	--	--	--	--
**1IAR.pdb_AI5A**	-1	1.171	1	-1	-1	-1	-1	1	1	--	--	--	--	--	--	--
**1IAR.pdb_AN89A**	-1	1.559	-1	-1	1	1	1	-1	1	--	--	--	--	--	--	--
**1IAR.pdb_AQ78A**	-1	0.125	-1	-1	-1	-1	--	--	--	--	--	--	--	--	--	--
**1IAR.pdb_AQ8A**	-1	-0.022	-1	-1	-1	-1	--	--	--	--	--	--	--	--	--	--
**1IAR.pdb_AR81A**	-1	0.480	-1	-1	-1	-1	-1	-1	-1	--	--	--	--	--	--	--
**1IAR.pdb_AR85A**	-1	0.427	1	1	1	1	1	1	1	--	--	--	--	--	--	--
**1IAR.pdb_AR88A**	1	3.755	-1	-1	1	1	-1	1	1	--	--	--	--	--	--	--
**1IAR.pdb_AT13A**	-1	0.979	-1	-1	-1	-1	--	--	--	--	--	--	--	--	--	--
**1IAR.pdb_AT6A**	-1	-0.104	-1	-1	-1	-1	--	--	--	--	--	--	--	--	--	--
**1IAR.pdb_AW91A**	-1	0.730	-1	-1	-1	-1	1	-1	1	--	--	--	--	--	--	--
**1JCK.pdb_BF176A**	1	2.134	1	-1	--	--	--	-1	-1	--	--	-1	--	-1	-1	-1
**1JCK.pdb_BK103A**	-1	0.677	-1	-1	--	--	--	-1	-1	--	--	-1	--	-1	-1	-1
**1JCK.pdb_BN60A**	-1	1.643	-1	-1	--	--	--	--	--	--	--	-1	--	-1	-1	-1
**1JCK.pdb_BT20A**	-1	1.655	1	1	--	--	--	-1	-1	--	--	-1	--	-1	-1	-1
**1JCK.pdb_BV91A**	1	2.233	1	-1	--	--	--	-1	-1	--	--	-1	--	-1	-1	-1
**1JCK.pdb_BY26A**	-1	1.775	-1	-1	--	--	--	-1	-1	--	--	-1	--	-1	-1	-1
**1JCK.pdb_BY90A**	1	2.596	-1	-1	--	--	--	-1	-1	--	--	-1	--	-1	-1	-1
**1JRH.pdb_HD56A**	-1	1.887	-1	-1	--	--	--	-1	-1	--	--	--	--	-1	-1	-1
**1JRH.pdb_HD58A**	-1	1.855	-1	1	--	--	--	--	--	--	--	--	--	--	--	--
**1JRH.pdb_HH107A**	-1	1.699	-1	-1	1	1	1	-1	1	--	--	--	--	--	--	--
**1JRH.pdb_HR100A**	-1	0.543	-1	-1	--	--	--	--	--	--	--	--	--	--	--	--
**1JRH.pdb_HW54A**	1	2.687	-1	-1	--	--	--	--	--	--	--	--	--	--	--	--
**1JRH.pdb_HW55A**	1	2.422	-1	-1	--	--	--	-1	-1	--	--	--	--	--	--	--
**1JRH.pdb_HY104A**	-1	1.062	-1	1	--	--	--	-1	-1	--	--	--	--	--	--	--
**1JRH.pdb_HY32A**	-1	1.434	-1	-1	--	--	--	-1	1	--	--	--	--	-1	-1	-1
**1JRH.pdb_HY60A**	-1	1.256	-1	-1	--	--	--	--	--	--	--	--	--	--	--	--
**1JRH.pdb_IE55A**	-1	-0.435	-1	-1	--	--	--	-1	-1	--	--	--	--	-1	-1	-1
**1JRH.pdb_IG50A**	1	4.527	1	-1	1	-1	-1	-1	-1	--	--	--	--	--	--	--
**1JRH.pdb_IK47A**	1	3.579	1	-1	--	--	--	-1	-1	--	--	--	--	1	1	1
**1JRH.pdb_IK52A**	1	2.985	1	-1	--	--	--	-1	-1	--	--	--	--	-1	1	1
**1JRH.pdb_IK98A**	-1	-0.043	-1	-1	--	--	--	--	--	--	--	--	--	-1	-1	-1
**1JRH.pdb_IN48A**	-1	-0.293	1	-1	-1	-1	--	--	--	--	--	--	--	--	--	--
**1JRH.pdb_IN53A**	1	3.894	-1	-1	--	--	--	-1	-1	--	--	--	--	1	1	1
**1JRH.pdb_IR84A**	-1	-0.246	-1	-1	--	--	--	--	--	--	--	--	--	-1	-1	-1
**1JRH.pdb_IS54A**	-1	0.298	-1	-1	--	--	--	-1	-1	--	--	--	--	-1	1	1
**1JRH.pdb_IV51A**	-1	1.884	-1	-1	--	--	--	-1	1	--	--	--	--	-1	1	1
**1JRH.pdb_IW82A**	1	4.529	-1	-1	--	--	--	-1	-1	--	--	--	--	1	-1	-1
**1JRH.pdb_IY49A**	1	3.401	1	-1	--	--	--	1	1	--	--	--	--	-1	1	1
**1JRH.pdb_LD28A**	-1	0.435	-1	-1	--	--	--	--	--	--	--	--	--	-1	-1	-1
**1JRH.pdb_LE27A**	-1	0.543	-1	-1	--	--	--	--	--	--	--	--	--	-1	-1	-1
**1JRH.pdb_LS93A**	-1	-0.651	-1	1	--	--	--	-1	-1	--	--	--	--	-1	1	1
**1JRH.pdb_LT94A**	-1	0.385	-1	-1	--	--	--	-1	-1	--	--	--	--	-1	-1	-1
**1JRH.pdb_LW92A**	1	2.820	1	-1	--	--	--	1	1	--	--	--	--	1	1	1
**1JRH.pdb_LY30A**	-1	1.109	-1	-1	--	--	--	-1	1	--	--	--	--	-1	-1	-1
**1JRH.pdb_LY91A**	-1	0.581	-1	1	--	--	--	--	--	--	--	--	--	-1	1	1
**1JTG.pdb_AE104A**	-1	1.553	1	-1	--	--	--	-1	1	1	1	--	--	1	1	1
**1JTG.pdb_AE110A**	1	4.062	-1	-1	1	-1	-1	1	1	--	--	--	--	--	--	--
**1JTG.pdb_AE168A**	-1	-0.073	-1	-1	-1	-1	-1	-1	-1	--	--	--	--	--	--	--
**1JTG.pdb_AM129A**	-1	0.739	-1	-1	1	-1	1	-1	-1	--	--	--	--	--	--	--
**1JTG.pdb_AN100A**	-1	-0.456	-1	-1	-1	-1	-1	-1	-1	--	--	--	--	--	--	--
**1JTG.pdb_AP107A**	-1	-0.383	-1	-1	1	-1	1	-1	-1	--	--	--	--	--	--	--
**1JTG.pdb_AQ99A**	-1	0.430	-1	-1	-1	-1	-1	-1	1	--	--	--	--	--	--	--
**1JTG.pdb_AR243A**	-1	1.339	-1	-1	--	--	--	--	--	-1	-1	--	--	-1	-1	1
**1JTG.pdb_AS130A**	-1	0.792	-1	-1	--	--	--	-1	-1	-1	-1	--	--	-1	1	1
**1JTG.pdb_AV103A**	-1	1.911	1	-1	1	1	1	-1	-1	--	--	--	--	--	--	--
**1JTG.pdb_AV216A**	-1	-0.407	-1	-1	1	-1	1	-1	-1	--	--	--	--	--	--	--
**1JTG.pdb_AY105A**	-1	-0.168	1	1	--	--	--	1	1	1	1	--	1	1	1	1
**1JTG.pdb_BD163A**	-1	-1.341	-1	-1	-1	-1	--	--	--	--	--	--	--	--	--	--
**1JTG.pdb_BD49A**	1	2.561	-1	-1	--	--	--	1	1	-1	1	--	--	-1	1	1
**1JTG.pdb_BF142A**	1	3.379	-1	-1	--	--	--	-1	-1	1	1	--	1	1	1	1
**1JTG.pdb_BF36A**	1	3.201	1	-1	1	1	1	-1	1	--	--	--	--	--	--	--
**1JTG.pdb_BH148A**	1	2.748	-1	-1	1	1	-1	-1	-1	--	--	--	--	--	--	--
**1JTG.pdb_BH41A**	1	3.250	-1	-1	1	1	--	--	--	--	--	--	--	--	--	--
**1JTG.pdb_BK74A**	1	3.560	1	-1	--	--	--	-1	-1	-1	-1	--	1	1	1	1
**1JTG.pdb_BR160A**	1	2.222	-1	1	-1	-1	-1	-1	-1	--	--	--	--	--	--	--
**1JTG.pdb_BS113A**	-1	-0.168	-1	-1	-1	-1	-1	-1	-1	--	--	--	--	--	--	--
**1JTG.pdb_BS71A**	-1	0.358	-1	-1	-1	-1	-1	-1	-1	--	--	--	--	--	--	--
**1JTG.pdb_BW112A**	1	3.011	1	-1	1	1	1	-1	-1	--	--	--	--	--	--	--
**1JTG.pdb_BW150A**	1	4.254	-1	-1	-1	-1	-1	1	1	--	--	--	--	--	--	--
**1JTG.pdb_BW162A**	1	2.341	1	1	1	1	-1	-1	1	--	--	--	--	--	--	--
**1JTG.pdb_BY143A**	-1	0.382	-1	-1	--	--	--	-1	-1	-1	-1	--	1	-1	1	-1
**1JTG.pdb_BY50A**	-1	-0.407	1	1	1	1	-1	1	1	--	--	--	--	--	--	--
**1JTG.pdb_BY53A**	1	2.077	1	-1	1	1	-1	1	1	--	--	--	--	--	--	--

Supplementary Text S4: Hotspot Predictor Predictions and Performance

**1KTZ.pdb_AR25A**	-1	1.482	-1	-1	-1	-1	-1	-1	-1	--	--	--	--	--	--	--
**1KTZ.pdb_AR94A**	1	2.884	-1	-1	-1	-1	-1	-1	-1	--	--	--	--	--	--	--
**1KTZ.pdb_BD118A**	-1	1.261	-1	-1	-1	-1	--	--	--	--	--	--	--	--	--	--
**1KTZ.pdb_BD32A**	-1	1.968	-1	-1	-1	-1	-1	-1	-1	--	--	--	--	--	--	--
**1KTZ.pdb_BE119A**	-1	1.941	-1	1	-1	-1	-1	-1	1	--	--	--	--	--	--	--
**1KTZ.pdb_BE55A**	-1	1.663	-1	-1	-1	-1	-1	-1	1	--	--	--	--	--	--	--
**1KTZ.pdb_BF30A**	1	3.427	-1	-1	1	1	1	-1	-1	--	--	--	--	--	--	--
**1KTZ.pdb_BI50A**	1	2.343	-1	-1	1	-1	1	-1	-1	--	--	--	--	--	--	--
**1KTZ.pdb_BI53A**	-1	1.817	-1	-1	-1	-1	1	-1	1	--	--	--	--	--	--	--
**1KTZ.pdb_BL27A**	1	2.271	-1	-1	1	1	1	1	1	--	--	--	--	--	--	--
**1KTZ.pdb_BS49A**	-1	0.773	-1	-1	-1	-1	--	--	--	--	--	--	--	--	--	--
**1KTZ.pdb_BS52A**	-1	0.663	-1	-1	1	-1	-1	-1	1	--	--	--	--	--	--	--
**1KTZ.pdb_BT51A**	-1	1.960	-1	-1	1	-1	-1	-1	1	--	--	--	--	--	--	--
**1KTZ.pdb_BV77A**	-1	0.862	-1	-1	-1	-1	--	--	--	--	--	--	--	--	--	--
**1LFD.pdb_AD51A**	-1	-0.579	-1	-1	-1	-1	-1	-1	-1	--	--	--	--	--	--	--
**1LFD.pdb_AD56A**	-1	-0.280	-1	-1	-1	-1	--	--	--	--	--	--	--	--	--	--
**1LFD.pdb_AK32A**	-1	1.326	1	1	1	1	-1	1	1	--	--	--	--	--	--	--
**1LFD.pdb_AK52A**	-1	1.179	-1	-1	-1	-1	-1	-1	-1	--	--	--	--	--	--	--
**1LFD.pdb_AR20A**	-1	1.136	-1	-1	-1	-1	--	--	--	--	--	--	--	--	--	--
**1NMB.pdb_HY103A**	1	2.142	-1	-1	--	--	--	-1	-1	--	--	--	--	--	--	--
**1PPF.pdb_IE19A**	-1	1.203	-1	-1	-1	-1	-1	-1	-1	--	--	--	--	--	--	--
**1PPF.pdb_IG32A**	-1	0.235	-1	-1	-1	-1	-1	-1	1	--	--	--	--	--	--	--
**1PPF.pdb_IL18A**	-1	1.071	1	-1	1	1	1	1	1	--	--	--	--	--	--	--
**1PPF.pdb_IN36A**	-1	-1.646	-1	-1	-1	-1	-1	-1	-1	--	--	--	--	--	--	--
**1PPF.pdb_IP14A**	-1	-0.124	-1	-1	-1	-1	-1	-1	-1	--	--	--	--	--	--	--
**1PPF.pdb_IR21A**	-1	0.208	-1	-1	-1	-1	-1	-1	-1	--	--	--	--	--	--	--
**1PPF.pdb_IT17A**	1	3.485	1	-1	1	-1	-1	-1	1	--	--	--	--	--	--	--
**1PPF.pdb_IY20A**	1	3.210	1	1	1	1	1	1	1	--	--	--	--	--	--	--
**1R0R.pdb_IE19A**	1	2.089	1	-1	1	1	1	-1	1	--	--	--	--	--	--	--
**1R0R.pdb_IG32A**	-1	1.298	-1	-1	-1	-1	--	--	--	--	--	--	--	--	--	--
**1R0R.pdb_IK13A**	-1	-0.610	-1	-1	-1	-1	-1	-1	-1	--	--	--	--	--	--	--
**1R0R.pdb_IL18A**	-1	0.315	-1	-1	1	1	1	-1	1	--	--	--	--	--	--	--
**1R0R.pdb_IP14A**	-1	-0.639	-1	-1	-1	-1	-1	-1	-1	--	--	--	--	--	--	--
**1R0R.pdb_IR21A**	-1	-0.096	-1	-1	-1	-1	-1	-1	-1	--	--	--	--	--	--	--
**1R0R.pdb_IT17A**	-1	1.173	-1	-1	1	-1	-1	1	1	--	--	--	--	--	--	--
**1R0R.pdb_IY20A**	1	5.475	1	1	1	-1	-1	-1	1	--	--	--	--	--	--	--
**1REW.pdb_CQ86A**	1	2.659	1	-1	1	1	1	1	1	--	--	--	--	--	--	--
**1S1Q.pdb_AD46A**	-1	0.966	-1	-1	-1	-1	-1	-1	-1	--	--	--	--	--	--	--
**1S1Q.pdb_AF44A**	-1	0.199	-1	-1	-1	-1	-1	-1	-1	--	--	--	--	--	--	--
**1S1Q.pdb_AF88A**	-1	0.775	-1	-1	-1	1	1	-1	-1	--	--	--	--	--	--	--
**1S1Q.pdb_AN45A**	-1	1.232	-1	-1	1	-1	-1	-1	-1	--	--	--	--	--	--	--
**1S1Q.pdb_AV43A**	-1	0.671	-1	-1	1	-1	1	-1	1	--	--	--	--	--	--	--
**1S1Q.pdb_AW75A**	-1	0.281	-1	-1	-1	-1	--	--	--	--	--	--	--	--	--	--
**1SMF.pdb_IE16A**	-1	1.013	-1	-1	-1	-1	-1	-1	-1	--	--	--	--	--	--	--
**1SMF.pdb_II13A**	1	3.511	1	1	1	-1	-1	-1	-1	--	--	--	--	--	--	--
**1SMF.pdb_IS12A**	-1	1.900	-1	-1	-1	-1	1	-1	-1	--	--	--	--	--	--	--
**1SMF.pdb_IT10A**	1	2.046	-1	-1	1	-1	-1	-1	-1	--	--	--	--	--	--	--
**1TM1.pdb_IE60A**	1	2.925	1	-1	1	1	1	-1	-1	--	--	--	--	--	--	--
**1TM1.pdb_IM59A**	-1	1.028	1	1	1	1	1	1	1	--	--	--	--	--	--	--
**1TM1.pdb_IR62A**	-1	1.256	-1	-1	-1	-1	-1	-1	-1	--	--	--	--	--	--	--
**1TM1.pdb_IR65A**	1	3.080	-1	-1	-1	-1	--	--	--	--	--	--	--	--	--	--
**1TM1.pdb_IR67A**	1	2.923	-1	-1	-1	-1	--	--	--	--	--	--	--	--	--	--
**1TM1.pdb_IT58A**	1	2.572	1	1	1	-1	1	1	1	--	--	--	--	--	--	--
**1TM1.pdb_IY61A**	1	2.180	1	1	1	-1	-1	-1	-1	--	--	--	--	--	--	--
**1UUZ.pdb_AC64A**	-1	0.651	-1	-1	1	-1	1	-1	1	--	--	--	--	--	--	--
**1UUZ.pdb_AH62A**	-1	1.775	-1	-1	1	-1	-1	-1	-1	--	--	--	--	--	--	--
**1VFB.pdb_AH30A**	-1	0.845	-1	-1	--	--	--	-1	-1	--	--	--	--	-1	-1	-1
**1VFB.pdb_AS93A**	-1	0.343	-1	-1	--	--	--	-1	-1	--	--	--	--	-1	-1	-1
**1VFB.pdb_AW92A**	1	2.728	1	1	--	--	--	-1	-1	--	--	--	--	-1	-1	-1
**1VFB.pdb_AY32A**	-1	1.340	1	-1	--	--	--	-1	-1	-1	-1	--	--	1	-1	-1
**1VFB.pdb_AY49A**	-1	0.798	-1	-1	--	--	--	--	--	-1	-1	--	--	-1	-1	-1
**1VFB.pdb_AY50A**	-1	0.388	-1	-1	--	--	--	-1	-1	--	--	--	--	-1	-1	-1
**1VFB.pdb_BD100A**	1	3.072	-1	1	--	--	--	-1	-1	--	--	--	--	-1	1	1
**1VFB.pdb_BD54A**	-1	0.639	-1	-1	-1	-1	-1	-1	-1	--	--	--	--	--	-1	-1
**1VFB.pdb_BR99A**	-1	-0.100	-1	-1	--	--	--	--	--	--	--	--	--	-1	-1	-1
**1VFB.pdb_BT30A**	-1	-0.056	-1	-1	--	--	--	--	--	--	--	--	--	-1	-1	-1
**1VFB.pdb_BW52A**	-1	0.364	-1	-1	--	--	--	1	1	-1	-1	--	--	-1	-1	-1
**1VFB.pdb_BY32A**	-1	0.460	-1	-1	--	--	--	--	--	--	--	--	--	-1	-1	-1
**1VFB.pdb_CD119A**	-1	0.954	-1	-1	--	--	--	-1	-1	--	--	-1	--	-1	1	-1
**1VFB.pdb_CD18A**	-1	0.340	-1	-1	--	--	--	--	--	--	--	-1	--	-1	-1	-1
**1VFB.pdb_CI124A**	-1	1.232	-1	-1	--	--	--	-1	-1	--	--	-1	--	-1	-1	-1
**1VFB.pdb_CK116A**	-1	0.714	-1	-1	--	--	--	--	--	--	--	-1	--	-1	-1	-1
**1VFB.pdb_CL129A**	-1	0.172	-1	-1	--	--	--	-1	-1	--	--	-1	--	-1	-1	-1
**1VFB.pdb_CN19A**	-1	0.396	-1	-1	--	--	--	-1	-1	--	--	-1	--	-1	-1	-1
**1VFB.pdb_CQ121A**	1	2.878	1	1	--	--	--	1	1	--	--	1	--	1	1	1
**1VFB.pdb_CR125A**	-1	1.838	-1	-1	--	--	--	-1	-1	--	--	1	--	-1	-1	-1
**1VFB.pdb_CS24A**	-1	0.851	-1	-1	--	--	--	-1	-1	--	--	-1	--	-1	-1	-1
**1VFB.pdb_CT118A**	-1	0.765	-1	-1	--	--	--	-1	-1	--	--	-1	--	-1	-1	-1

Supplementary Text S4: Hotspot Predictor Predictions and Performance

**1VFB.pdb_CV120A**	-1	0.917	-1	-1	--	--	--	--	--	--	--	-1	--	-1	-1	-1
**1VFB.pdb_CY23A**	-1	0.411	-1	-1	--	--	--	--	--	--	--	-1	--	-1	-1	-1
**1XD3.pdb_BD39A**	-1	-0.411	-1	-1	-1	-1	-1	-1	-1	--	--	--	--	--	--	--
**1XD3.pdb_BD52A**	-1	-0.240	-1	-1	-1	-1	--	--	--	--	--	--	--	--	--	--
**1XD3.pdb_BI44A**	-1	0.266	-1	-1	-1	-1	--	--	--	--	--	--	--	--	--	--
**1XD3.pdb_BK27A**	-1	-0.062	-1	-1	-1	-1	--	--	--	--	--	--	--	--	--	--
**1XD3.pdb_BL8A**	1	2.677	1	1	1	1	1	-1	1	--	--	--	--	--	--	--
**1Z7X.pdb_WD435A**	1	3.662	-1	-1	1	-1	-1	1	1	--	--	--	--	--	--	--
**1Z7X.pdb_WE206A**	-1	1.019	-1	-1	-1	-1	--	--	--	--	--	--	--	--	--	--
**1Z7X.pdb_WE287A**	-1	1.321	-1	-1	-1	-1	1	-1	-1	--	--	--	--	--	--	--
**1Z7X.pdb_WE344A**	-1	1.562	-1	-1	-1	-1	--	--	--	--	--	--	--	--	--	--
**1Z7X.pdb_WE401A**	-1	1.306	-1	-1	-1	-1	-1	-1	-1	--	--	--	--	--	--	--
**1Z7X.pdb_WI459A**	-1	0.337	-1	-1	-1	-1	1	-1	-1	--	--	--	--	--	--	--
**1Z7X.pdb_WK320A**	-1	1.321	-1	-1	-1	-1	-1	-1	-1	--	--	--	--	--	--	--
**1Z7X.pdb_WR457A**	-1	0.848	-1	-1	-1	-1	-1	-1	-1	--	--	--	--	--	--	--
**1Z7X.pdb_WS289A**	-1	0.814	-1	-1	-1	-1	--	--	--	--	--	--	--	--	--	--
**1Z7X.pdb_WW261A**	-1	1.336	1	-1	1	-1	1	1	1	--	--	--	--	--	--	--
**1Z7X.pdb_WW263A**	1	2.212	-1	-1	-1	-1	1	-1	-1	--	--	--	--	--	--	--
**1Z7X.pdb_WW318A**	-1	0.993	-1	-1	-1	-1	1	-1	-1	--	--	--	--	--	--	--
**1Z7X.pdb_WW375A**	-1	1.670	-1	-1	-1	-1	1	-1	-1	--	--	--	--	--	--	--
**1Z7X.pdb_WY434A**	1	5.956	1	1	1	1	1	1	1	--	--	--	--	--	--	--
**1Z7X.pdb_WY437A**	1	2.624	1	1	1	1	1	-1	1	--	--	--	--	--	--	--
**2B42.pdb_AH374A**	-1	1.638	1	1	1	1	1	-1	-1	--	--	--	--	--	--	--
**2BTF.pdb_PF59A**	-1	1.596	-1	-1	-1	-1	-1	-1	1	--	--	--	--	--	--	--
**2BTF.pdb_PK125A**	-1	0.460	-1	-1	1	-1	-1	-1	-1	--	--	--	--	--	--	--
**2FTL.pdb_IG11A**	1	4.393	-1	-1	-1	-1	-1	-1	-1	--	--	--	--	--	--	--
**2FTL.pdb_IG35A**	1	2.214	-1	-1	-1	-1	1	-1	-1	--	--	--	--	--	--	--
**2FTL.pdb_II17A**	1	5.022	1	-1	1	1	1	-1	-1	--	--	--	--	--	--	--
**2FTL.pdb_IK14A**	1	10.159	1	-1	1	1	1	1	1	--	--	--	--	--	--	--
**2G2U.pdb_BE31A**	-1	0.651	-1	-1	-1	-1	-1	-1	1	--	--	--	--	--	--	--
**2G2U.pdb_BE73A**	-1	-1.979	-1	-1	1	1	1	-1	-1	--	--	--	--	--	--	--
**2G2U.pdb_BF142A**	-1	0.276	1	1	1	1	1	-1	-1	--	--	--	--	--	--	--
**2G2U.pdb_BF36A**	1	2.764	1	1	1	1	1	1	1	--	--	--	--	--	--	--
**2G2U.pdb_BG141A**	-1	-0.414	-1	-1	-1	-1	-1	-1	-1	--	--	--	--	--	--	--
**2G2U.pdb_BG48A**	-1	-0.427	-1	-1	-1	-1	1	1	1	--	--	--	--	--	--	--
**2G2U.pdb_BH148A**	-1	1.119	-1	-1	1	1	1	1	1	--	--	--	--	--	--	--
**2G2U.pdb_BH41A**	-1	1.717	-1	-1	1	1	1	-1	-1	--	--	--	--	--	--	--
**2G2U.pdb_BK74A**	-1	-0.217	-1	-1	1	1	-1	-1	-1	--	--	--	--	--	--	--
**2G2U.pdb_BR144A**	-1	-0.342	-1	-1	-1	-1	--	--	--	--	--	--	--	--	--	--
**2G2U.pdb_BR160A**	-1	0.670	-1	-1	-1	-1	-1	-1	-1	--	--	--	--	--	--	--
**2G2U.pdb_BS113A**	-1	-0.612	-1	-1	-1	-1	1	-1	-1	--	--	--	--	--	--	--
**2G2U.pdb_BS35A**	-1	-0.951	-1	-1	-1	-1	-1	-1	-1	--	--	--	--	--	--	--
**2G2U.pdb_BS39A**	-1	-0.956	-1	-1	-1	-1	-1	-1	-1	--	--	--	--	--	--	--
**2G2U.pdb_BS71A**	-1	-0.512	-1	-1	-1	-1	-1	-1	-1	--	--	--	--	--	--	--
**2G2U.pdb_BW112A**	-1	0.959	1	1	1	1	1	1	1	--	--	--	--	--	--	--
**2G2U.pdb_BW150A**	-1	1.785	-1	-1	-1	-1	-1	-1	-1	--	--	--	--	--	--	--
**2G2U.pdb_BW162A**	-1	0.531	-1	-1	-1	-1	--	--	--	--	--	--	--	--	--	--
**2G2U.pdb_BY143A**	-1	-1.847	-1	1	-1	-1	1	-1	-1	--	--	--	--	--	--	--
**2G2U.pdb_BY50A**	-1	-2.076	1	1	1	1	1	-1	-1	--	--	--	--	--	--	--
**2G2U.pdb_BY51A**	-1	-0.629	-1	-1	-1	-1	--	--	--	--	--	--	--	--	--	--
**2G2U.pdb_BY53A**	1	2.302	1	-1	1	1	-1	1	1	--	--	--	--	--	--	--
**2GOX.pdb_BN138A**	-1	1.570	-1	-1	1	-1	1	-1	-1	--	--	--	--	--	--	--
**2GOX.pdb_BR131A**	1	2.247	-1	-1	1	1	-1	-1	-1	--	--	--	--	--	--	--
**2HRK.pdb_AK159A**	-1	0.954	-1	-1	-1	-1	-1	-1	-1	--	--	--	--	--	--	--
**2I9B.pdb_EK139A**	-1	0.674	-1	-1	-1	-1	-1	-1	-1	--	--	--	--	--	--	--
**2I9B.pdb_ER137A**	-1	-0.288	-1	-1	1	1	-1	-1	1	--	--	--	--	--	--	--
**2J0T.pdb_DM66A**	-1	1.643	-1	-1	-1	-1	-1	-1	-1	--	--	--	--	--	--	--
**2J0T.pdb_DS68A**	1	2.106	-1	-1	1	-1	-1	-1	-1	--	--	--	--	--	--	--
**2J0T.pdb_DT2A**	1	4.289	1	-1	1	-1	1	1	1	--	--	--	--	--	--	--
**2J0T.pdb_DV4A**	-1	0.000	-1	-1	-1	-1	-1	-1	1	--	--	--	--	--	--	--
**2J1K.pdb_CR384A**	-1	0.765	-1	-1	-1	-1	1	-1	-1	--	--	--	--	--	--	--
**2JEL.pdb_PE68A**	-1	0.411	-1	-1	-1	-1	--	--	--	--	--	--	--	--	--	--
**2JEL.pdb_PE70A**	1	2.728	-1	-1	1	1	-1	-1	1	--	--	--	--	--	--	--
**2JEL.pdb_PH76A**	-1	-0.411	-1	-1	-1	-1	-1	-1	-1	--	--	--	--	--	--	--
**2JEL.pdb_PT62A**	-1	0.000	-1	-1	-1	-1	--	--	--	--	--	--	--	--	--	--
**2O3B.pdb_BE24A**	1	5.475	-1	-1	1	1	1	1	1	--	--	--	--	--	--	--
**2O3B.pdb_BQ74A**	1	3.233	-1	-1	-1	-1	--	--	--	--	--	--	--	--	--	--
**2O3B.pdb_BW76A**	1	4.074	1	1	1	-1	-1	-1	1	--	--	--	--	--	--	--
**2PCC.pdb_AD34A**	-1	-0.897	-1	-1	-1	-1	-1	-1	-1	--	--	--	--	--	--	--
**2PCC.pdb_AE290A**	1	6.203	-1	-1	-1	-1	-1	-1	-1	--	--	--	--	--	--	--
**2PCC.pdb_AV197A**	1	2.103	-1	-1	-1	-1	-1	-1	1	--	--	--	--	--	--	--
**2PCC.pdb_BK72A**	-1	0.304	-1	-1	-1	-1	-1	-1	-1	--	--	--	--	--	--	--
**2PCC.pdb_BK87A**	-1	0.902	-1	-1	-1	-1	-1	-1	-1	--	--	--	--	--	--	--
**2SIC.pdb_IM73A**	-1	0.218	1	1	1	1	1	-1	1	--	--	--	--	--	--	--
**2VLJ.pdb_DS31A**	-1	0.628	-1	-1	-1	-1	--	--	--	--	--	--	--	--	--	--
**2VLJ.pdb_DS32A**	-1	1.038	-1	-1	-1	-1	--	--	--	--	--	--	--	--	--	--
**2VLJ.pdb_ED32A**	-1	1.573	-1	-1	-1	-1	-1	-1	-1	--	--	--	--	--	--	--
**2VLJ.pdb_ED56A**	-1	0.132	-1	-1	-1	-1	--	--	--	--	--	--	--	--	--	--

2VLJ.pdb_EN55A	-1	1.130	-1	-1	-1	-1	--	--	--	--	--	--	--	--	--	--
2VLJ.pdb_EQ58A	-1	0.495	-1	-1	-1	-1	--	--	--	--	--	--	--	--	--	--
2VLJ.pdb_ES99A	-1	-0.035	-1	-1	1	-1	--	--	--	--	--	--	--	--	--	--
2VLJ.pdb_EY101A	-1	0.233	-1	-1	-1	-1	1	-1	1	--	--	--	--	--	--	--
2WPT.pdb_AD33A	-1	-0.132	-1	-1	1	-1	--	--	--	--	--	--	--	--	--	--
2WPT.pdb_AE30A	-1	1.734	-1	-1	1	-1	-1	-1	-1	--	--	--	--	--	--	--
2WPT.pdb_AE41A	1	4.503	-1	-1	-1	-1	-1	-1	-1	--	--	--	--	--	--	--
2WPT.pdb_AN34A	-1	-0.380	-1	-1	1	-1	-1	1	1	--	--	--	--	--	--	--
2WPT.pdb_AP56A	1	2.928	-1	-1	-1	-1	--	--	--	--	--	--	--	--	--	--
2WPT.pdb_AR38A	-1	-1.111	-1	-1	-1	-1	--	--	--	--	--	--	--	--	--	--
2WPT.pdb_AS50A	1	2.426	1	-1	1	-1	1	-1	1	--	--	--	--	--	--	--
2WPT.pdb_AV37A	1	3.809	-1	-1	1	-1	1	-1	1	--	--	--	--	--	--	--
2WPT.pdb_BF86A	-1	1.170	1	1	1	1	1	1	1	--	--	--	--	--	--	--
2WPT.pdb_BK97A	-1	0.497	1	1	1	1	-1	1	1	--	--	--	--	--	--	--
2WPT.pdb_BN72A	-1	0.918	-1	1	1	-1	-1	-1	-1	--	--	--	--	--	--	--
2WPT.pdb_BN75A	-1	1.238	1	-1	1	1	-1	-1	-1	--	--	--	--	--	--	--
2WPT.pdb_BQ92A	-1	0.900	-1	-1	-1	-1	1	-1	-1	--	--	--	--	--	--	--
2WPT.pdb_BR54A	-1	1.134	-1	-1	-1	-1	-1	-1	1	--	--	--	--	--	--	--
2WPT.pdb_BS74A	-1	-0.849	-1	-1	1	-1	-1	-1	-1	--	--	--	--	--	--	--
2WPT.pdb_BS77A	-1	-0.612	-1	-1	-1	-1	-1	-1	-1	--	--	--	--	--	--	--
2WPT.pdb_BS78A	-1	-0.150	-1	-1	-1	-1	1	-1	-1	--	--	--	--	--	--	--
2WPT.pdb_BS84A	-1	-0.094	-1	-1	-1	-1	-1	-1	-1	--	--	--	--	--	--	--
2WPT.pdb_BT87A	-1	0.526	-1	-1	-1	-1	1	-1	-1	--	--	--	--	--	--	--
2WPT.pdb_BV98A	-1	0.119	-1	-1	-1	-1	--	--	--	--	--	--	--	--	--	--
3BK3.pdb_CI17A	-1	0.486	-1	-1	-1	-1	1	-1	-1	--	--	--	--	--	--	--
3BK3.pdb_CI1A	-1	1.039	-1	-1	1	-1	1	-1	-1	--	--	--	--	--	--	--
3BK3.pdb_CI20A	-1	1.308	1	-1	1	-1	1	1	1	--	--	--	--	--	--	--
3BK3.pdb_CI26A	-1	1.262	-1	-1	-1	-1	1	-1	-1	--	--	--	--	--	--	--
3BK3.pdb_CL0A	-1	0.000	-1	-1	-1	-1	-1	-1	-1	--	--	--	--	--	--	--
3BN9.pdb_BD230A	-1	0.566	-1	1	1	-1	-1	-1	1	--	--	--	--	--	--	--
3BN9.pdb_BD61A	-1	0.423	-1	-1	-1	-1	--	--	--	--	--	--	--	--	--	--
3BN9.pdb_BD62A	-1	0.311	-1	1	-1	-1	-1	-1	-1	--	--	--	--	--	--	--
3BN9.pdb_BF104A	-1	0.640	-1	-1	-1	-1	--	--	--	--	--	--	--	--	--	--
3BN9.pdb_BH153A	-1	0.085	-1	-1	-1	-1	--	--	--	--	--	--	--	--	--	--
3BN9.pdb_BI41A	-1	0.000	-1	-1	-1	-1	--	--	--	--	--	--	--	--	--	--
3BN9.pdb_BI60A	-1	0.836	-1	-1	-1	-1	-1	-1	-1	--	--	--	--	--	--	--
3BN9.pdb_BK238A	-1	0.785	-1	-1	-1	-1	--	--	--	--	--	--	--	--	--	--
3BN9.pdb_BN105A	-1	0.774	-1	-1	-1	-1	-1	-1	-1	--	--	--	--	--	--	--
3BN9.pdb_BQ184A	-1	-0.035	-1	-1	-1	-1	-1	-1	-1	--	--	--	--	--	--	--
3BN9.pdb_BQ185A	1	2.510	-1	-1	1	-1	1	-1	-1	--	--	--	--	--	--	--
3BN9.pdb_BQ235A	-1	0.706	-1	-1	-1	-1	--	--	--	--	--	--	--	--	--	--
3BN9.pdb_BR63A	-1	-0.045	-1	-1	-1	-1	-1	-1	1	--	--	--	--	--	--	--
3BN9.pdb_BT108A	-1	1.133	-1	-1	-1	-1	-1	-1	-1	--	--	--	--	--	--	--
3BN9.pdb_BY156A	-1	1.085	-1	-1	-1	-1	--	--	--	--	--	--	--	--	--	--
3BP8.pdb_AF136A	-1	0.709	1	-1	1	1	--	--	--	--	--	--	--	--	--	--
3HFM.pdb_HD32A	-1	1.899	-1	-1	--	--	--	-1	-1	-1	-1	--	-1	-1	-1	-1
3HFM.pdb_HS31A	-1	0.170	-1	-1	--	--	--	-1	-1	-1	-1	--	-1	-1	-1	-1
3HFM.pdb_HW98A	1	5.513	1	1	1	1	1	-1	1	--	--	--	--	--	--	--
3HFM.pdb_HY33A	1	6.038	1	1	--	--	--	-1	1	1	1	--	1	1	1	1
3HFM.pdb_HY50A	1	7.323	1	-1	--	--	--	1	1	-1	-1	--	1	-1	-1	1
3HFM.pdb_HY53A	1	3.198	-1	-1	--	--	--	-1	-1	-1	-1	--	1	1	1	1
3HFM.pdb_HY58A	-1	1.649	-1	-1	--	--	--	-1	-1	-1	-1	--	--	-1	1	1
3HFM.pdb_LN31A	1	5.216	-1	-1	--	--	--	-1	-1	-1	-1	--	-1	1	1	1
3HFM.pdb_LN32A	1	5.110	1	-1	--	--	--	1	1	-1	-1	--	-1	1	-1	-1
3HFM.pdb_LQ53A	-1	0.954	-1	-1	--	--	--	-1	-1	-1	-1	--	--	-1	-1	-1
3HFM.pdb_LY50A	1	4.560	-1	-1	--	--	--	1	1	-1	-1	--	1	-1	1	1
3HFM.pdb_LY96A	1	2.708	-1	-1	--	--	--	-1	-1	-1	-1	--	-1	-1	-1	-1
3HFM.pdb_YD101A	-1	1.209	-1	-1	--	--	--	-1	1	1	1	-1	--	1	1	1
3HFM.pdb_YH15A	-1	-0.446	-1	-1	--	--	--	-1	-1	-1	-1	-1	1	-1	-1	-1
3HFM.pdb_YK96A	1	6.991	1	-1	--	--	--	-1	1	-1	1	1	1	1	1	1
3HFM.pdb_YK97A	1	6.170	1	1	--	--	--	-1	1	1	1	-1	-1	1	-1	1
3HFM.pdb_YL75A	-1	0.705	-1	-1	--	--	--	-1	1	-1	-1	-1	--	-1	-1	-1
3HFM.pdb_YN93A	-1	0.211	-1	-1	--	--	--	-1	-1	-1	-1	-1	--	-1	-1	-1
3HFM.pdb_YR21A	-1	1.028	-1	-1	--	--	--	-1	-1	1	1	1	--	-1	1	1
3HFM.pdb_YR73A	-1	-0.332	-1	-1	--	--	--	-1	-1	-1	-1	-1	-1	-1	-1	-1
3HFM.pdb_YS100A	-1	0.268	-1	-1	--	--	--	-1	-1	1	1	-1	-1	1	1	1
3HFM.pdb_YT89A	-1	0.000	-1	-1	--	--	--	-1	-1	-1	-1	-1	-1	-1	-1	-1
3HFM.pdb_YW63A	-1	0.319	-1	-1	--	--	--	--	--	-1	-1	-1	1	-1	-1	-1
3HFM.pdb_YY20A	1	4.878	1	1	--	--	--	1	1	1	1	1	1	1	1	1
3NPS.pdb_AD106A	-1	1.508	-1	-1	-1	-1	1	-1	-1	--	--	--	--	--	--	--
3NPS.pdb_AD230A	-1	1.467	1	1	1	-1	1	1	1	--	--	--	--	--	--	--
3NPS.pdb_AD61A	-1	0.340	-1	-1	-1	-1	--	--	--	--	--	--	--	--	--	--
3NPS.pdb_AD62A	-1	1.068	-1	-1	-1	-1	--	--	--	--	--	--	--	--	--	--
3NPS.pdb_AF107A	-1	0.464	-1	-1	1	-1	1	-1	1	--	--	--	--	--	--	--
3NPS.pdb_AH153A	-1	1.876	-1	-1	1	-1	-1	-1	-1	--	--	--	--	--	--	--
3NPS.pdb_AI41A	-1	0.642	-1	-1	-1	-1	--	--	--	--	--	--	--	--	--	--
3NPS.pdb_AI60A	-1	-0.332	-1	-1	-1	-1	--	--	--	--	--	--	--	--	--	--
3NPS.pdb_AQ184A	-1	-0.059	-1	-1	-1	-1	--	--	--	--	--	--	--	--	--	--

**3NPS.pdb_AQ185A**	-1	0.741	-1	-1	-1	-1	--	--	--	--	--	--	--	--	--	--
**3NPS.pdb_AQ235A**	-1	-0.041	-1	-1	-1	-1	--	--	--	--	--	--	--	--	--	--
**3NPS.pdb_AR63A**	-1	-1.063	-1	-1	-1	-1	-1	-1	1	--	--	--	--	--	--	--
**3NPS.pdb_AT108A**	-1	0.724	-1	-1	-1	-1	-1	-1	-1	--	--	--	--	--	--	--
**3NPS.pdb_AT160A**	-1	0.175	-1	-1	-1	-1	--	--	--	--	--	--	--	--	--	--
**3NPS.pdb_AY156A**	-1	1.776	-1	-1	-1	-1	-1	-1	-1	--	--	--	--	--	--	--
**3NPS.pdb_AY67A**	-1	0.452	-1	-1	-1	-1	--	--	--	--	--	--	--	--	--	--
**3SGB.pdb_IE19A**	-1	1.019	-1	-1	1	-1	-1	-1	-1	--	--	--	--	--	--	--
**3SGB.pdb_IG32A**	-1	1.223	-1	-1	-1	-1	-1	-1	-1	--	--	--	--	--	--	--
**3SGB.pdb_IK13A**	-1	-2.572	-1	-1	-1	-1	-1	-1	-1	--	--	--	--	--	--	--
**3SGB.pdb_IL18A**	1	2.989	-1	-1	1	1	1	1	1	--	--	--	--	--	--	--
**3SGB.pdb_IN36A**	-1	0.331	-1	-1	-1	-1	-1	-1	-1	--	--	--	--	--	--	--
**3SGB.pdb_IP14A**	-1	-0.189	-1	-1	-1	-1	-1	-1	-1	--	--	--	--	--	--	--
**3SGB.pdb_IR21A**	-1	0.054	-1	-1	-1	-1	-1	-1	1	--	--	--	--	--	--	--
**3SGB.pdb_IT17A**	1	3.592	1	-1	1	-1	-1	-1	1	--	--	--	--	--	--	--
**3SGB.pdb_IY20A**	-1	1.944	1	1	1	1	1	1	1	--	--	--	--	--	--	--
**4CPA.pdb_IV38A**	1	2.326	1	-1	1	-1	1	-1	-1	--	--	--	--	--	--	--

* Indicate Hotspot Predictors used for the generation of hotspot descriptors and further analyzed in main manuscript for off-rate prediction.

References:

1. Zhu X, Mitchell JC (2011) KFC2: a knowledge-based hot spot prediction method based on interface solvation, atomic density, and plasticity features. Proteins 79: 2671-2683.
2. Kortemme T, Baker D (2002) A simple physical model for binding energy hot spots in protein-protein complexes. Proc Natl Acad Sci U S A 99: 14116-14121.
3. Tuncbag N, Gursoy A, Keskin O (2009) Identification of computational hot spots in protein interfaces: combining solvent accessibility and inter-residue potentials improves the accuracy. Bioinformatics 25: 1513-1520.
4. Wang L, Liu ZP, Zhang XS, Chen L (2012) Prediction of hot spots in protein interfaces using a random forest model with hybrid features. Protein Eng Des Sel 25: 119-126.
5. Lise S, Archambeau C, Pontil M, Jones DT (2009) Prediction of hot spot residues at protein-protein interfaces by combining machine learning and energy-based methods. BMC Bioinformatics 10: 365.
