# Supplementary material for: Characterizing Changes in the Rate of Protein-Protein Dissociation upon Interface Mutation Using Hotspot Energy and Organization
Source: PLoS Comput Biol. 2013 Sep 5;9(9):e1003216. doi: 10.1371/journal.pcbi.1003216 (PMC3764008; doi:10.1371/journal.pcbi.1003216)
Supplement: Text S5 — Descriptor performance measures (PCC_UTest_AUC). (PDF) [file pcbi.1003216.s015.pdf]

Supplementary Text S5: Descriptor Performance Measures (PCC / U-Test /AUC)

Table S6. Ranked absolute PCCs of hotspot and molecular descriptors with experimental Δ$log_{10}(k_{off})$. Experimental data for $log_{10}(k_{off})$ is based on the 713 kinetically characterized mutations from SKEMPI. Descriptor Categories: AP – Atomic-based Statistical Potential, CP – Coarse-Grain Statistical Potential, PB – Physics-based Energy Term. For Hotspot Descriptors, Descriptor Categories shows hotspot prediction algorithm generating the respective hotspot predictor.

Rank	Descriptor	Descriptor Category	Pearson's Correlation Coefficient	Ranked Absolute Pearson's Correlation Coefficient	Descriptor Type
1	SuppHSEnergy	KFC2a	-0.617	0.617	Hotspot
2	CoreHSEnergy	Hotpoint1	-0.608	0.608	Hotspot
3	HSEner_PosCoop	RFSpot	-0.573	0.573	Hotspot
4	Int_HS_Energy	Hotpoint1	-0.568	0.568	Hotspot
5	CoreHSEnergy	RFSpot_KFC2	-0.560	0.560	Hotspot
6	Int_HS_Energy	RFSpot_KFC2	-0.559	0.559	Hotspot
7	Int_HS_Energy	KFC2a	-0.546	0.546	Hotspot
8	CoreHSEnergy	RFSpot	-0.541	0.541	Hotspot
9	Int_HS_Energy	RFSpot	-0.532	0.532	Hotspot
10	Int_HS_Energy	KFC2b	-0.527	0.527	Hotspot
11	HSEner_NegCoop	RFSpot_KFC2	-0.514	0.514	Hotspot
12	No_HS	KFC2b	-0.496	0.496	Hotspot
13	No_HS	RFSpot_KFC2	-0.496	0.496	Hotspot
14	HS_PosCoop	RFSpot	-0.494	0.494	Hotspot
15	No_HS	RFSpot	-0.493	0.493	Hotspot
16	SuppHSEnergy	KFC2b	-0.489	0.489	Hotspot
17	HSEner_NegCoop	Hotpoint1	-0.487	0.487	Hotspot
18	SuppHS	RFSpot	-0.480	0.480	Hotspot
19	CoreHS	KFC2b	-0.476	0.476	Hotspot
20	HSEner_NegCoop	KFC2a	-0.475	0.475	Hotspot
21	Int_Energy_1	KFC2a	-0.472	0.472	Hotspot
22	SuppHSEnergy	RFSpot_KFC2	-0.465	0.465	Hotspot
23	HS_NegCoop	RFSpot_KFC2	-0.460	0.460	Hotspot
24	HS_PosCoop	RFSpot_KFC2	-0.457	0.457	Hotspot
25	AP_MPS	AP	0.456	0.456	Molecular
26	HSEner_PosCoop	RFSpot_KFC2	-0.444	0.444	Hotspot
27	SuppHS	RFSpot_KFC2	-0.441	0.441	Hotspot
28	CoreHS	RFSpot_KFC2	-0.440	0.440	Hotspot
29	HSEner_PosCoop	KFC2b	-0.437	0.437	Hotspot
30	No_HS	Hotpoint1	-0.433	0.433	Hotspot
31	Int_Energy_1	KFC2b	-0.432	0.432	Hotspot
32	SuppHS	KFC2a	-0.430	0.430	Hotspot
33	No_HS	KFC2a	-0.429	0.429	Hotspot
34	CoreHSEnergy	KFC2b	-0.427	0.427	Hotspot
35	CP_SKOa	CP	0.415	0.415	Molecular
36	RimHSEnergy	Hotpoint1	-0.415	0.415	Hotspot
37	HS_NegCoop	RFSpot	-0.415	0.415	Hotspot
38	HSEner_NegCoop	RFSpot	-0.414	0.414	Hotspot
39	CoreHS	Hotpoint1	-0.413	0.413	Hotspot
40	CP_SKOb	CP	0.391	0.391	Molecular
41	SuppHSEnergy	RFSpot	-0.385	0.385	Hotspot
42	CP_Qp	CP	0.380	0.380	Molecular
43	CP_TB	CP	0.378	0.378	Molecular
44	CHARM_gb	PB	-0.373	0.373	Molecular
45	CoreHSEnergy	KFC2a	-0.369	0.369	Hotspot
46	RimHSEnergy	RFSpot	-0.367	0.367	Hotspot
47	CoreHSEnergy	Hotpoint2	-0.365	0.365	Hotspot
48	HS_NegCoop	KFC2b	-0.356	0.356	Hotspot
49	CHARM_elec	PB	0.353	0.353	Molecular
50	SuppHS	KFC2b	-0.344	0.344	Hotspot
51	CoreHS	RFSpot	-0.342	0.342	Hotspot
52	CP_BT	CP	0.337	0.337	Molecular
53	HS_NegCoop	Hotpoint1	-0.330	0.330	Hotspot
54	CP_BFKV	CP	0.330	0.330	Molecular
55	RimHSEnergy	RFSpot_KFC2	-0.329	0.329	Hotspot
56	RimHS	Hotpoint1	-0.319	0.319	Hotspot
57	Int_Energy_1	Hotpoint1	-0.312	0.312	Hotspot
58	Int_Energy_1	Hotpoint2	-0.312	0.312	Hotspot
59	Int_HS_Energy	Hotpoint2	-0.312	0.312	Hotspot
60	CP_MJ2	CP	0.302	0.302	Molecular
61	CP_RO	CP	-0.297	0.297	Molecular
62	CP_BL	CP	0.296	0.296	Molecular
63	GEOMETRIC	CP	0.295	0.295	Molecular
64	MaxClusterSize	RFSpot_KFC2	-0.292	0.292	Hotspot
65	CP_Qm	CP	0.291	0.291	Molecular
66	Int_Energy_1	RFSpot_KFC2	-0.289	0.289	Hotspot
67	No_Clusters	RFSpot_KFC2	-0.285	0.285	Hotspot
68	No_Clusters	RFSpot	-0.284	0.284	Hotspot
69	HSEner_NegCoop	Hotpoint2	-0.282	0.282	Hotspot
70	CoreHS	Hotpoint2	-0.281	0.281	Hotspot
71	HSEner_PosCoop	Hotpoint1	-0.278	0.278	Hotspot
72	OPUS_PSP2	CP	0.274	0.274	Molecular
73	No_Clusters	KFC2b	-0.272	0.272	Hotspot
74	FX1_int_kn electrostatic	PB	0.268	0.268	Molecular
75	FX2_kn electrostatic	PB	0.268	0.268	Molecular
76	CP_SJKG	CP	0.266	0.266	Molecular
77	No_HS	Hotpoint2	-0.266	0.266	Hotspot
78	HSEner_NegCoop	KFC2b	-0.260	0.260	Hotspot
79	CP_TEI	CP	0.258	0.258	Molecular
80	HS_PosCoop	Hotpoint2	-0.256	0.256	Hotspot
81	PY_fa_atr	PB	0.249	0.249	Molecular
82	PY_fa_sol	PB	-0.243	0.243	Molecular

83	OPUS_PSP3	CP	-0.242	0.242	Molecular
84	CoreHS	KFC2a	-0.232	0.232	Hotspot
85	MaxClusterSize	Hotpoint1	-0.225	0.225	Hotspot
86	CP_GKS	CP	0.224	0.224	Molecular
87	HSEner_PosCoop	KFC2a	-0.218	0.218	Hotspot
88	CP_RMFCA	CP	0.217	0.217	Molecular
89	CP_Qa	CP	0.197	0.197	Molecular
90	AVG_HS_PathLength	KFC2b	-0.197	0.197	Hotspot
91	FX1_int_Entropy_sidec	PB	-0.196	0.196	Molecular
92	HSEner_PosCoop	Hotpoint2	-0.192	0.192	Hotspot
93	FX1_int_Energy_VdW	PB	0.191	0.191	Molecular
94	FX2_Entropy_sidec	PB	-0.186	0.186	Molecular
95	FX2_Energy_VdW	PB	0.185	0.185	Molecular
96	ACE19_SCREENing	PB	0.185	0.185	Molecular
97	Int_Energy_1	RFSpot	-0.182	0.182	Hotspot
98	RimHS	KFC2a	-0.181	0.181	Hotspot
99	RimHS	RFSpot_KFC2	-0.176	0.176	Hotspot
100	CP_TD	CP	0.176	0.176	Molecular
101	MaxClusterSize	RFSpot	-0.163	0.163	Hotspot
102	SuppHSEnergy	Hotpoint2	-0.162	0.162	Hotspot
103	ACE19_INTERaction	PB	0.159	0.159	Molecular
104	SuppHS	Hotpoint1	-0.156	0.156	Hotspot
105	HS_PosCoop	KFC2b	-0.154	0.154	Hotspot
106	FX1_int_Energy_vdwclash	PB	-0.154	0.154	Molecular
107	SuppHSEnergy	Hotpoint1	-0.153	0.153	Hotspot
108	SuppHS	Hotpoint2	-0.153	0.153	Hotspot
109	FX2_Energy_vdwclash	PB	-0.152	0.152	Molecular
110	AVG_HS_PathLength	Hotpoint1	-0.152	0.152	Hotspot
111	DCOMPLEX	AP	0.150	0.150	Molecular
112	AVG_HS_PathLength	Hotpoint2	-0.139	0.139	Hotspot
113	CP_HLPL	CP	0.139	0.139	Molecular
114	CP_MJPL	CP	0.138	0.138	Molecular
115	HS_PosCoop	KFC2a	-0.138	0.138	Hotspot
116	AP_T1	AP	0.134	0.134	Molecular
117	CP_MS	CP	0.131	0.131	Molecular
118	RimHS	RFSpot	-0.128	0.128	Hotspot
119	CP_TS	CP	0.125	0.125	Molecular
120	CP_SKOIP	CP	0.125	0.125	Molecular
121	FX2_ helix dipole	PB	0.121	0.121	Molecular
122	FX1_int_Entropy_mainc	PB	-0.119	0.119	Molecular
123	FX1_int_Energy_SolvP	PB	-0.115	0.115	Molecular
124	FX2_Energy_SolvP	PB	-0.113	0.113	Molecular
125	FX2_Entropy_mainc	PB	-0.111	0.111	Molecular
126	AVG_HS_PathLength	RFSpot	-0.110	0.110	Hotspot
127	CP_TSC	CP	0.107	0.107	Molecular
128	FX1_int_ helix dipole	PB	0.107	0.107	Molecular
129	No_Clusters	Hotpoint1	0.101	0.101	Hotspot
130	RimHSEnergy	KFC2a	-0.100	0.100	Hotspot
131	AP_DOPE_HR	AP	-0.099	0.099	Molecular
132	PY_hbond_sc	PB	0.099	0.099	Molecular
133	FX1_int_backbone_vdwclash	PB	-0.097	0.097	Molecular
134	FX2_backbone_vdwclash	PB	-0.094	0.094	Molecular
135	MaxClusterSize	KFC2a	0.094	0.094	Hotspot
136	CP_RMFCEN1	CP	0.085	0.085	Molecular
137	AP_DOPE	AP	0.083	0.083	Molecular
138	FX2_Energy_Ionisation	PB	0.079	0.079	Molecular
139	FX1_int_energy_torsion	PB	-0.076	0.076	Molecular
140	No_Clusters	KFC2a	-0.075	0.075	Hotspot
141	RimHS	Hotpoint2	-0.071	0.071	Hotspot
142	FX1_int_Electro	PB	0.071	0.071	Molecular
143	No_Clusters	Hotpoint2	-0.069	0.069	Hotspot
144	ACE19_SELF	PB	-0.068	0.068	Molecular
145	PY_hbond_sr_bb	PB	-0.067	0.067	Molecular
146	AP_DARS	AP	0.067	0.067	Molecular
147	FX2_energy_torsion	PB	-0.066	0.066	Molecular
148	FX2_Electro	PB	0.065	0.065	Molecular
149	PY_fa_rep	PB	-0.060	0.060	Molecular
150	CP_MJ1	CP	-0.060	0.060	Molecular
151	CP_MJ3h	CP	0.054	0.054	Molecular
152	NIP	CP	0.052	0.052	Molecular
153	MaxClusterSize	KFC2b	0.052	0.052	Hotspot
154	CP_MJ2h	CP	0.050	0.050	Molecular
155	HS_NegCoop	KFC2a	-0.049	0.049	Hotspot
156	CHARM_total	PB	-0.049	0.049	Molecular
157	CHARM_vdwaals	PB	-0.049	0.049	Molecular
158	CP_TEs	CP	0.046	0.046	Molecular
159	FX1_int_Energy_Ionisation	PB	-0.044	0.044	Molecular
160	DDFIRE	AP	0.044	0.044	Molecular
161	FX2_Entropy Complex	PB	0.042	0.042	Molecular
162	PY_fa_dun	PB	0.042	0.042	Molecular
163	FX1_int_Entropy Complex	PB	0.042	0.042	Molecular
164	ACE19_SOLVation	PB	0.040	0.040	Molecular
165	CP_RMFCEN2	CP	0.040	0.040	Molecular
166	CP_MSBM	CP	0.040	0.040	Molecular
167	PY_pro_close	PB	0.038	0.038	Molecular
168	ACE19_HYDRophobic	PB	-0.038	0.038	Molecular
169	AP_URS	AP	0.037	0.037	Molecular
170	FX2_disulfide	PB	0.035	0.035	Molecular

171	CP_VD	CP	0.035	0.035	Molecular
172	FX1_int_disulfide	PB	0.034	0.034	Molecular
173	FX1_int_partial covalent interactions	PB	-0.034	0.034	Molecular
174	FX1_int_water bonds	PB	-0.033	0.033	Molecular
175	AVG_HS_PathLength	KFC2a	-0.031	0.031	Hotspot
176	FX2_water bonds	PB	-0.031	0.031	Molecular
177	FX2_loop_entropy	PB	-0.030	0.030	Molecular
178	FX1_int_SideHbond	PB	-0.030	0.030	Molecular
179	FX2_partial covalent interactions	PB	0.030	0.030	Molecular
180	DFIRE	AP	0.027	0.027	Molecular
181	FX2_SideHbond	PB	-0.025	0.025	Molecular
182	PY_fa_intra_rep	PB	-0.023	0.023	Molecular
183	CHARM_gb+sasa	PB	-0.022	0.022	Molecular
184	NSc	CP	-0.022	0.022	Molecular
185	PY_hbond_lr_bb	PB	-0.022	0.022	Molecular
186	MaxClusterSize	Hotpoint2	0.022	0.022	Hotspot
187	ACE19_SASL	PB	-0.022	0.022	Molecular
188	RimHSEnergy	Hotpoint2	0.020	0.020	Hotspot
189	PY_fa_pair	PB	0.018	0.018	Molecular
190	FX1_int_cis_bond	PB	-0.017	0.017	Molecular
191	CHARM_sasa	PB	-0.017	0.017	Molecular
192	AVG_HS_PathLength	RFSpot_KFC2	-0.016	0.016	Hotspot
193	FX2_cis_bond	PB	-0.016	0.016	Molecular
194	HS_NegCoop	Hotpoint2	0.013	0.013	Hotspot
195	PY_hbond_bb_sc	PB	0.013	0.013	Molecular
196	HS_PosCoop	Hotpoint1	-0.013	0.013	Hotspot
197	OPUS_PSP1	CP	-0.010	0.010	Molecular
198	FX2_Energy_SolvH	PB	0.003	0.003	Molecular
199	FX1_int_Energy_SolvH	PB	0.003	0.003	Molecular
200	FX1_int_BackHbond	PB	-0.001	0.001	Molecular
201	ACE19_COULomb	PB	0.000	0.000	Molecular
202	FX2_BackHbond	PB	0.000	0.000	Molecular
203	RimHSEnergy	KFC2b	0.000	0.000	Hotspot
204	RimHS	KFC2b	0.000	0.000	Hotspot
205	FX1_int_loop_entropy	PB	0.000	0.000	Molecular
206	AP_T2	AP	0.000	0.000	Molecular

Table S7. Mann Whitney U-Test of hotspot and molecular descriptors. Two different data sets are used. Left table uses experimental data from the kinetically characterized mutations in SKEMPI which satisfy Δlog10(koff) <-1 and Δlog10(koff) >0 (CDS1). Right table uses same experimental data but which satisfies |Δlog10(koff)| >1 (CDS2).

Δlog10(koff)<-1 and Δlog10(koff)>0 (CDS1)

Rank	Descriptor	Descriptor Category	Pval	$-\log_{10}$(pval)	Descriptor Type
1	HS_PosCoop	RFSpot	6.84E-10	9.16	Hotspot
2	HS_PosCoop	RFSpot_KFC2	9.65E-10	9.02	Hotspot
3	HSEner_PosCoop	RFSpot	1.21E-09	8.92	Hotspot
4	RimHSEnergy	KFC2a	2.08E-08	7.68	Hotspot
5	RimHS	KFC2a	5.78E-07	6.24	Hotspot
6	NSc	CP	3.68E-06	5.43	Molecular
7	AP_DOPE	AP	5.72E-06	5.24	Molecular
8	CP_TB	CP	8.33E-06	5.08	Molecular
9	AP_MPS	AP	1.22E-05	4.92	Molecular
10	HSEner_PosCoop	RFSpot_KFC2	1.55E-05	4.81	Hotspot
11	No_Clusters	RFSpot	1.75E-05	4.76	Hotspot
12	SuppHSEnergy	KFC2a	4.25E-05	4.37	Hotspot
13	No_HS	KFC2a	8.82E-05	4.05	Hotspot
14	No_HS	KFC2b	1.04E-04	3.99	Hotspot
15	DFIRE	AP	1.37E-04	3.86	Molecular
16	CHARM_gb	PB	1.70E-04	3.77	Molecular
17	Int_HS_Energy	RFSpot	1.91E-04	3.72	Hotspot
18	ACE19_HYDRophobic	PB	2.73E-04	3.56	Molecular
19	No_HS	Hotpoint2	2.85E-04	3.55	Hotspot
20	Int_HS_Energy	RFSpot_KFC2	2.91E-04	3.54	Hotspot
21	FX1_int_Energy_SolvH	PB	2.96E-04	3.53	Molecular
22	FX2_Energy_SolvH	PB	3.00E-04	3.52	Molecular
23	CoreHSEnergy	RFSpot	3.01E-04	3.52	Hotspot
24	CP_SKOb	CP	3.60E-04	3.44	Molecular
25	CoreHSEnergy	RFSpot_KFC2	3.73E-04	3.43	Hotspot
26	CP_SKOa	CP	3.80E-04	3.42	Molecular
27	GEOMETRIC	CP	4.06E-04	3.39	Molecular
28	CP_RMFCA	CP	4.09E-04	3.39	Molecular
29	AP_T2	AP	4.34E-04	3.36	Molecular
30	CHARM_gb+sasa	PB	4.92E-04	3.31	Molecular
31	PY_fa_dun	PB	5.89E-04	3.23	Molecular
32	PY_fa_intra_rep	PB	5.98E-04	3.22	Molecular
33	No_Clusters	Hotpoint2	6.96E-04	3.16	Hotspot
34	DCOMPLEX	AP	7.53E-04	3.12	Molecular
35	HSEner_NegCoop	Hotpoint1	7.55E-04	3.12	Hotspot
36	FX1_int_Energy_VdW	PB	1.02E-03	2.99	Molecular
37	FX2_Energy_VdW	PB	1.22E-03	2.91	Molecular
38	CoreHSEnergy	Hotpoint1	1.31E-03	2.88	Hotspot
39	Int_Energy_1	KFC2b	1.46E-03	2.84	Hotspot
40	FX2_kn electrostatic	PB	1.72E-03	2.77	Molecular
41	CP_Qp	CP	1.83E-03	2.74	Molecular
42	FX1_int_kn electrostatic	PB	1.96E-03	2.71	Molecular
43	PY_fa_atr	PB	1.97E-03	2.71	Molecular
44	SuppHSEnergy	RFSpot_KFC2	2.12E-03	2.67	Hotspot
45	CoreHS	KFC2b	2.47E-03	2.61	Hotspot
46	ACE19_SCREENing	PB	2.65E-03	2.58	Molecular
47	DDFIRE	AP	2.80E-03	2.55	Molecular
48	RimHS	RFSpot	2.84E-03	2.55	Hotspot
49	ACE19_SELF	PB	2.95E-03	2.53	Molecular
50	PY_fa_pair	PB	2.97E-03	2.53	Molecular
51	Int_Energy_1	KFC2a	3.25E-03	2.49	Hotspot
52	ACE19_INTERaction	PB	3.51E-03	2.45	Molecular
53	FX1_int_Entropy_sidec	PB	3.71E-03	2.43	Molecular
54	Int_HS_Energy	Hotpoint1	3.83E-03	2.42	Hotspot
55	FX1_int_ helix dipole	PB	4.13E-03	2.38	Molecular
56	FX2_Entropy_sidec	PB	4.16E-03	2.38	Molecular
57	CP_HLPL	CP	4.65E-03	2.33	Molecular
58	CHARM_elec	PB	5.31E-03	2.27	Molecular
59	NIP	CP	6.30E-03	2.20	Molecular
60	MaxClusterSize	KFC2a	6.60E-03	2.18	Hotspot
61	OPUS_PSP2	CP	6.88E-03	2.16	Molecular
62	No_HS	RFSpot	6.96E-03	2.16	Hotspot
63	FX1_int_Energy_SolvP	PB	7.08E-03	2.15	Molecular

|Δlog10(koff)|>1 (CDS2).

Rank	Descriptor	Descriptor Category	Pval	$-\log_{10}$(pval)	Descriptor Type
1	HSEner_PosCoop	RFSpot	6.29E-16	15.20	Hotspot
2	HS_PosCoop	RFSpot_KFC2	3.22E-14	13.49	Hotspot
3	HS_PosCoop	RFSpot	7.26E-14	13.14	Hotspot
4	HSEner_PosCoop	RFSpot_KFC2	2.56E-13	12.59	Hotspot
5	Int_HS_Energy	RFSpot	9.93E-12	11.00	Hotspot
6	Int_HS_Energy	Hotpoint1	1.86E-11	10.73	Hotspot
7	CoreHSEnergy	RFSpot	2.08E-11	10.68	Hotspot
8	CoreHSEnergy	RFSpot_KFC2	2.60E-11	10.58	Hotspot
9	Int_HS_Energy	RFSpot_KFC2	2.88E-11	10.54	Hotspot
10	CoreHSEnergy	Hotpoint1	1.02E-10	9.99	Hotspot
11	SuppHSEnergy	KFC2a	2.64E-10	9.58	Hotspot
12	AP_MPS	2	3.46E-10	9.46	Molecular
13	SuppHSEnergy	RFSpot_KFC2	4.12E-10	9.38	Hotspot
14	HSEner_NegCoop	Hotpoint1	2.60E-09	8.59	Hotspot
15	No_HS	RFSpot	8.81E-09	8.06	Hotspot
16	CP_TB	1	1.03E-08	7.99	Molecular
17	ACE19_HYDRophobic	3	2.36E-08	7.63	Molecular
18	SuppHSEnergy	RFSpot	2.51E-08	7.60	Hotspot
19	No_HS	KFC2b	3.67E-08	7.44	Hotspot
20	HS_NegCoop	RFSpot	5.65E-08	7.25	Hotspot
21	No_HS	KFC2a	8.73E-08	7.06	Hotspot
22	FX1_int_Energy_VdW	3	9.67E-08	7.01	Molecular
23	RimHSEnergy	KFC2a	1.24E-07	6.90	Hotspot
24	SuppHSEnergy	KFC2b	1.33E-07	6.88	Hotspot
25	FX2_Energy_VdW	3	1.37E-07	6.86	Molecular
26	FX2_Energy_SolvH	3	2.37E-07	6.63	Molecular
27	FX1_int_Energy_SolvH	3	2.37E-07	6.63	Molecular
28	HS_NegCoop	RFSpot_KFC2	2.92E-07	6.53	Hotspot
29	HSEner_NegCoop	RFSpot	3.09E-07	6.51	Hotspot
30	CP_Qp	1	4.01E-07	6.40	Molecular
31	SuppHS	RFSpot	4.23E-07	6.37	Hotspot
32	PY_fa_atr	3	4.40E-07	6.36	Molecular
33	No_HS	Hotpoint1	5.92E-07	6.23	Hotspot
34	Int_HS_Energy	KFC2b	6.40E-07	6.19	Hotspot
35	CP_HLPL	1	6.56E-07	6.18	Molecular
36	No_Clusters	RFSpot	7.90E-07	6.10	Hotspot
37	CP_SKOb	1	1.01E-06	6.00	Molecular
38	CoreHS	KFC2b	1.68E-06	5.78	Hotspot
39	CP_SKOa	1	2.21E-06	5.66	Molecular
40	Int_Energy_1	KFC2b	2.37E-06	5.62	Hotspot
41	Int_Energy_1	KFC2a	2.37E-06	5.62	Hotspot
42	HSEner_NegCoop	RFSpot_KFC2	2.70E-06	5.57	Hotspot
43	AP_DOPE	2	2.79E-06	5.55	Molecular
44	CHARM_gb	3	5.00E-06	5.30	Molecular
45	ACE19_SCREENing	3	5.55E-06	5.26	Molecular
46	NSc	1	6.32E-06	5.20	Molecular
47	ACE19_SELF	3	8.57E-06	5.07	Molecular
48	Int_HS_Energy	KFC2a	9.79E-06	5.01	Hotspot
49	CHARM_gb+sasa	3	9.98E-06	5.00	Molecular
50	CP_RMFCA	1	1.15E-05	4.94	Molecular
51	RimHS	KFC2a	1.24E-05	4.91	Hotspot
52	ACE19_INTERaction	3	1.70E-05	4.77	Molecular
53	PY_fa_dun	3	1.72E-05	4.76	Molecular
54	FX1_int_Energy_SolvP	3	2.47E-05	4.61	Molecular
55	HS_NegCoop	Hotpoint1	3.12E-05	4.51	Hotspot
56	SuppHS	KFC2a	3.96E-05	4.40	Hotspot
57	No_HS	Hotpoint2	5.22E-05	4.28	Hotspot
58	GEOMETRIC	1	6.13E-05	4.21	Molecular
59	CoreHS	Hotpoint1	6.51E-05	4.19	Hotspot
60	FX2_Energy_SolvP	3	6.64E-05	4.18	Molecular
61	RimHSEnergy	Hotpoint1	7.69E-05	4.11	Hotspot
62	PY_fa_sol	3	7.80E-05	4.11	Molecular
63	FX2_backbone_vdwclash	3	8.97E-05	4.05	Molecular

64	CP_TEs	CP	8.64E-03	2.06	Molecular
65	SuppHSEnergy	KFC2b	9.87E-03	2.01	Hotspot
66	FX1_int_BackHbond	PB	9.93E-03	2.00	Molecular
67	No_HS	Hotpoint1	1.01E-02	2.00	Hotspot
68	FX2_Energy_SolvP	PB	1.05E-02	1.98	Molecular
69	HS_NegCoop	RFSpot	1.14E-02	1.94	Hotspot
70	RimHSEnergy	Hotpoint1	1.14E-02	1.94	Hotspot
71	CP_Qm	CP	1.24E-02	1.91	Molecular
72	SuppHS	KFC2a	1.28E-02	1.89	Hotspot
73	No_Clusters	RFSpot_KFC2	1.46E-02	1.83	Hotspot
74	FX2_backbone_vdwclash	PB	1.48E-02	1.83	Molecular
75	SuppHSEnergy	RFSpot	1.52E-02	1.82	Hotspot
76	PY_fa_sol	PB	1.62E-02	1.79	Molecular
77	AP_T1	AP	1.75E-02	1.76	Molecular
78	FX1_int_backbone_vdwclash	PB	1.79E-02	1.75	Molecular
79	No_Clusters	KFC2b	1.97E-02	1.71	Hotspot
80	Int_HS_Energy	KFC2a	2.00E-02	1.70	Hotspot
81	CoreHS	Hotpoint1	2.22E-02	1.65	Hotspot
82	OPUS_PSP1	CP	2.26E-02	1.65	Molecular
83	FX2_BackHbond	PB	2.49E-02	1.60	Molecular
84	SuppHS	KFC2b	2.57E-02	1.59	Hotspot
85	ACE19_SASL	PB	2.96E-02	1.53	Molecular
86	CP_MSBM	CP	3.00E-02	1.52	Molecular
87	CP_SJKG	CP	3.28E-02	1.48	Molecular
88	CP_BL	CP	3.33E-02	1.48	Molecular
89	RimHS	Hotpoint1	3.52E-02	1.45	Hotspot
90	HSEner_NegCoop	RFSpot	3.94E-02	1.40	Hotspot
91	Int_HS_Energy	KFC2b	3.95E-02	1.40	Hotspot
92	HS_NegCoop	RFSpot_KFC2	4.40E-02	1.36	Hotspot
93	CP_RO	CP	5.12E-02	1.29	Molecular
94	HSEner_NegCoop	KFC2a	5.71E-02	1.24	Hotspot
95	AVG_HS_PathLength	Hotpoint2	5.75E-02	1.24	Hotspot
96	HSEner_NegCoop	RFSpot_KFC2	6.29E-02	1.20	Hotspot
97	RimHS	RFSpot_KFC2	6.61E-02	1.18	Hotspot
98	HS_NegCoop	KFC2b	6.97E-02	1.16	Hotspot
99	CP_MJPL	CP	7.23E-02	1.14	Molecular
100	SuppHS	Hotpoint2	7.44E-02	1.13	Hotspot
101	HS_PosCoop	Hotpoint2	7.74E-02	1.11	Hotspot
102	HSEner_PosCoop	Hotpoint2	8.14E-02	1.09	Hotspot
103	CHARM_sasa	PB	9.55E-02	1.02	Molecular
104	PY_hbond_bb_sc	PB	9.59E-02	1.02	Molecular
105	SuppHS	RFSpot	9.70E-02	1.01	Hotspot
106	MaxClusterSize	Hotpoint1	1.03E-01	0.99	Hotspot
107	SuppHSEnergy	Hotpoint2	1.05E-01	0.98	Hotspot
108	CoreHS	KFC2a	1.08E-01	0.97	Hotspot
109	CP_SKOIP	CP	1.11E-01	0.96	Molecular
110	PY_hbond_sc	PB	1.14E-01	0.94	Molecular
111	CP_TD	CP	1.19E-01	0.92	Molecular
112	CP_TS	CP	1.21E-01	0.92	Molecular
113	MaxClusterSize	RFSpot	1.36E-01	0.87	Hotspot
114	RimHS	Hotpoint2	1.47E-01	0.83	Hotspot
115	HSEner_NegCoop	Hotpoint2	1.60E-01	0.80	Hotspot
116	CP_RMFCEN1	CP	1.69E-01	0.77	Molecular
117	AP_URS	AP	1.70E-01	0.77	Molecular
118	HSEner_PosCoop	KFC2b	1.83E-01	0.74	Hotspot
119	PY_pro_close	PB	1.94E-01	0.71	Molecular
120	FX2_ helix dipole	PB	2.14E-01	0.67	Molecular
121	CoreHS	Hotpoint2	2.35E-01	0.63	Hotspot
122	PY_fa_rep	PB	2.38E-01	0.62	Molecular
123	CoreHS	RFSpot	2.38E-01	0.62	Hotspot
124	FX1_int_energy_torsion	PB	2.50E-01	0.60	Molecular
125	HS_PosCoop	KFC2b	2.61E-01	0.58	Hotspot
126	FX2_energy_torsion	PB	2.74E-01	0.56	Molecular
127	RimHSEnergy	Hotpoint2	2.78E-01	0.56	Hotspot
128	AP_DARS	AP	2.97E-01	0.53	Molecular
129	CP_RMFCEN2	CP	3.00E-01	0.52	Molecular
130	MaxClusterSize	RFSpot_KFC2	3.07E-01	0.51	Hotspot
131	FX2_Energy_Ionisation	PB	3.07E-01	0.51	Molecular
132	FX1_int_Entropy_mainc	PB	3.17E-01	0.50	Molecular
133	CHARM_vdwaals	PB	3.19E-01	0.50	Molecular
134	FX1_int_disulfide	PB	3.21E-01	0.49	Molecular
135	FX1_int_Entropy Complex	PB	3.21E-01	0.49	Molecular
136	FX2_Entropy Complex	PB	3.21E-01	0.49	Molecular
137	CP_Qa	CP	3.35E-01	0.47	Molecular
138	HS_NegCoop	KFC2a	3.42E-01	0.47	Hotspot
139	RimHSEnergy	RFSpot_KFC2	3.47E-01	0.46	Hotspot
140	CP_TEI	CP	3.51E-01	0.45	Molecular
141	FX2_cis_bond	PB	3.66E-01	0.44	Molecular
142	HS_NegCoop	Hotpoint1	3.74E-01	0.43	Hotspot
143	CoreHSEnergy	Hotpoint2	3.77E-01	0.42	Hotspot
144	No_Clusters	KFC2a	3.89E-01	0.41	Hotspot
145	FX2_Entropy_mainc	PB	4.00E-01	0.40	Molecular
146	FX2_loop_entropy	PB	4.13E-01	0.38	Molecular
147	FX1_int_partial covalent interactions	PB	4.13E-01	0.38	Molecular
148	FX2_partial covalent interactions	PB	4.13E-01	0.38	Molecular
149	FX2_water bonds	PB	4.13E-01	0.38	Molecular
150	FX1_int_water bonds	PB	4.13E-01	0.38	Molecular
151	CP_TSC	CP	4.30E-01	0.37	Molecular
152	FX1_int_SideHbond	PB	4.31E-01	0.37	Molecular
153	MaxClusterSize	KFC2b	4.38E-01	0.36	Hotspot
154	CoreHSEnergy	KFC2a	4.53E-01	0.34	Hotspot
155	CP_MJ2h	CP	4.60E-01	0.34	Molecular
156	CP_MJ1	CP	4.72E-01	0.33	Molecular
157	CP_VD	CP	4.74E-01	0.32	Molecular
158	Int_Energy_1	RFSpot	4.83E-01	0.32	Hotspot
159	HS_NegCoop	Hotpoint2	4.92E-01	0.31	Hotspot
160	CP_BFKV	CP	4.95E-01	0.31	Molecular
161	SuppHS	RFSpot_KFC2	5.03E-01	0.30	Hotspot
162	FX2_SideHbond	PB	5.11E-01	0.29	Molecular
163	HSEner_NegCoop	KFC2b	5.23E-01	0.28	Hotspot
164	HSEner_PosCoop	KFC2a	5.56E-01	0.25	Hotspot
165	No_Clusters	Hotpoint1	5.63E-01	0.25	Hotspot
166	FX1_int_Electro	PB	5.90E-01	0.23	Molecular
167	RimHSEnergy	RFSpot	6.25E-01	0.20	Hotspot
168	ACE19_SOLVation	PB	6.27E-01	0.20	Molecular
169	CP_MS	CP	6.29E-01	0.20	Molecular
170	SuppHS	Hotpoint1	6.33E-01	0.20	Hotspot
171	FX1_int_Energy_Ionisation	PB	6.33E-01	0.20	Molecular
172	No_HS	RFSpot_KFC2	6.45E-01	0.19	Hotspot
173	Int_Energy_1	Hotpoint1	6.51E-01	0.19	Hotspot
174	Int_Energy_1	Hotpoint2	6.51E-01	0.19	Hotspot
175	Int_HS_Energy	Hotpoint2	6.51E-01	0.19	Hotspot
176	HS_PosCoop	KFC2a	6.65E-01	0.18	Hotspot
177	HS_PosCoop	Hotpoint1	6.72E-01	0.17	Hotspot
178	FX2_disulfide	PB	7.05E-01	0.15	Molecular
179	Int_Energy_1	RFSpot_KFC2	7.15E-01	0.15	Hotspot
180	MaxClusterSize	Hotpoint2	7.41E-01	0.13	Hotspot
181	CP_MJ2	CP	7.57E-01	0.12	Molecular
182	CP_BT	CP	7.70E-01	0.11	Molecular
183	CoreHS	RFSpot_KFC2	7.73E-01	0.11	Hotspot
184	CP_MJ3h	CP	7.77E-01	0.11	Molecular
185	CoreHSEnergy	KFC2b	7.77E-01	0.11	Hotspot
186	SuppHSEnergy	Hotpoint1	7.92E-01	0.10	Hotspot
187	OPUS_PSP3	CP	8.02E-01	0.10	Molecular
188	FX1_int_cis_bond	PB	8.12E-01	0.09	Molecular
189	PY_hbond_sr_bb	PB	8.88E-01	0.05	Molecular
190	PY_hbond_lr_bb	PB	9.01E-01	0.05	Molecular
191	FX2_Electro	PB	9.12E-01	0.04	Molecular
192	ACE19_COULomb	PB	9.29E-01	0.03	Molecular
193	CP_GKS	CP	9.41E-01	0.03	Molecular
194	FX2_Energy_vdwclash	PB	9.48E-01	0.02	Molecular
195	CHARM_total	PB	9.59E-01	0.02	Molecular

64	OPUS_PSP2	1	9.40E-05	4.03	Molecular
65	CP_Qm	1	1.04E-04	3.98	Molecular
66	FX1_int_backbone_vdwclash	3	1.05E-04	3.98	Molecular
67	HSEner_NegCoop	KFC2a	1.66E-04	3.78	Hotspot
68	MaxClusterSize	Hotpoint1	2.07E-04	3.68	Hotspot
69	CHARM_elec	3	2.19E-04	3.66	Molecular
70	HSEner_NegCoop	KFC2b	2.32E-04	3.63	Hotspot
71	CP_BL	1	2.45E-04	3.61	Molecular
72	PY_fa_pair	3	2.71E-04	3.57	Molecular
73	MaxClusterSize	RFSpot_KFC2	3.07E-04	3.51	Hotspot
74	FX2_kn electrostatic	3	3.75E-04	3.43	Molecular
75	No_Clusters	RFSpot_KFC2	3.84E-04	3.42	Hotspot
76	FX1_int_kn electrostatic	3	4.11E-04	3.39	Molecular
77	HS_NegCoop	KFC2b	4.46E-04	3.35	Hotspot
78	HSEner_PosCoop	KFC2b	5.65E-04	3.25	Hotspot
79	SuppHS	RFSpot_KFC2	5.70E-04	3.24	Hotspot
80	DFIRE	2	6.37E-04	3.20	Molecular
81	PY_fa_rep	3	8.08E-04	3.09	Molecular
82	No_HS	RFSpot_KFC2	1.09E-03	2.96	Hotspot
83	CP_MJPL	1	1.24E-03	2.91	Molecular
84	FX2_Entropy_sidec	3	1.28E-03	2.89	Molecular
85	FX1_int_Entropy_sidec	3	1.35E-03	2.87	Molecular
86	SuppHS	KFC2b	1.51E-03	2.82	Hotspot
87	DDFIRE	2	1.71E-03	2.77	Molecular
88	SuppHSEnergy	Hotpoint2	1.82E-03	2.74	Hotspot
89	HSEner_NegCoop	Hotpoint2	1.98E-03	2.70	Hotspot
90	RimHS	Hotpoint1	2.04E-03	2.69	Hotspot
91	MaxClusterSize	KFC2a	2.06E-03	2.69	Hotspot
92	DCOMPLEX	2	2.07E-03	2.68	Molecular
93	CP_RO	1	2.54E-03	2.59	Molecular
94	CoreHS	RFSpot	2.55E-03	2.59	Hotspot
95	CP_SJKG	1	2.63E-03	2.58	Molecular
96	RimHS	RFSpot	3.11E-03	2.51	Hotspot
97	MaxClusterSize	RFSpot	3.17E-03	2.50	Hotspot
98	CoreHSEnergy	KFC2b	3.50E-03	2.46	Hotspot
99	AP_T2	2	4.52E-03	2.34	Molecular
100	CoreHS	KFC2a	4.54E-03	2.34	Hotspot
101	CP_TS	1	5.84E-03	2.23	Molecular
102	ACE19_SASL	3	5.85E-03	2.23	Molecular
103	RimHSEnergy	RFSpot	6.76E-03	2.17	Hotspot
104	NIP	1	7.15E-03	2.15	Molecular
105	CoreHS	RFSpot_KFC2	8.06E-03	2.09	Hotspot
106	FX1_int_ helix dipole	3	9.21E-03	2.04	Molecular
107	CoreHS	Hotpoint2	9.97E-03	2.00	Hotspot
108	PY_fa_intra_rep	3	1.32E-02	1.88	Molecular
109	FX1_int_Energy_vdwclash	3	1.33E-02	1.88	Molecular
110	CP_MSBM	1	1.38E-02	1.86	Molecular
111	CoreHSEnergy	Hotpoint2	1.42E-02	1.85	Hotspot
112	Int_Energy_1	Hotpoint1	1.46E-02	1.84	Hotspot
113	Int_Energy_1	Hotpoint2	1.46E-02	1.84	Hotspot
114	Int_HS_Energy	Hotpoint2	1.46E-02	1.84	Hotspot
115	FX2_Energy_vdwclash	3	1.53E-02	1.82	Molecular
116	No_Clusters	Hotpoint2	1.74E-02	1.76	Hotspot
117	AVG_HS_PathLength	Hotpoint2	1.77E-02	1.75	Hotspot
118	CoreHSEnergy	KFC2a	1.96E-02	1.71	Hotspot
119	OPUS_PSP3	1	2.10E-02	1.68	Molecular
120	Int_Energy_1	RFSpot_KFC2	2.46E-02	1.61	Hotspot
121	SuppHSEnergy	Hotpoint1	3.03E-02	1.52	Hotspot
122	FX1_int_BackHbond	3	3.05E-02	1.52	Molecular
123	HSEner_PosCoop	Hotpoint1	3.35E-02	1.47	Hotspot
124	CHARM_sasa	3	3.60E-02	1.44	Molecular
125	FX2_BackHbond	3	3.61E-02	1.44	Molecular
126	SuppHS	Hotpoint2	4.55E-02	1.34	Hotspot
127	SuppHS	Hotpoint1	4.76E-02	1.32	Hotspot
128	CHARM_total	3	4.77E-02	1.32	Molecular
129	No_Clusters	KFC2b	5.79E-02	1.24	Hotspot
130	CP_MJ2h	1	6.21E-02	1.21	Molecular
131	OPUS_PSP1	1	6.58E-02	1.18	Molecular
132	CP_BT	1	6.64E-02	1.18	Molecular
133	CP_BFKV	1	6.78E-02	1.17	Molecular
134	FX2_ helix dipole	3	7.25E-02	1.14	Molecular
135	CP_TD	1	7.42E-02	1.13	Molecular
136	CP_SKOIP	1	7.42E-02	1.13	Molecular
137	CP_TEs	1	8.34E-02	1.08	Molecular
138	HSEner_PosCoop	KFC2a	8.87E-02	1.05	Hotspot
139	FX1_int_Entropy_mainc	3	8.95E-02	1.05	Molecular
140	FX1_int_Electro	3	9.55E-02	1.02	Molecular
141	CP_TSC	1	1.02E-01	0.99	Molecular
142	CP_GKS	1	1.03E-01	0.99	Molecular
143	CP_TEI	1	1.05E-01	0.98	Molecular
144	CP_MJ1	1	1.06E-01	0.98	Molecular
145	RimHSEnergy	RFSpot_KFC2	1.15E-01	0.94	Hotspot
146	FX2_Entropy_mainc	3	1.20E-01	0.92	Molecular
147	HS_NegCoop	KFC2a	1.24E-01	0.91	Hotspot
148	CP_RMFCEN2	1	1.31E-01	0.88	Molecular
149	FX1_int_Energy_Ionisation	3	1.59E-01	0.80	Molecular
150	RimHS	Hotpoint2	1.60E-01	0.80	Hotspot
151	PY_hbond_bb_sc	3	1.77E-01	0.75	Molecular
152	HS_PosCoop	KFC2b	1.79E-01	0.75	Hotspot
153	PY_pro_close	3	1.88E-01	0.72	Molecular
154	FX2_Electro	3	2.03E-01	0.69	Molecular
155	AP_URS	2	2.10E-01	0.68	Molecular
156	Int_Energy_1	RFSpot	2.17E-01	0.66	Hotspot
157	AP_T1	2	2.19E-01	0.66	Molecular
158	CP_Qa	1	2.36E-01	0.63	Molecular
159	CP_RMFCEN1	1	2.50E-01	0.60	Molecular
160	No_Clusters	KFC2a	2.59E-01	0.59	Hotspot
161	PY_hbond_sc	3	2.60E-01	0.59	Molecular
162	AP_DARS	2	2.73E-01	0.56	Molecular
163	AP_DOPE_HR	2	2.75E-01	0.56	Molecular
164	HS_PosCoop	KFC2a	3.03E-01	0.52	Hotspot
165	No_Clusters	Hotpoint1	3.07E-01	0.51	Hotspot
166	CP_MJ2	1	3.40E-01	0.47	Molecular
167	CHARM_vdwaals	3	3.47E-01	0.46	Molecular
168	CP_MS	1	3.95E-01	0.40	Molecular
169	CP_MJ3h	1	4.75E-01	0.32	Molecular
170	FX2_energy_torsion	3	4.84E-01	0.32	Molecular
171	MaxClusterSize	KFC2b	5.19E-01	0.28	Hotspot
172	FX1_int_energy_torsion	3	5.49E-01	0.26	Molecular
173	HS_PosCoop	Hotpoint1	5.84E-01	0.23	Hotspot
174	FX2_SideHbond	3	5.92E-01	0.23	Molecular
175	FX1_int_disulfide	3	5.95E-01	0.23	Molecular
176	FX2_cis_bond	3	5.95E-01	0.23	Molecular
177	FX1_int_Entropy Complex	3	5.95E-01	0.23	Molecular
178	FX2_Entropy Complex	3	5.95E-01	0.23	Molecular
179	MaxClusterSize	Hotpoint2	6.15E-01	0.21	Hotspot
180	HS_PosCoop	Hotpoint2	6.19E-01	0.21	Hotspot
181	FX2_disulfide	3	6.37E-01	0.20	Molecular
182	FX1_int_SideHbond	3	6.66E-01	0.18	Molecular
183	RimHSEnergy	Hotpoint2	7.78E-01	0.11	Hotspot
184	CP_VD	1	8.26E-01	0.08	Molecular
185	ACE19_SOLVation	3	8.42E-01	0.07	Molecular
186	ACE19_COULomb	3	8.43E-01	0.07	Molecular
187	PY_hbond_lr_bb	3	8.56E-01	0.07	Molecular
188	RimHS	RFSpot_KFC2	8.70E-01	0.06	Hotspot
189	HS_NegCoop	Hotpoint2	8.75E-01	0.06	Hotspot
190	HSEner_PosCoop	Hotpoint2	8.88E-01	0.05	Hotspot
191	FX2_Energy_Ionisation	3	9.49E-01	0.02	Molecular
192	PY_hbond_sr_bb	3	9.63E-01	0.02	Molecular
193	AVG_HS_PathLength	Hotpoint1	NaN	0.00	Hotspot
194	AVG_HS_PathLength	KFC2a	NaN	0.00	Hotspot
195	AVG_HS_PathLength	KFC2b	NaN	0.00	Hotspot

196	HSEner_PosCoop	Hotpoint1	9.66E-01	0.02	Hotspot	196	RimHSEnergy	KFC2b	NaN	0.00	Hotspot
197	AP_DOPE_HR	AP	9.66E-01	0.01	Molecular	197	RimHS	KFC2b	NaN	0.00	Hotspot
198	FX1_int_Energy_vdwclash	PB	9.96E-01	0.00	Molecular	198	AVG_HS_PathLength	RFSpot	NaN	0.00	Hotspot
199	FX1_int_loop_entropy	PB	NaN	0.00	Molecular	199	AVG_HS_PathLength	RFSpot_KFC2	NaN	0.00	Hotspot
200	AVG_HS_PathLength	Hotpoint1	NaN	0.00	Hotspot	200	FX1_int_water bonds	3	NaN	0.00	Molecular
201	AVG_HS_PathLength	KFC2a	NaN	0.00	Hotspot	201	FX1_int_loop_entropy	3	NaN	0.00	Molecular
202	AVG_HS_PathLength	KFC2b	NaN	0.00	Hotspot	202	FX1_int_cis_bond	3	NaN	0.00	Molecular
203	RimHSEnergy	KFC2b	NaN	0.00	Hotspot	203	FX1_int_partial covalent interactions	3	NaN	0.00	Molecular
204	RimHS	KFC2b	NaN	0.00	Hotspot	204	FX2_water bonds	3	NaN	0.00	Molecular
205	AVG_HS_PathLength	RFSpot	NaN	0.00	Hotspot	205	FX2_loop_entropy	3	NaN	0.00	Molecular
206	AVG_HS_PathLength	RFSpot_KFC2	NaN	0.00	Hotspot	206	FX2_partial covalent interactions	3	NaN	0.00	Molecular

Table S8. Area under Curve (AUC) of hotspot and molecular descriptors. Two different data sets are used. Left table uses experimental data from the kinetically characterized mutations in SKEMPI which satisfy Δlog10(koff) <-1 and Δlog10(koff) >0 (CDS1). Right table uses same experimental data but which satisfies |Δlog10(koff)| >1 (CDS2).

Δlog10(koff) <-1 and Δlog10(koff) >0 (CDS1)					|Δlog10(koff)| >1 (CDS2).				
**Rank**	**Descriptor**	**Descriptor Category**	**AUC Stabilizing**	**Descriptor Type**	**Rank**	**Descriptor**	**Descriptor Category**	**AUC Stabilizing**	**Descriptor Type**
1	HS_PosCoop	RFSpot	0.830	Hotspot	1	HSEner_PosCoop	RFSpot	0.949	Hotspot
2	HS_PosCoop	RFSpot_KFC2	0.827	Hotspot	2	HS_PosCoop	RFSpot_KFC2	0.921	Hotspot
3	HSEner_PosCoop	RFSpot	0.825	Hotspot	3	HS_PosCoop	RFSpot	0.916	Hotspot
4	NSc	CP	0.746	Molecular	4	HSEner_PosCoop	RFSpot_KFC2	0.906	Hotspot
5	AP_DOPE	AP	0.742	Molecular	5	Int_HS_Energy	RFSpot	0.878	Hotspot
6	CP_TB	CP	0.738	Molecular	6	Int_HS_Energy	Hotpoint1	0.874	Hotspot
7	AP_MPS	AP	0.732	Molecular	7	CoreHSEnergy	RFSpot	0.872	Hotspot
8	HSEner_PosCoop	RFSpot_KFC2	0.731	Hotspot	8	CoreHSEnergy	RFSpot_KFC2	0.871	Hotspot
9	SuppHSEnergy	KFC2a	0.718	Hotspot	9	Int_HS_Energy	RFSpot_KFC2	0.870	Hotspot
10	RimHSEnergy	KFC2a	0.710	Hotspot	10	CoreHSEnergy	Hotpoint1	0.860	Hotspot
11	No_Clusters	RFSpot	0.709	Hotspot	11	SuppHSEnergy	KFC2a	0.851	Hotspot
12	DFIRE	AP	0.704	Molecular	12	AP_MPS	AP	0.849	Molecular
13	No_HS	KFC2a	0.704	Hotspot	13	SuppHSEnergy	RFSpot_KFC2	0.847	Hotspot
14	Int_HS_Energy	RFSpot	0.699	Hotspot	14	HSEner_NegCoop	Hotpoint1	0.831	Hotspot
15	No_HS	KFC2b	0.695	Hotspot	15	CP_TB	CP	0.818	Molecular
16	ACE19_HYDRophobic	PB	0.694	Molecular	16	No_HS	RFSpot	0.817	Hotspot
17	Int_HS_Energy	RFSpot_KFC2	0.694	Hotspot	17	SuppHSEnergy	RFSpot	0.811	Hotspot
18	FX1_int_Energy_SolvH	PB	0.693	Molecular	18	ACE19_HYDRophobic	PB	0.810	Molecular
19	FX2_Energy_SolvH	PB	0.693	Molecular	19	HS_NegCoop	RFSpot	0.802	Hotspot
20	CoreHSEnergy	RFSpot	0.693	Hotspot	20	No_HS	KFC2b	0.801	Hotspot
21	CoreHSEnergy	RFSpot_KFC2	0.690	Hotspot	21	FX1_int_Energy_VdW	PB	0.796	Molecular
22	CP_SKOb	CP	0.690	Molecular	22	No_HS	KFC2a	0.794	Hotspot
23	GEOMETRIC	CP	0.689	Molecular	23	FX2_Energy_VdW	PB	0.793	Molecular
24	CP_SKOa	CP	0.689	Molecular	24	SuppHSEnergy	KFC2b	0.793	Hotspot
25	CP_RMFCA	CP	0.689	Molecular	25	FX2_Energy_SolvH	PB	0.787	Molecular
26	AP_T2	AP	0.688	Molecular	26	FX1_int_Energy_SolvH	PB	0.787	Molecular
27	No_HS	Hotpoint2	0.685	Hotspot	27	HS_NegCoop	RFSpot_KFC2	0.785	Hotspot
28	PY_fa_dun	PB	0.683	Molecular	28	HSEner_NegCoop	RFSpot	0.784	Hotspot
29	PY_fa_intra_rep	PB	0.682	Molecular	29	CP_Qp	CP	0.782	Molecular
30	DCOMPLEX	AP	0.680	Molecular	30	PY_fa_atr	PB	0.781	Molecular
31	HSEner_NegCoop	Hotpoint1	0.680	Hotspot	31	Int_HS_Energy	KFC2b	0.777	Hotspot
32	FX1_int_Energy_VdW	PB	0.676	Molecular	32	CP_HLPL	CP	0.776	Molecular
33	FX2_Energy_VdW	PB	0.673	Molecular	33	SuppHS	RFSpot	0.776	Hotspot
34	CoreHSEnergy	Hotpoint1	0.671	Hotspot	34	No_HS	Hotpoint1	0.773	Hotspot
35	Int_Energy_1	KFC2b	0.670	Hotspot	35	CP_SKOb	CP	0.772	Molecular
36	FX2_kn electrostatic	PB	0.667	Molecular	36	CP_SKOa	CP	0.764	Molecular
37	CP_Qp	CP	0.666	Molecular	37	Int_Energy_1	KFC2b	0.763	Hotspot
38	PY_fa_atr	PB	0.665	Molecular	38	No_Clusters	RFSpot	0.763	Hotspot
39	FX1_int_kn electrostatic	PB	0.665	Molecular	39	Int_Energy_1	KFC2a	0.762	Hotspot
40	SuppHSEnergy	RFSpot_KFC2	0.664	Hotspot	40	HSEner_NegCoop	RFSpot_KFC2	0.761	Hotspot
41	ACE19_SCREENing	PB	0.661	Molecular	41	AP_DOPE	AP	0.761	Molecular
42	DDFIRE	AP	0.660	Molecular	42	ACE19_SCREENing	PB	0.753	Molecular
43	PY_fa_pair	PB	0.659	Molecular	43	NSc	CP	0.750	Molecular
44	Int_Energy_1	KFC2a	0.657	Hotspot	44	CoreHS	KFC2b	0.750	Hotspot
45	ACE19_INTERaction	PB	0.656	Molecular	45	Int_HS_Energy	KFC2a	0.746	Hotspot
46	Int_HS_Energy	Hotpoint1	0.654	Hotspot	46	CP_RMFCA	CP	0.744	Molecular
47	CP_HLPL	CP	0.651	Molecular	47	ACE19_INTERaction	PB	0.739	Molecular
48	CHARM_elec	PB	0.649	Molecular	48	PY_fa_dun	PB	0.738	Molecular
49	No_Clusters	Hotpoint2	0.647	Hotspot	49	HS_NegCoop	Hotpoint1	0.731	Hotspot
50	FX1_int_ helix dipole	PB	0.647	Molecular	50	GEOMETRIC	CP	0.723	Molecular
51	OPUS_PSP2	CP	0.644	Molecular	51	No_HS	Hotpoint2	0.721	Hotspot
52	NIP	CP	0.644	Molecular	52	SuppHS	KFC2a	0.720	Hotspot
53	No_HS	RFSpot	0.642	Hotspot	53	OPUS_PSP2	CP	0.718	Molecular
54	RimHS	KFC2a	0.642	Hotspot	54	CoreHS	Hotpoint1	0.716	Hotspot
55	CoreHS	KFC2b	0.638	Hotspot	55	CP_Qm	CP	0.716	Molecular
56	FX1_int_BackHbond	PB	0.638	Molecular	56	HSEner_NegCoop	KFC2a	0.709	Hotspot
57	SuppHSEnergy	KFC2b	0.637	Hotspot	57	CHARM_elec	PB	0.706	Molecular
58	HS_NegCoop	RFSpot	0.635	Hotspot	58	HSEner_NegCoop	KFC2b	0.705	Hotspot
59	No_HS	Hotpoint1	0.634	Hotspot	59	MaxClusterSize	Hotpoint1	0.705	Hotspot
60	CP_Qm	CP	0.633	Molecular	60	CP_BL	CP	0.704	Molecular
61	SuppHSEnergy	RFSpot	0.628	Hotspot	61	PY_fa_pair	PB	0.702	Molecular
62	AP_T1	AP	0.627	Molecular	62	FX2_kn electrostatic	PB	0.697	Molecular
63	Int_HS_Energy	KFC2a	0.624	Hotspot	63	MaxClusterSize	RFSpot_KFC2	0.696	Hotspot
64	OPUS_PSP1	CP	0.622	Molecular	64	FX1_int_kn electrostatic	PB	0.696	Molecular
65	SuppHS	KFC2a	0.622	Hotspot	65	HS_NegCoop	KFC2b	0.695	Hotspot
66	No_Clusters	RFSpot_KFC2	0.620	Hotspot	66	HSEner_PosCoop	KFC2b	0.692	Hotspot
67	AVG_HS_PathLength	KFC2a	0.620	Hotspot	67	DFIRE	AP	0.689	Molecular
68	FX2_BackHbond	PB	0.620	Molecular	68	RimHSEnergy	Hotpoint1	0.688	Hotspot
69	CoreHS	Hotpoint1	0.616	Hotspot	69	No_Clusters	RFSpot_KFC2	0.688	Hotspot
70	CP_MSBM	CP	0.615	Molecular	70	SuppHS	RFSpot_KFC2	0.686	Hotspot
71	CP_SJKG	CP	0.614	Molecular	71	No_HS	RFSpot_KFC2	0.680	Hotspot
72	CP_BL	CP	0.613	Molecular	72	CP_MJPL	CP	0.679	Molecular
73	Int_HS_Energy	KFC2b	0.610	Hotspot	73	DDFIRE	AP	0.674	Molecular
74	HSEner_NegCoop	RFSpot	0.610	Hotspot	74	SuppHSEnergy	Hotpoint2	0.673	Hotspot
75	RimHS	RFSpot	0.608	Hotspot	75	HSEner_NegCoop	Hotpoint2	0.672	Hotspot
76	HS_NegCoop	RFSpot_KFC2	0.608	Hotspot	76	DCOMPLEX	AP	0.672	Molecular
77	AVG_HS_PathLength	Hotpoint2	0.607	Hotspot	77	SuppHS	KFC2b	0.668	Hotspot
78	HSEner_NegCoop	KFC2a	0.603	Hotspot	78	CP_SJKG	CP	0.668	Molecular
79	SuppHS	KFC2b	0.603	Hotspot	79	CoreHS	RFSpot	0.664	Hotspot
80	HSEner_NegCoop	RFSpot_KFC2	0.599	Hotspot	80	CoreHSEnergy	KFC2b	0.662	Hotspot
81	No_Clusters	KFC2b	0.598	Hotspot	81	AP_T2	AP	0.658	Molecular
82	HS_NegCoop	KFC2b	0.596	Hotspot	82	MaxClusterSize	RFSpot	0.658	Hotspot
83	CP_MJPL	CP	0.595	Molecular	83	RimHSEnergy	KFC2a	0.656	Hotspot
84	RimHSEnergy	Hotpoint1	0.595	Hotspot	84	CP_TS	CP	0.654	Molecular
85	CHARM_sasa	PB	0.589	Molecular	85	CoreHS	KFC2a	0.653	Hotspot
86	PY_hbond_bb_sc	PB	0.588	Molecular	86	NIP	CP	0.649	Molecular
87	SuppHSEnergy	Hotpoint2	0.586	Hotspot	87	RimHSEnergy	RFSpot	0.648	Hotspot
88	MaxClusterSize	Hotpoint1	0.586	Hotspot	88	CoreHS	RFSpot_KFC2	0.646	Hotspot
89	CP_SKOIP	CP	0.585	Molecular	89	FX1_int_ helix dipole	PB	0.643	Molecular
90	SuppHS	RFSpot	0.583	Hotspot	90	RimHS	KFC2a	0.641	Hotspot
91	CP_TD	CP	0.583	Molecular	91	PY_fa_intra_rep	PB	0.638	Molecular
92	CP_TS	CP	0.583	Molecular	92	CP_MSBM	CP	0.637	Molecular
93	SuppHS	Hotpoint2	0.582	Hotspot	93	CoreHSEnergy	Hotpoint2	0.637	Hotspot
94	CoreHS	KFC2a	0.582	Hotspot	94	Int_Energy_1	Hotpoint1	0.636	Hotspot
95	MaxClusterSize	RFSpot	0.579	Hotspot	95	Int_Energy_1	Hotpoint2	0.636	Hotspot
96	HSEner_NegCoop	Hotpoint2	0.575	Hotspot	96	Int_HS_Energy	Hotpoint2	0.636	Hotspot
97	AP_URS	AP	0.573	Molecular	97	RimHS	RFSpot	0.634	Hotspot
98	CP_RMFCEN1	CP	0.573	Molecular	98	AVG_HS_PathLength	Hotpoint2	0.632	Hotspot
99	HSEner_PosCoop	KFC2b	0.571	Hotspot	99	CoreHSEnergy	KFC2a	0.630	Hotspot
100	FX2_ helix dipole	PB	0.566	Molecular	100	CoreHS	Hotpoint2	0.629	Hotspot
101	RimHS	Hotpoint2	0.561	Hotspot	101	Int_Energy_1	RFSpot_KFC2	0.625	Hotspot
102	RimHS	Hotpoint1	0.560	Hotspot	102	SuppHSEnergy	Hotpoint1	0.623	Hotspot
103	FX1_int_energy_torsion	PB	0.560	Molecular	103	FX1_int_BackHbond	PB	0.621	Molecular
104	CoreHS	RFSpot	0.560	Hotspot	104	RimHS	Hotpoint1	0.618	Hotspot
105	HS_PosCoop	KFC2b	0.560	Hotspot	105	HSEner_PosCoop	Hotpoint1	0.618	Hotspot
106	FX2_energy_torsion	PB	0.558	Molecular	106	No_Clusters	Hotpoint2	0.618	Hotspot

107	AP_DARS	AP	0.556	Molecular
108	CP_RMFCEN2	CP	0.555	Molecular
109	MaxClusterSize	RFSpot_KFC2	0.554	Hotspot
110	CoreHS	Hotpoint2	0.553	Hotspot
111	CHARM_vdwaals	PB	0.553	Molecular
112	CP_Qa	CP	0.551	Molecular
113	CP_TEl	CP	0.550	Molecular
114	HS_NegCoop	KFC2a	0.548	Hotspot
115	CoreHSEnergy	Hotpoint2	0.547	Hotspot
116	HS_NegCoop	Hotpoint1	0.547	Hotspot
117	FX2_Energy_Ionisation	PB	0.545	Molecular
118	FX1_int_SideHbond	PB	0.542	Molecular
119	MaxClusterSize	KFC2b	0.541	Hotspot
120	CoreHSEnergy	KFC2a	0.540	Hotspot
121	No_Clusters	KFC2a	0.540	Hotspot
122	CP_MJ2h	CP	0.539	Molecular
123	CP_BFKV	CP	0.536	Molecular
124	FX2_SideHbond	PB	0.535	Molecular
125	SuppHS	RFSpot_KFC2	0.533	Hotspot
126	HSEner_NegCoop	KFC2b	0.532	Hotspot
127	HSEner_PosCoop	KFC2a	0.531	Hotspot
128	FX1_int_Electro	PB	0.529	Molecular
129	RimHSEnergy	RFSpot	0.525	Hotspot
130	No_HS	RFSpot_KFC2	0.524	Hotspot
131	HS_PosCoop	KFC2a	0.523	Hotspot
132	SuppHS	Hotpoint1	0.522	Hotspot
133	Int_Energy_1	RFSpot_KFC2	0.519	Hotspot
134	CP_BT	CP	0.516	Molecular
135	CP_MJ3h	CP	0.515	Molecular
136	CoreHSEnergy	KFC2b	0.515	Hotspot
137	CoreHS	RFSpot_KFC2	0.515	Hotspot
138	OPUS_PSP3	CP	0.513	Molecular
139	FX1_int_water bonds	PB	0.511	Molecular
140	FX1_int_partial covalent interactions	PB	0.511	Molecular
141	FX2_water bonds	PB	0.511	Molecular
142	FX2_loop_entropy	PB	0.511	Molecular
143	FX2_Electro	PB	0.506	Molecular
144	ACE19_COULomb	PB	0.505	Molecular
145	CHARM_total	PB	0.503	Molecular
146	FX1_int_cis_bond	PB	0.501	Molecular
147	FX1_int_loop_entropy	PB	0.500	Molecular
148	RimHSEnergy	KFC2b	0.500	Hotspot
149	RimHS	KFC2b	0.500	Hotspot
150	FX1_int_Energy_vdwclash	PB	0.498	Molecular
151	HSEner_PosCoop	Hotpoint1	0.498	Hotspot
152	AP_DOPE_HR	AP	0.498	Molecular
153	FX2_Energy_vdwclash	PB	0.497	Molecular
154	CP_GKS	CP	0.496	Molecular
155	PY_hbond_lr_bb	PB	0.495	Molecular
156	PY_hbond_sr_bb	PB	0.495	Molecular
157	SuppHSEnergy	Hotpoint1	0.493	Hotspot
158	FX2_disulfide	PB	0.492	Molecular
159	FX2_partial covalent interactions	PB	0.489	Molecular
160	FX2_cis_bond	PB	0.485	Molecular
161	FX1_int_disulfide	PB	0.484	Molecular
162	FX1_int_Entropy Complex	PB	0.484	Molecular
163	FX2_Entropy Complex	PB	0.484	Molecular
164	CP_MJ2	CP	0.483	Molecular
165	MaxClusterSize	Hotpoint2	0.481	Hotspot
166	FX1_int_Energy_Ionisation	PB	0.481	Molecular
167	HS_PosCoop	Hotpoint1	0.477	Hotspot
168	Int_Energy_1	Hotpoint1	0.476	Hotspot
169	Int_Energy_1	Hotpoint2	0.476	Hotspot
170	Int_HS_Energy	Hotpoint2	0.476	Hotspot
171	CP_MS	CP	0.474	Molecular
172	ACE19_SOLVation	PB	0.474	Molecular
173	No_Clusters	Hotpoint1	0.473	Hotspot
174	PY_pro_close	PB	0.469	Molecular
175	HS_NegCoop	Hotpoint2	0.464	Hotspot
176	Int_Energy_1	RFSpot	0.462	Hotspot
177	CP_VD	CP	0.462	Molecular
178	CP_MJ1	CP	0.461	Molecular
179	CP_TSC	CP	0.458	Molecular
180	FX2_Entropy_mainc	PB	0.455	Molecular
181	RimHSEnergy	RFSpot_KFC2	0.454	Hotspot
182	FX1_int_Entropy_mainc	PB	0.447	Molecular
183	RimHSEnergy	Hotpoint2	0.442	Hotspot
184	PY_fa_rep	PB	0.437	Molecular
185	RimHS	RFSpot_KFC2	0.435	Hotspot
186	PY_hbond_sc	PB	0.416	Molecular
187	HS_PosCoop	Hotpoint2	0.408	Hotspot
188	HSEner_PosCoop	Hotpoint2	0.406	Hotspot
189	AVG_HS_PathLength	RFSpot_KFC2	0.399	Hotspot
190	CP_RO	CP	0.396	Molecular
191	ACE19_SASL	PB	0.384	Molecular
192	FX1_int_backbone_vdwclash	PB	0.374	Molecular
193	PY_fa_sol	PB	0.371	Molecular
194	FX2_backbone_vdwclash	PB	0.370	Molecular
195	FX2_Energy_SolvP	PB	0.363	Molecular
196	CP_TEs	CP	0.360	Molecular
197	MaxClusterSize	KFC2a	0.359	Hotspot
198	FX1_int_Energy_SolvP	PB	0.356	Molecular
199	FX2_Entropy_sidec	PB	0.347	Molecular
200	FX1_int_Entropy_sidec	PB	0.345	Molecular
201	ACE19_SELF	PB	0.341	Molecular
202	CHARM_gb+sasa	PB	0.314	Molecular
203	CHARM_gb	PB	0.299	Molecular
204	AVG_HS_PathLength	Hotpoint1	0.294	Hotspot
205	AVG_HS_PathLength	KFC2b	0.288	Hotspot
206	AVG_HS_PathLength	RFSpot	0.205	Hotspot

107	CHARM_sasa	PB	0.617	Molecular
108	FX2_BackHbond	PB	0.617	Molecular
109	AVG_HS_PathLength	KFC2a	0.611	Hotspot
110	CP_MJ2h	CP	0.604	Molecular
111	SuppHS	Hotpoint2	0.604	Hotspot
112	OPUS_PSP1	CP	0.602	Molecular
113	CP_BFKV	CP	0.602	Molecular
114	CP_BT	CP	0.601	Molecular
115	SuppHS	Hotpoint1	0.600	Hotspot
116	FX2_ helix dipole	PB	0.600	Molecular
117	CP_SKOIP	CP	0.599	Molecular
118	CP_TD	CP	0.599	Molecular
119	No_Clusters	KFC2b	0.597	Hotspot
120	HSEner_PosCoop	KFC2a	0.595	Hotspot
121	FX1_int_Electro	PB	0.593	Molecular
122	CP_TSC	CP	0.591	Molecular
123	CP_GKS	CP	0.590	Molecular
124	CP_TEl	CP	0.590	Molecular
125	HS_NegCoop	KFC2a	0.585	Hotspot
126	RimHSEnergy	RFSpot_KFC2	0.585	Hotspot
127	CP_RMFCEN2	CP	0.584	Molecular
128	PY_hbond_bb_sc	PB	0.575	Molecular
129	HS_PosCoop	KFC2b	0.574	Hotspot
130	FX2_Electro	PB	0.571	Molecular
131	AP_URS	AP	0.570	Molecular
132	Int_Energy_1	RFSpot	0.569	Hotspot
133	AP_T1	AP	0.568	Molecular
134	CP_Qa	CP	0.566	Molecular
135	CP_RMFCEN1	CP	0.564	Molecular
136	AP_DARS	AP	0.561	Molecular
137	RimHS	Hotpoint2	0.559	Hotspot
138	No_Clusters	KFC2a	0.557	Hotspot
139	HS_PosCoop	KFC2a	0.557	Hotspot
140	CP_MJ2	CP	0.552	Molecular
141	CP_MS	CP	0.548	Molecular
142	CP_MJ3h	CP	0.540	Molecular
143	MaxClusterSize	KFC2b	0.535	Hotspot
144	FX2_SideHbond	PB	0.530	Molecular
145	HS_PosCoop	Hotpoint2	0.526	Hotspot
146	FX1_int_SideHbond	PB	0.524	Molecular
147	CP_VD	CP	0.512	Molecular
148	ACE19_COULomb	PB	0.511	Molecular
149	HSEner_PosCoop	Hotpoint2	0.508	Hotspot
150	FX2_disulfide	PB	0.507	Molecular
151	FX1_int_water bonds	PB	0.500	Molecular
152	FX1_int_loop_entropy	PB	0.500	Molecular
153	FX1_int_cis_bond	PB	0.500	Molecular
154	FX1_int_partial covalent interactions	PB	0.500	Molecular
155	FX2_water bonds	PB	0.500	Molecular
156	FX2_loop_entropy	PB	0.500	Molecular
157	FX2_partial covalent interactions	PB	0.500	Molecular
158	RimHSEnergy	KFC2b	0.500	Hotspot
159	RimHS	KFC2b	0.500	Hotspot
160	PY_hbond_sr_bb	PB	0.497	Molecular
161	FX1_int_disulfide	PB	0.495	Molecular
162	FX1_int_Entropy Complex	PB	0.495	Molecular
163	FX2_cis_bond	PB	0.495	Molecular
164	FX2_Entropy Complex	PB	0.495	Molecular
165	FX2_Energy_Ionisation	PB	0.493	Molecular
166	PY_hbond_lr_bb	PB	0.493	Molecular
167	RimHS	RFSpot_KFC2	0.493	Hotspot
168	HS_NegCoop	Hotpoint2	0.490	Hotspot
169	ACE19_SOLVation	PB	0.489	Molecular
170	RimHSEnergy	Hotpoint2	0.485	Hotspot
171	MaxClusterSize	Hotpoint2	0.473	Hotspot
172	HS_PosCoop	Hotpoint1	0.469	Hotspot
173	FX1_int_energy_torsion	PB	0.469	Molecular
174	PY_pro_close	PB	0.463	Molecular
175	FX2_energy_torsion	PB	0.461	Molecular
176	CHARM_vdwaals	PB	0.448	Molecular
177	No_Clusters	Hotpoint1	0.448	Hotspot
178	AP_DOPE_HR	AP	0.439	Molecular
179	PY_hbond_sc	PB	0.437	Molecular
180	FX1_int_Energy_Ionisation	PB	0.433	Molecular
181	FX2_Entropy_mainc	PB	0.413	Molecular
182	CP_MJ1	CP	0.410	Molecular
183	FX1_int_Entropy_mainc	PB	0.405	Molecular
184	CP_TEs	CP	0.404	Molecular
185	CHARM_total	PB	0.390	Molecular
186	AVG_HS_PathLength	RFSpot_KFC2	0.389	Hotspot
187	OPUS_PSP3	CP	0.372	Molecular
188	FX2_Energy_vdwclash	PB	0.365	Molecular
189	FX1_int_Energy_vdwclash	PB	0.363	Molecular
190	ACE19_SASL	PB	0.347	Molecular
191	MaxClusterSize	KFC2a	0.335	Hotspot
192	CP_RO	CP	0.332	Molecular
193	FX1_int_Entropy_sidec	PB	0.321	Molecular
194	FX2_Entropy_sidec	PB	0.321	Molecular
195	PY_fa_rep	PB	0.313	Molecular
196	AVG_HS_PathLength	Hotpoint1	0.295	Hotspot
197	AVG_HS_PathLength	KFC2b	0.288	Hotspot
198	FX1_int_backbone_vdwclash	PB	0.284	Molecular
199	FX2_backbone_vdwclash	PB	0.283	Molecular
200	PY_fa_sol	PB	0.280	Molecular
201	FX2_Energy_SolvP	PB	0.278	Molecular
202	FX1_int_Energy_SolvP	PB	0.266	Molecular
203	CHARM_gb+sasa	PB	0.255	Molecular
204	ACE19_SELF	PB	0.252	Molecular
205	CHARM_gb	PB	0.246	Molecular
206	AVG_HS_PathLength	RFSpot	0.215	Hotspot
